# Integrated omics reveal novel functions and underlying mechanisms of the receptor kinase FERONIA in *Arabidopsis thaliana*

**DOI:** 10.1093/plcell/koac111

**Published:** 2022-04-18

**Authors:** Ping Wang, Natalie M Clark, Trevor M Nolan, Gaoyuan Song, Parker M Bartz, Ching-Yi Liao, Christian Montes-Serey, Ella Katz, Joanna K Polko, Joseph J Kieber, Daniel J Kliebenstein, Diane C Bassham, Justin W Walley, Yanhai Yin, Hongqing Guo

**Affiliations:** Department of Genetics, Development and Cell Biology, Iowa State University, Ames, Iowa 50011, USA; Department of Plant Pathology and Microbiology, Iowa State University, Ames, Iowa 50011, USA; Department of Genetics, Development and Cell Biology, Iowa State University, Ames, Iowa 50011, USA; Department of Plant Pathology and Microbiology, Iowa State University, Ames, Iowa 50011, USA; Department of Genetics, Development and Cell Biology, Iowa State University, Ames, Iowa 50011, USA; Department of Genetics, Development and Cell Biology, Iowa State University, Ames, Iowa 50011, USA; Department of Plant Pathology and Microbiology, Iowa State University, Ames, Iowa 50011, USA; Department of Plant Science, University of California, Davis, California 95616, USA; Department of Biology, University of North Carolina, Chapel Hill, North Carolina 27599, USA; Department of Biology, University of North Carolina, Chapel Hill, North Carolina 27599, USA; Department of Plant Science, University of California, Davis, California 95616, USA; Department of Genetics, Development and Cell Biology, Iowa State University, Ames, Iowa 50011, USA; Department of Plant Pathology and Microbiology, Iowa State University, Ames, Iowa 50011, USA; Plant Sciences Institutes, Iowa State University, Ames, Iowa 50011, USA; Department of Genetics, Development and Cell Biology, Iowa State University, Ames, Iowa 50011, USA; Plant Sciences Institutes, Iowa State University, Ames, Iowa 50011, USA; Department of Genetics, Development and Cell Biology, Iowa State University, Ames, Iowa 50011, USA

## Abstract

The receptor kinase FERONIA (FER) is a versatile regulator of plant growth and development, biotic and abiotic stress responses, and reproduction. To gain new insights into the molecular interplay of these processes and to identify new FER functions, we carried out quantitative transcriptome, proteome, and phosphoproteome profiling of Arabidopsis (*Arabidopsis thaliana*) wild-type and *fer-4* loss-of-function mutant plants. Gene ontology terms for phytohormone signaling, abiotic stress, and biotic stress were significantly enriched among differentially expressed transcripts, differentially abundant proteins, and/or misphosphorylated proteins, in agreement with the known roles for FER in these processes. Analysis of multiomics data and subsequent experimental evidence revealed previously unknown functions for FER in endoplasmic reticulum (ER) body formation and glucosinolate biosynthesis. FER functions through the transcription factor NAI1 to mediate ER body formation. FER also negatively regulates indole glucosinolate biosynthesis, partially through NAI1. Furthermore, we found that a group of abscisic acid (ABA)-induced transcription factors is hypophosphorylated in the *fer-4* mutant and demonstrated that FER acts through the transcription factor ABA INSENSITIVE5 (ABI5) to negatively regulate the ABA response during cotyledon greening. Our integrated omics study, therefore, reveals novel functions for FER and provides new insights into the underlying mechanisms of FER function.

## Teaser

Multi-omics data analysis predicted novel functions of FERONIA in ER body formation and glucosinolate biosynthesis, as well as FERONIA regulation of ABI5 in ABA signaling.

IN A NUTSHELL **Background:** FERONIA (FER) is a plasma membrane-localized receptor kinase that belongs to the CrRLK1L family, which consists of 17 members in Arabidopsis. FER, together with co-receptors LLGs/LRE and ligands such as RALF peptides, plays critical roles in plant growth, stress responses, and reproduction. Better understanding the functions and underlying molecular mechanisms of FER will help illustrate how this receptor kinase balances plant growth and stress in response to environmental cues.

**Question:** We wished to understand the molecular mechanisms underlying FER-mediated biological processes, and to identify novel functions of FER.

**Findings:** Our integrated omics study revealed that FER regulates the expression of thousands of transcripts and the abundance of thousands of proteins, as well as the phosphorylation of more than one thousand proteins. We also uncovered an extensive involvement of transcription factors in FER-mediated signaling. We identified and experimentally validated novel functions for FER in regulating endoplasmic reticulum (ER) body formation and indole glucosinolate biosynthesis. In addition, we established that FER phosphorylates and destabilizes ABI5, an important transcription factor in ABA signaling, to mediate cotyledon greening.

**Next steps:** Our multiomics analysis revealed many potential FER substrates that are involved in diverse biological processes. We will investigate the FER substrates and establish context-specific FER signaling pathways. We will also investigate how FER controls hundreds of transcription factors that regulate thousands of genes for different biological processes.

## Introduction

The receptor kinase FERONIA (FER) plays important roles in plant growth and development, abiotic and biotic stress responses, and reproduction (Franck et al., 2018; Duan et al., 2020; Liu et al., 2021; Ortiz-Morea et al., 2021). Loss-of-function *fer* mutants display skewed male and female interactions and reduced fertility (Escobar-Restrepo et al., 2007; Duan et al., 2020), stunted vegetative growth (Guo et al., 2009b; Chen et al., 2016), collapsed root hairs (Duan et al., 2010), hypersensitivity to high concentrations of NaCl (Chen et al., 2016; Feng et al., 2018; Zhao et al., 2018) and to cold and heat stresses (Chen et al., 2016), susceptibility to bacterial pathogens (Stegmann et al., 2017; Guo et al., 2018), and resistance to fungal pathogens (Kessler et al., 2010; Masachis et al., 2016) and nematodes (Zhang et al., 2020). Most recently, FER was shown to restrict the spread of *Pseudomonas fluorescens* in the rhizosphere, and the loss-of-function *fer-8* mutant displayed increased infection by rhizosphere *P. fluorescens* (Song et al., 2021). FER function is modulated through peptides from the rapid alkalinization factor (RALF) family, which can function as ligands for FER and its co-receptors LORELEI/LORELEI-like glycosylphosphatidylinositol (GPI)-anchored proteins (LRE/LLGs) (Haruta et al., 2014; Li et al., 2015; Stegmann et al., 2017; Guo et al., 2018; Lin et al., 2018, 2021; Xiao et al., 2019). Cell wall pectin can also be recognized by the FER extracellular domain, which mediates salt stress responses and the orientation of microtubules and plays a key role in directing patterns of cellulose biosynthesis (Feng et al., 2018; Lin et al., 2018; Tang et al., 2021). Extracellular proteins such as leucine-rich repeat extensins (LRXs) can interact with FER indirectly through RALFs or directly to modify cell wall expansion and cell growth and salt stress responses (Zhao et al., 2018; Dunser et al., 2019; Herger et al., 2019). FER and its close paralogs, ANXUR1/2, have been shown to form a complex with immune receptors FLAGELLIN-SENSITIVE 2 (FLS2) and EF-TU RECEPTOR (EFR) to facilitate pathogen-associated molecular pattern-triggered immunity (Mang et al., 2017; Stegmann et al., 2017), and FER functions through the guanine exchange factors/Rho GTPases of plants (ROPs) and receptor-like cytoplasmic kinases, RPM1-INDUCED PROTEIN KINASE (RIPK) and MARIS to regulate root-hair development (Duan et al., 2010; Boisson-Dernier et al., 2015; Du et al., 2016). Moreover, FER can directly regulate proteins involved in the control of transcription and translation. FER phosphorylates and destabilizes MYC2, the major transcriptional factor in the jasmonic acid (JA) signaling pathway to negatively regulate JA-mediated host susceptibility (Guo et al., 2018). FER also phosphorylates ERBB-3-binding protein 1 (EBP1), a DNA-binding protein, to promote its nuclear translocation and transcriptional activity (Li et al., 2018). Moreover, FER phosphorylates eukaryotic translation initiation factor 4E1 (eIF4E1) to regulate protein synthesis (Zhu et al., 2020).

FER plays critical roles in plant growth and stress responses. The functional and mechanistic studies of FER can illustrate how a cell surface receptor kinase integrates diverse signals to profoundly influence various cellular pathways and thus balance growth and stress in response to environmental conditions. Studies on FER also provide an important paradigm for a better understanding of receptor kinase signaling in general.

To gain new insights into the functions and mechanisms associated with FER, we globally quantified transcripts, proteins, and phosphorylation sites in wild-type (WT) plants and the *fer-4* mutant. Our study validated many known functions of FER and revealed several interesting novel functions for this receptor kinase. We discovered that FER is involved in endoplasmic reticulum (ER) body formation and glucosinolate biosynthesis. FER functions through the transcription factor NAI1 to negatively mediate ER body formation. FER also negatively regulates indole glucosinolate biosynthesis, partially through NAI1. We also determined that FER phosphorylates and destabilizes abscisic acid (ABA)-INSENSITIVE 5 (ABI5), a critical transcriptional regulator of cotyledon greening (Guan et al., 2014), which provides a novel mechanism underlying the negative role of FER in ABA signaling. Taking advantage of the comprehensive integrated omics analysis along with genetics, cell biology, and biochemistry experiments, our study reveals novel functions for FER and provides new insights into the underlying mechanisms of FER function.

## Results

### Integrated omics confirm known functions and predict novel functions of FER 

We performed multiomics analyses on 4.5-week-old Arabidopsis rosette leaves from the WT Columbia-0 (Col-0) and *fer-4* (hereafter *fer*) grown under long-day conditions (see “Materials and methods”). Under our growth conditions, 4.5-week-old *fer* plants showed a pronounced stunted growth phenotype with smaller rosettes, while 10-day-old *fer* seedlings exhibited an overall similar growth to that of WT (Supplemental Figure S2, C and D). Transcriptome profiling by 3′-based RNA sequencing (QuantSeq) identified 3,908 (*q* < 0.05) transcripts that are differentially expressed in *fer* (Supplemental Figure S1), of which 2,299 had increased levels and 1,609 decreased levels in the mutant compared to WT (Figure�1A;Supplemental Data Set 1). More than 50% of the 3,908 genes (2,190/3,908) were also identified as differentially expressed in *fer* mutants in our previous report (Guo et al. 2018; Supplemental Figure S2B). We determined that 4,129 (*q* < 0.05) proteins have different abundance in the *fer* mutant out of 8,621 quantified proteins (i.e. protein groups; Supplemental Figure S1). Among the differentially abundant proteins (DAPs), 2,346 had increased and 1,783 had decreased levels in the *fer* mutant (Figure�1A;Supplemental Data Set 1). Finally, we detected 11,955 phosphosites from 2,959 proteins (i.e. protein groups), among which 3,432 phosphosites (*q* < 0.2) had altered phosphorylation status in the mutant background relative to the WT (Supplemental Figure S1). Further, 1,577 phosphosites (from 703 different proteins) had elevated phosphorylation, and 1,855 phosphosites (from 691 different proteins) exhibited decreased phosphorylation, with 110 proteins displaying both elevated and decreased phosphorylation levels at different sites (Figure�1A;Supplemental Data Set 1). Comparisons among the three sets of omics data revealed that 29% of all DAPs also exhibit differential expression of their cognate transcripts in *fer*, with most of the DAPs following the same direction of change as their transcript (Figure�1B;Supplemental Figure S2A). Similarly, 47% of the differentially phosphorylated proteins also showed an alteration at the corresponding transcript or protein levels (Figure�1B;Supplemental Figure S2A).

**Figure 1:**
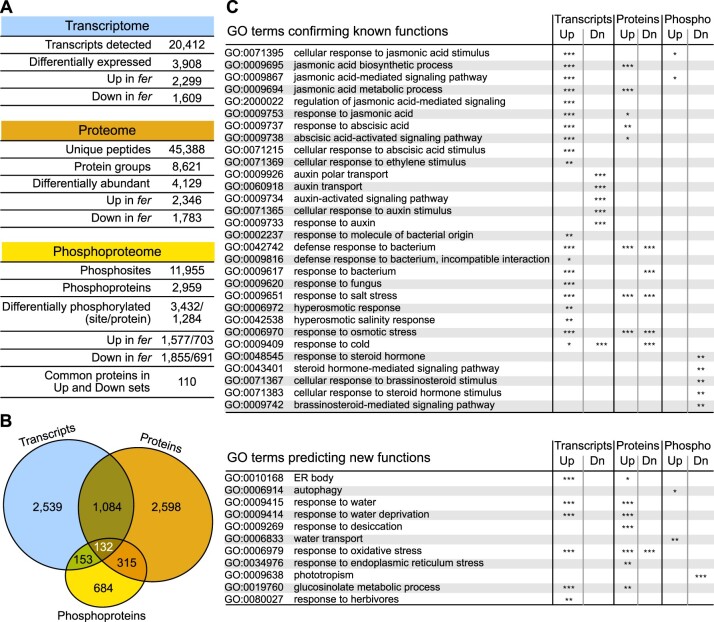
Integrated omics confirm known functions and predict novel functions of FER. A, Summary table of the results from transcriptome, proteome, and phosphoproteome analyses. The number of identified transcripts, peptides, protein groups, phosphorylation sites, and phosphorylated proteins in this study as well as the number of differentially expressed genes or DAPs or differentially phosphorylated proteins in the *fer-4* mutant are shown. B, Venn diagram showing the extent of overlap between differentially expressed transcripts, DAPs, and differentially phosphorylated proteins. C, Selected GO terms that corroborate the known functions of FER, and those that predict novel functions for FER. GO enrichment analysis was performed using the ClueGO application in Cytoscape (Bindea et al., 2009). Terms with corrected *P* < 0.05 were considered enriched, and the *P*-values are indicated with stars (*P < 0.05; **P < 0.01; ***P < 0.001).

We performed gene ontology (GO) analysis using ClueGO in Cytoscape on our multiomics data (Bindea et al., 2009) (Supplemental Figures S3–S8; Supplemental Data Set 2). We considered GO terms with a corrected *P* < 0.05 as significantly enriched. The GO analysis validated many previous findings concerning FER function in plants. Namely, GO terms related to stress phytohormones were enriched (Figure�1C;Supplemental Data Set 2). For example, GO terms such as response to JA (GO:0009753), response to ABA (GO:0009737), and ethylene (GO:0071369), were significantly enriched among transcripts and/or proteins with increased levels in *fer*, which corroborated the previous findings that FER regulates ABA, JA, and ethylene signaling pathways and that loss-of-function *fer* mutants are hypersensitive to these phytohormones (Deslauriers and Larsen, 2010; Yu et al., 2012; Guo et al., 2018). Additionally, GO terms related to growth-promoting phytohormones were enriched (Figure�1C). Consistent with a role for FER in brassinosteroid (BR)-mediated plant growth, the GO terms response to BR (GO:0009741) and BR-mediated signaling pathway (GO:0009742) were enriched among proteins with decreased phosphorylation levels (Guo et al., 2009b). Providing support for the findings that FER is involved in auxin-regulated processes, GO terms such as auxin polar transport (GO:0009926), auxin activated signaling pathway (GO:0009734), and response to auxin (GO:0009733) were also enriched among transcripts with lower levels in *fer* (Duan et al., 2010; Figure�1C;Supplemental Figures S3–S8; Supplemental Data Set 2).

We also observed the enrichment of GO terms related to various biotic stresses, for example, defense response to bacterium (GO:0042742), and defense response to fungus (GO:0050832; Figure�1C;Supplemental Data Set 2), which confirmed the involvement of FER in fungal and bacterial defense responses (Masachis et al., 2016; Stegmann et al., 2017; Guo et al., 2018)*.* Similarly, GO terms related to abiotic stresses were enriched, for example, response to cold (GO:0009409), response to salt stress (GO:0009651), and response to osmotic stress (GO:0006970; Figure�1C;Supplemental Data Set 2). Consistent with our findings, FER has been experimentally shown to regulate responses to cold stress, salt stress, and osmotic stress imposed by mannitol. In agreement, the loss-of-function *fer* mutant is hypersensitive to cold and high salt and is resistant to osmotic stress (Chen et al., 2016; Feng et al., 2018; Zhao et al., 2018).

Moreover, the analysis of the omics data predicted previously unknown functions for FER (Figure�1C;Supplemental Data Set 2). Among these, ER body (GO:0010168) and glucosinolate metabolism (GO:0019760) were enriched, suggesting that FER is involved in ER body formation and glucosinolate metabolism.

Using the hypophosphorylated proteins in the *fer* mutant that are potential FER substrates, we predicted the consensus sequences of the FER phosphorylation sites using the motifeR software (Wang et al., 2019a), which identified 33 significantly enriched consensus sequences of FER phosphorylation motifs (Supplemental Data Set 3). The LOGOs of the three most enriched sequences are shown in Supplemental Figure S9A. Furthermore, we carried out an in vitro kinase assay to validate potential FER substrates. We selected nine proteins for this assay, each harboring at least one of the 33 enriched FER phosphorylation motifs. These proteins have diverse functions, including transcription factors (ABA-RESPONSIVE ELEMENTS-BINDING FACTOR3 [ABF3], OXIDATIVE STRESS2 [OXS2], FLOWERING BHLH3 [FBH3], ABI5, and GT2), a plasma membrane-localized protein (SHOU4) involved in cellulose biosynthesis (Polko et al., 2018), a cytoplasmic protein (NAIP1, At3g51950) involved in ER body formation (Wang et al., 2019b) and a nucleus/chloroplast-localized protein (NUCLEAR SHUTTLE INTERACTING, NSI). The identified phosphopeptides and phosphorylation sites in these proteins are shown in Figure�2A. We performed the above in vitro kinase assays with FER kinase (FERK, consisting of the FER C-terminus containing the kinase domain) fused to glutathione S-transferase (GST) as described (Guo et al., 2018), with the nine candidate substrate proteins fused to maltose-binding protein (MBP). The kinase assays revealed that seven of the nine proteins are phosphorylated in the presence of FERK (Figure�2B), suggesting that FER can directly phosphorylate these proteins.

**Figure 2:**
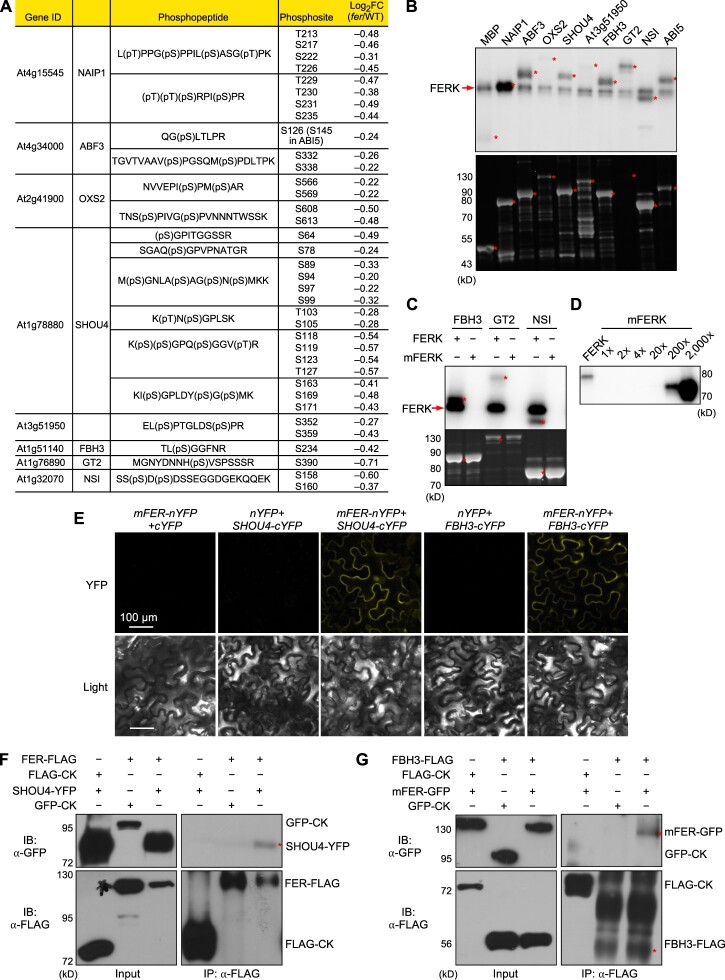
FER directly phosphorylates many proteins with diverse functions. A, List of proteins selected for in vitro kinase assays, with gene identifier, phosphopeptide(s), phosphosites, and the change in phosphorylation levels in *fer*, as indicated by Log_2_(fold-change[*fer*/WT]). B, In vitro kinase assay of the selected proteins. Top, autoradiograph of the in vitro kinase assay, showing FERK autophosphorylation (red arrow) and phosphorylation of the selected proteins (asterisks). Note that NAIP1 phosphorylation overlaps with FER autophosphorylation. Bottom, SYPRO RUBY staining of the gel with the same amount of proteins run separately. C, In vitro kinase assay of FBH3, GT2, and NSI using a similar amount of FERK and FERK^K565R^ (mFERK). D, Autophosphorylation of FERK and different amounts of mFERK. 1� mFERK indicates the same amount as FERK. Note the change in mobility of FERK^K565R^ compared to FERK when resolved on 8% SDS–PAGE. E, Protein-protein interaction by BiFC in *N. benthamiana* leaves. Fluorescence and light images of leaf epidermal cells are shown. Scale bars = 100 �m. F–G, Co-IP assays confirming the interactions of FER with SHOU4 (F) and FBH3 (G). Constructs were co-infiltrated in *N. benthamiana*. Proteins were immunoprecipitated with anti-FLAG (mouse) and detected with anti-FLAG (rabbit) and anti-GFP (rabbit) antibodies.

The K565R (lysine 565 to arginine) mutation in the FER kinase domain (mFERK) greatly decreases FER kinase activity (Escobar-Restrepo et al., 2007; Haruta et al., 2018). To confirm that FERK itself is responsible for the phosphorylation of these substrates in the in vitro kinase assay, we repeated the assay with FERK and mFERK (K565R) using three of the substrate proteins, FBH2, GT2, and NSI. FERK showed both autophosphorylation and phosphorylation of its putative substrates, mFERK did not show appreciable levels of autophosphorylation or phosphorylation of any of these substrates when provided in amounts equal to those used for FERK (Figure�2C). Interestingly, a larger amount of mFERK (i.e. approximately 200 times more than FERK) showed autophosphorylation in an in vitro kinase assay (Figure�2D), suggesting residual kinase activity in this mutant. We estimated the amount of FERK and mFERK used for kinase assay by immunoblotting using an anti-FER antibody (Supplemental Figure S9B; Guo et al., 2018). It is worth noting that the mFERK appears to migrate faster than FERK when resolved on 8% (w/v) sodium dodecyl-sulfate polyacrylamide gel electrophoresis (SDS–PAGE) (Supplemental Figure S9B).

We also tested the potential for interaction between FER and its putative substrates using bimolecular fluorescence complementation (BiFC) assays in transiently infiltrated *Nicotiana benthamiana* leaves. FER interacted with SHOU4, FBH3, and ABI5 (Figure�2E;Supplemental Figure S9C). DAWDLE (DDL), a mostly nucleus-localized protein that is not among the proteins detected in the phosphoproteomics data, was used as a negative control (Supplemental Figure S9D). We confirmed the interaction between FER and the substrates SHOU4 and FBH3 by co-immunoprecipitation (Co-IP) assays in *N. benthamiana* leaves transiently infiltrated with constructs encoding tagged versions of each protein (Figure�2, F and G). The Co-IP did not detect an interaction between FER and ABI5, which might be due to a highly transient interaction and/or highly unstable FER-phosphorylated ABI5.

Taken together, our multiomics data analysis validated previous findings that FER plays important roles in plant growth and development, abiotic and biotic stress responses. The phosphoproteomics detected many FER substrates in planta. We further validated some of the FER substrates using in vitro kinase assays, BiFC, and Co-IP. The multiomics data analysis also predicted novel functions for FER in ER body formation and glucosinolate biosynthesis, as well as FER regulation of ABI5 in ABA signaling. As detailed in the next sections, we explored and experimentally validated the role of FER in these processes.

### FER negatively regulates ER body formation

The ER body is a type of membranous structure, 1 � 10 �m in size, that is contiguous with the ER and is surrounded by ribosomes in the Arabidopsis cytoplasm (Hayashi et al., 2001). ER bodies, specific to Brassicales, are constitutively present in epidermal cells of healthy young seedlings but absent in rosette leaves of healthy WT plants (Hayashi et al., 2001). NAI1, a basic helix-loop-helix (bHLH) transcription factor, is required for ER body formation (Matsushima et al., 2003a, 2004) and a loss-of-function *nai1* mutant lacks ER bodies even in young seedlings. Many ER body-localized or body-related proteins are known (Matsushima et al., 2003b), and the expression of many of the genes encoding these proteins is regulated by NAI1 (Yamada et al., 2008).

Consistent with the enrichment of the GO term for ER body (GO:0010168) (Figure�1C;Supplemental Data Set 2), 16 out of the 17 known ER body-associated genes (listed in Figure�3A; Wang et al., 2017) displayed increased transcript levels, and 14 of the16 had increased protein abundance in the *fer* mutant background compared to WT (Figure�3A), suggesting that FER negatively regulates ER body formation. Sixteen of the 17 genes also exhibited increased transcript levels in the *fer* mutant in a previously described transcriptome dataset (*fer*-DEs-CB in Figure�3A) (Guo et al., 2018). We also generated an additional set of QuantSeq transcriptome data from 10-day-old seedlings for WT and the *fer* mutant and identified 7,718 differentially expressed transcripts (2,718 of them downregulated and 5,018 upregulated, respectively, in *fer* relative to WT) (Supplemental Data Set 4). All 17 ER body genes showed increased transcript levels (Figure�3A). A volcano plot also revealed the significant alterations in abundance for their encoded proteins (Figure�3B). We confirmed the expression pattern of three selected ER body genes by reverse transcription–quantitative polymerase chain reaction (RT-qPCR) in 3-week-old plants (Figure�3E). Consistent with the transcriptome data from 4.5-week-old plants and 10-day-old seedlings, *NAI1*, *NAI2*, and *PYK10* transcripts accumulated to higher levels in *fer* relative to WT. *NAI2* and *PYK10* were expressed at lower levels in the *nai1* mutant, corroborating the previous findings that NAI1 activates *NAI2* and *PYK10* transcription (Yamada et al., 2008). To better understand the genetic interaction between *FER* and *NAI1*, we constructed the *fer nai1* double mutant. Interestingly, the expression of *NAI2* and *PYK10* decreased in the *fer nai1* double mutant compared to the *fer* single mutant, suggesting that FER regulates the expression of ER body genes through NAI1. It is worth noting that the *nai1* mutant (CS69075) we used in this study has a point mutation (Matsushima et al., 2004) that does not disrupt the transcription of the mutant allele or its regulation by FER (Figure�3E).

**Figure 3:**
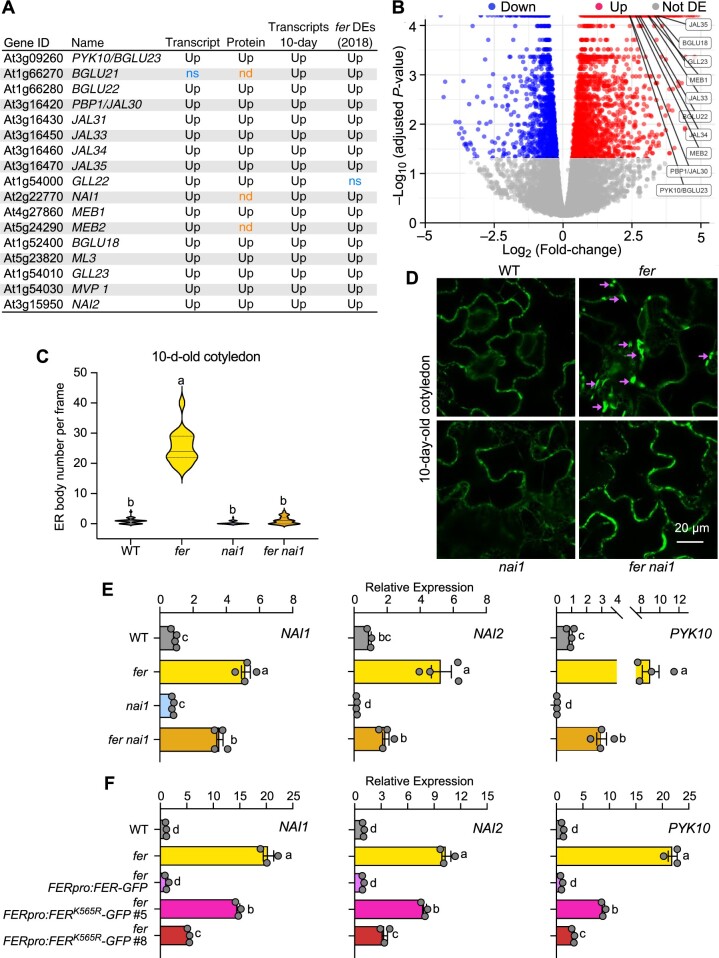
FER negatively regulates ER body formation. A, List of ER body-associated genes (Wang et al., 2017) and their regulation in the differentially expressed transcripts and DAPs in 4.5-week-old *fer* plants, differentially expressed transcripts in 10-day-old *fer* seedlings (Transcripts 10 days) and previously published *fer* transcriptome data (*fer* DEs (2018)) (Guo et al., 2018). Up: increased levels in *fer* mutant. NS: detected, but no statistically significant differences. ND: not detected. B, Volcano plot constructed with the 8,621 detected proteins, with ggplot2 (Wickham, 2016) in R using –Log_10_-transformed q-values and Log_2_(Fold-change) of protein abundance. The *x*-axis represents Log_2_(Fold-change) of protein levels between *fer* and WT; the *y*-axis represents statistical significance using –Log_10_(adjusted *P*-value). Black, no statistically significant changes in *fer*; blue, decreased levels in *fer*; red, increased levels in *fer.* Selected ER body-associated proteins are indicated. C, Number of ER bodies in 10-day-old cotyledons, quantified from confocal images of the four genotypes overexpressing the ER marker *GFP-HDEL*. WT (*n* = 31), *fer* (*n* = 18), *nai1* (*n* = 31), and *fer nai1* (*n* = 28). Data are shown as violin plots with median, first and third quartiles. Different letters indicate significant differences according to one-way analysis of variance (ANOVA)Tukey’s multiple range tests (*P* < 0.05). D, Representative confocal microscopy images from (C). Scale bar = 20 �m. E, RT-qPCR of representative ER body-related genes (*NAI1, NAI2*, and *PYK10*) in 3-week-old rosette leaves of WT, *fer, nai1*, and *fer nai1* overexpressing *GFP-HDEL*. F, RT-qPCR of *NAI1, NAI2*, and *PYK10* in 3-week-old WT, *fer*, *fer FERpro:FER-GFP*, or *fer FERpro:FER^K565R^-GFP* complementation lines. Relative expression levels were normalized to those of the reference gene *ACTIN2*. qPCR was performed with two to three technical replicates of three to four independent biological replicates. Data represent means � sem. Different letters indicate significant differences according to one-way ANOVA Tukey’s multiple range tests (*P* < 0.05).

To visualize ER bodies in plants, we crossed the ER marker *GFP-HDEL* (encoding the green fluorescent protein [GFP] with the ER retention signal HDEL) into the *fer* mutant background. We observed ER bodies in 5- and 10-day-old seedlings and 3-week-old rosette leaves of *fer GFP-HDEL* (Figure�3, C–D;Supplemental Figure S10, A–C). We used 5-day-old seedlings, in which ER bodies are abundant, and 10-day-old seedlings, in which ER bodies are largely diminished in WT cotyledons expressing *GFP-HDEL*, in the rest of this study. In 5-day-old seedlings, we observed ER bodies in the cotyledons, hypocotyls, and roots of both *GFP-HDEL* and *fer GFP-HDEL*, while we detected no ER bodies in *nai1 GFP-HDEL* except for a very few in roots (Supplemental Figure S10, B and C). We counted more ER bodies in the cotyledons and roots of *fer GFP-HDEL* compared to the *GFP-HDEL* line, likely due to increased *NAI1* expression in the *fer* mutant (Supplemental Figure S10, B and  C). While we failed to observe ER bodies in the cotyledons of 10-day-old *GFP-HDEL* seedlings (Figure�3, C and D), we did detect many in the cotyledons of *fer GFP-HDEL* (Figure�3, C and D), suggesting that FER negatively regulates ER body formation in older cotyledons and rosette leaves (Figure�3, C–D;Supplemental Figure S10A).

NAI1 was more highly expressed in *fer* relative to WT (Figure�3, A and E). To test our hypothesis that FER regulates ER body by negatively regulating NAI1, we generated the *fer nai1* double mutant harboring the *GFP-HDEL* reporter, yielding *fer nai1 GFP-HDEL*. Similar to *nai1 GFP-HDEL*, 5-day-old *fer nai1 GFP-HDEL* seedlings lacked ER bodies (Supplemental Figure S10, B and C). The increased ER body phenotype observed in 10-day-old *fer GFP-HDEL* also diminished in the *fer nai1 GFP-HDEL* background (Figure�3, C and D), suggesting that FER functions through NAI1 to regulate ER body formation.

To further support a role for FER in ER body formation, we transformed an artificial microRNA (amiRNA) specific for *FER* (*FER-*amiRNA) into the *GFP-HDEL* reporter line to generate the *FER-*amiRNA *GFP-HDEL* line. The *FER-*amiRNA has previously been shown to effectively knock down endogenous *FER* transcripts, with the knockdown plants behaving similarly to *fer* mutants for both plant growth and bacterial defense in T_2_ transgenic lines (Guo et al., 2009b, 2018). In *FER-*amiRNA *GFP-HDEL* T_2_ lines, FER protein abundance was much lower than in the *GFP-HDEL* line (Supplemental Figure S11A). We counted more ER bodies in 10-day-old cotyledons in all three *FER-*amiRNA *GFP-HDEL* T_2_ lines tested here (lines #2, #4, and #8; Supplemental Figure S11, D and E). *FER-*amiRNA *GFP-HDEL* lines also exhibited a slight increase in the number of ER bodies in 5-day-old cotyledons compared to the *GFP-HDEL* line (Supplemental Figure S11, B and C), similar to *fer GFP-HDEL* (Supplemental Figure S10, B and C). In summary, these results suggest that FER negatively regulates ER body formation by negatively regulating NAI1.

Complementation assays with full-length intact *FER* and mutant *FER^K565R^* constructs revealed that FER kinase activity is not required for some FER-mediated processes, such as ovule fertilization (Kessler et al., 2015), but was for other processes, such as stomatal movement, vegetative growth, and root elongation (Chakravorty et al., 2018; Haruta et al., 2018). Chakravorty et al. further showed that high levels of FER^K565R^ protein can complement the *fer* dwarf growth phenotype and restore stomatal movements regulated by the ligand RALF1. We thus investigated if ER body regulation by FER requires its kinase activity. From our transcriptome data and RT-qPCR results, we observed that the expression pattern of ER body genes is a good indicator of ER body phenotype. We measured the expression of ER body genes in WT, *fer*, and complementation lines harboring an intact *FER* or *FER^K565R^* transgene (*fer FERpro:FER-GFP*, *fer FERpro:FER^K565R^-GFP* #5 and *fer FERpro:FER^K565R^-GFP* #8; Shih et al., 2014; Chakravorty et al., 2018). Consistent with their reported growth phenotypes, the *FERpro:FER* transgene fully complemented the ER body gene expression pattern seen in *fer*. The lines *FERpro:FER^K565R^-GFP* #5 and #8 showed partial restoration, with line #8, with higher levels of the FER^K565R^-GFP fusion protein, showing greater rescue than line #5 (Figure�3F). These results suggest that FER regulation of ER body formation is dependent on its kinase activity.

NAI2 is an ER body protein found only in Brassicales that is involved in ER body formation and function; loss-of-function *nai2* mutants largely lack ER bodies (Yamada et al., 2008; Wang et al., 2019b). NAI1, the major transcription factor in ER body formation, was shown to activate *NAI2* gene expression by directly binding to its promoter (Sarkar et al., 2021). To further support the conclusion that FER functions through NAI1 to regulate ER body formation, we generated *FER-*amiRNA *nai2-2 GFP-HDEL* lines, in which FER levels dropped relative to *nai2-2* (Figure�4A). Similar to *nai2-2 GFP-HDEL*, all three individual *FER-*amiRNA *nai2-2 GFP-HDEL* lines lacked ER bodies in the cotyledons of both 5- and 10-day-old seedlings, while *FER-*amiRNA *GFP-HDEL* showed ER body formation in both 5- and 10-day-old seedlings (Figure�4, B–E). These results further demonstrate that FER negatively regulates NAI1 and hence NAI2 to control ER body formation.

**Figure 4:**
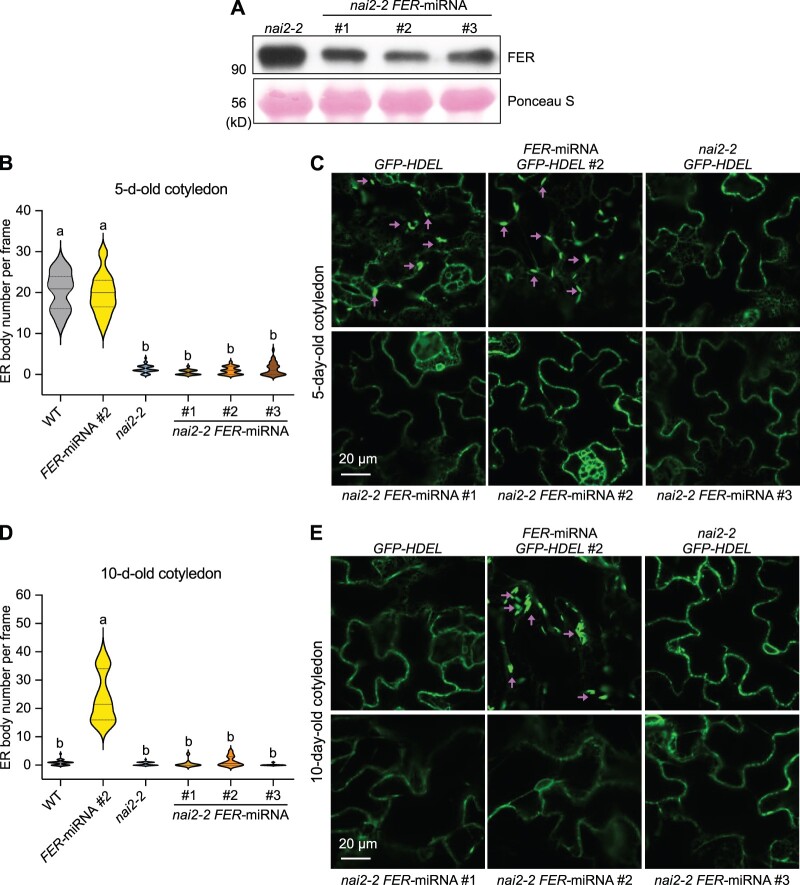
NAI2 functions downstream of FER in ER body formation. A, Immunoblot showing the decreased FER protein levels in the three lines of *FER-*amiRNA *nai2-2* used for the ER body analysis. Ponceau S staining of Rubisco is shown as loading control. B and D, Number of ER bodies in 5-day-old (B) and 10-day-old (D) cotyledons from the controls (*GFP-HDEL, FER-*amiRNA *GFP-HDEL #2*, and *nai2-2 GFP-HDEL*) and three individual lines of *FER-*amiRNA *nai2-2 GFP-HDEL* (#1, #2, and #3). Data are shown as violin plots with median, first and third quartiles. Different letters indicate significant differences according to one-way ANOVA Tukey’s multiple range tests (*P* < 0.05). *n* = 11–35. C and E, Representative confocal images from 5-day-old (C) and 10-day-old (E) cotyledons. Scale bars = 20 �m.

### FER negatively regulates indole glucosinolate biosynthesis

Glucosinolates are a family of secondary metabolites specific to Brassicales order that play important roles in responses to biotic stresses such as insect herbivory (Burrow et al., 2010). The two major forms of Arabidopsis glucosinolates are aliphatic glucosinolates (AG) synthesized from methionine, and indolic glucosinolates (IG) synthesized from tryptophan (S�nderby et al., 2010), and their levels can be induced by JA treatment (Kliebenstein et al., 2002). Consistent with the enrichment of the GO term glucosinolate metabolism (GO:0019760) in our omics data (Figure�1C;Supplemental Data Set 2), the transcript abundance and the levels of the encoding proteins of many IG biosynthetic genes and the genes that are involved in both IG and AG biosynthesis increased in *fer* relative to WT (Figure�5, A and B). Consistent with the increased gene expression, we observed a rise in the levels of three different IGs in the *fer* mutant, indol-3-ylmethyl (I3M), 4-methoxy-indol-3-ylmethyl (4MOI3M), and 1-methoxy-indol-3-ylmethyl (1MOI3M; Figure�5, C–E). Furthermore, while we did not see significant changes in the levels of I3M or 4MOI3M in the *nai1* mutant compared to WT, their levels did decrease in the *fer nai1* double mutant compared to those measured in *fer*, suggesting that NAI1 functions downstream of FER to promote the biosynthesis of I3M and 4MOI3M. Disruption of NAI1 did not alter 1MOI3M biosynthesis (Figure�5E), consistent with previous observations that 1MOI3M is regulated independently from other IGs (Kliebenstein et al., 2002).

**Figure 5:**
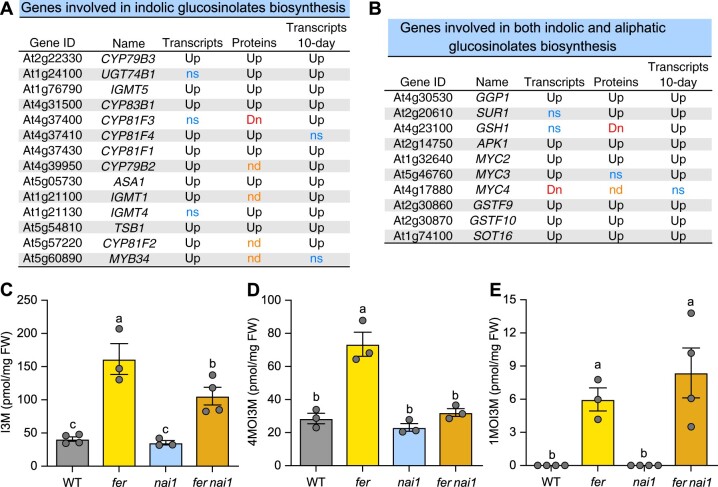
FER negatively regulates indole glucosinolate biosynthesis, partially through NAI1. A, Summary of gene expression and protein accumulation patterns involved in indole glucosinolate biosynthesis in the transcriptome and proteome data of 4.5-week-old *fer plants* and transcriptome data of 10-day-old *fer* seedlings (Transcripts 10 days). B, Summary of gene expression and protein accumulation patterns involved in both indole and aliphatic glucosinolate biosynthesis in our transcriptome and proteome data. For (A) and (B), Up: increased levels in the *fer* mutant. Down: decreased levels in *fer* mutant. NS: detected but no statistically significant differences. C–E, Mean contents for the three IGs, I3M, 4MOI3M, and 1MOI3M in the four genotypes, WT, *fer*, *nai1*, and *fer nai1*, given as pmol/mg fresh whole rosettes (3-week-old). Data represent means � sem from three to four biological replicates. Different letters indicate significant differences according to one-way ANOVA Tukey’s multiple range tests (*P* < 0.05).

To further strengthen the correlation between IG levels and the expression levels of IG genes, we carried out RT-qPCR in 3-week-old plants for six genes involved in IG biosynthesis: *ANTHRANILATE SYNTHASE ALPHA SUBUNIT1* (*ASA1*), *INDOLE GLUCOSINOLATE O-METHYLTRANSFERASE5* (*IGMT5*), *MYC2*, *CYTOCHROME P450 83B1* (*CYP83B1*)*, CYP79B2*, and *CYP79B3*. Consistent with the increased IG levels in *fer*, all six genes displayed increased transcript levels in *fer* (Figure�6A). While we observed no major changes in the expression of these six genes in the *nai1* mutant, the expression of *MYC2* decreased in the *fer nai1* double mutant, suggesting that NAI1 is required for optimal *MYC2* expression and also supports the observation that I3M and 4MOI3M levels are lower in *fer nai1* compared to *fer.* We also performed RT-qPCR using the *FERpro:FER-GFP* and *FERpro:FER^K565R^-GFP* complementation lines. While *FERpro:FER-GFP* completely restored the expression of all six genes back to WT levels, we witnessed little to partial rescue in *FERpro:FER^K565R^-GFP* #5 and partial to complete rescue in *FERpro:FER^K565R^-GFP* #8 (Figure�6B). These results demonstrated that optimal FERK activity is important for the regulation of IG biosynthesis.

**Figure 6:**
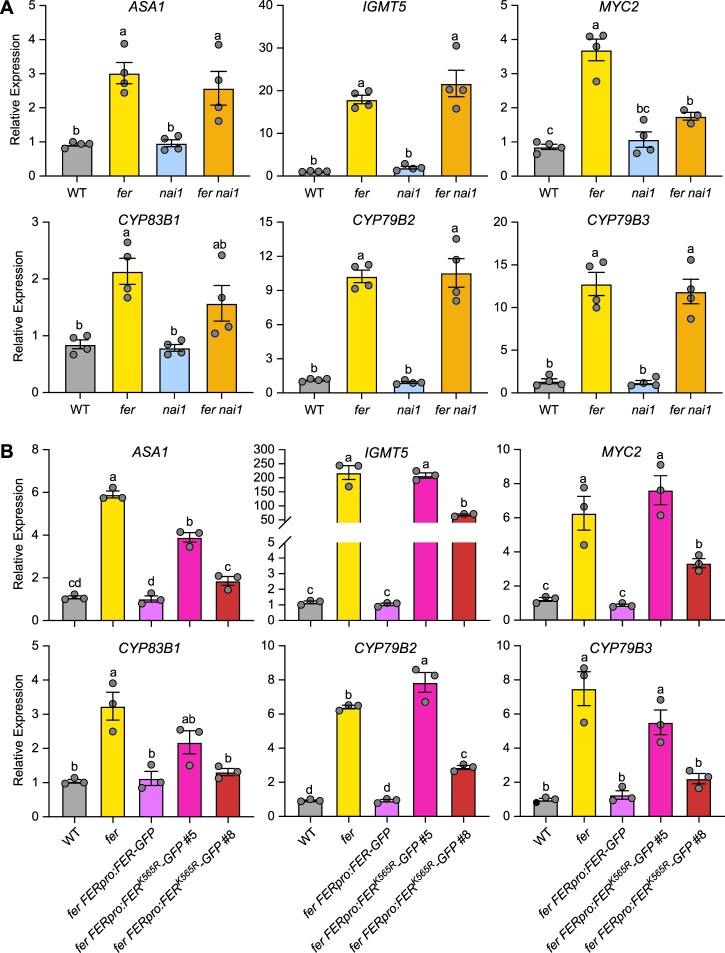
FER negatively regulates IG biosynthetic gene expressions. A and B, RT-qPCR of selected genes involved in IG biosynthesis (*ASA1*, *IGMT5*, *MYC2*, *CYP83B1*, *CYP79B2*, and *CYP79B3*) in 3-week-old rosette leaves of WT, *fer, nai1*, and *fer nai1* overexpressing *GFP-HDEL* (A) and in 3-week-old WT, *fer*, *fer FERpro:FER-GFP*, or *fer FERpro:FER^K565R^-GFP* complemented lines (B). Relative expression levels were normalized to those of the reference gene *ACTIN2*. qPCR was performed on two to three technical replicates of three to four independent biological replicates. Data represent means � sem. Different letters indicate significant differences according to one-way ANOVA Tukey’s multiple range tests (*P* < 0.05).

Taken together, our results suggest that FER negatively regulates indole glucosinolate biosynthesis while inhibiting ER body formation, which strongly corroborates the previous findings of co-regulation between ER body-related genes and glucosinolates biosynthesis and catabolic genes (Nakano et al., 2017; Wang et al., 2017). Further, these results suggest that FER functions as a potential link between environmental signals and ER body formation/IG biosynthesis in response to stress conditions.

### FER negatively regulates ABA signaling during cotyledon greening through ABI5

FER has been shown to negatively regulate ABA signaling by interacting with ABI2 (Yu et al., 2012; Chen et al., 2016). An interesting finding from our phosphoproteomics analysis was that a group of TFs whose transcription is induced by ABA, including ABF1 (At1g49720), ABF2 (At1g45249), ABF3 (At4g34000), ABF4 (At3g19290), ABA-RESPONSIVE ELEMENT BINDING PROTEIN3 (AREB3; At3g56850), ENHANCED EM LEVEL (EEL; At2g41070), and FBH3 (At1g51140; Song et al., 2016) are hypophosphorylated in *fer*, suggesting that FER regulates their phosphorylation. Direct target genes were reported for ABF1, ABF2, ABF3, and FBH3 from chromatin immunoprecipitation deep sequencing (ChIP-seq) studies (Song et al., 2016). We observed a significant overlap between *fer*-regulated genes and the target genes of these ABA-related TFs (Supplemental Figure S12A; Supplemental Data Set 5), suggesting that FER can function through these TFs to regulate ABA signaling.

Among the peptides that are hypophosphorylated in *fer*, we mapped the phosphopeptide QG(pS)LTLPR to ABF1, ABF2, ABF3, ABF4, AREB3, and EEL. Further examination revealed that the phosphorylated serine residue and seven out of the eight amino acids in the peptide are also conserved in ABI5, a transcription factor involved in ABA-mediated cotyledon greening (Figure�7A), raising the possibility that FER regulates ABA-mediated cotyledon greening through ABI5. ABI5 can be phosphorylated at many sites by protein kinases such as SNF1-RELATED PROTEIN KINASE2 (SnRK2), BR-INSENSITIVE2 (BIN2), and PROTEIN KINASE SOS2-LIKE5 (PKS5, also named CBL-INTERACTING PROTEIN KINASE11 [CIPK11]), and the kinase(s) responsible for phosphorylating Ser-145 in ABI5 has yet to be identified (Lopez-Molina et al., 2002; Yu et al., 2015). To test the hypothesis that FER regulates ABI5 to regulate cotyledon greening, we generated the *fer abi5-7* double mutant. In the cotyledon greening assay, while *fer* was hypersensitive and *abi5-7* was resistant to 1-�M ABA treatment, as quantified by the percentage of seedlings with green cotyledons, the *fer abi5-7* double mutant showed a level of tolerance to ABA similar to that of *abi5-7*, suggesting that FER represses ABI5 function during cotyledon greening (Figure�7, B and C;Supplemental Figure S12B). Further analysis of the cotyledon greening with complementation *fer* lines showed that *FERpro:FER-GFP* can largely rescue the ABA hypersensitivity of *fer.* The complementation by *FERpro:FER^K565R^-GFP* was partial, and the degree of the complementation positively correlated with FER^K565R^-GFP protein abundance, as measured with an anti-GFP antibody (#5 < #8 < #6) (Figure�7, D and E; Chakravorty et al., 2018), which demonstrates that FER regulation of ABA-mediated cotyledon greening is dependent on its kinase activity.

**Figure 7:**
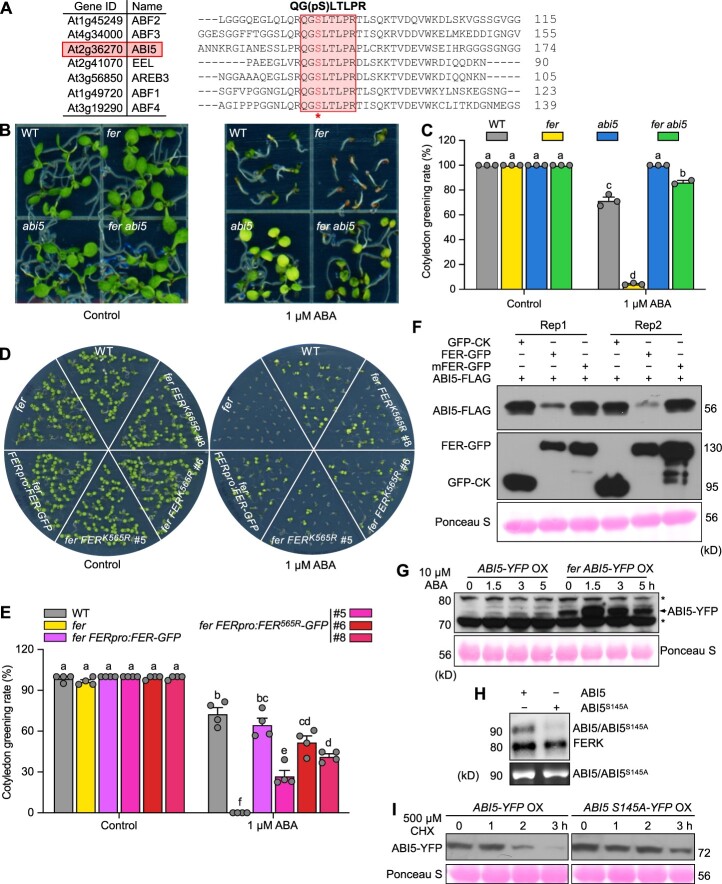
FER negatively regulates ABA response during cotyledon greening through ABI5. A, The phosphopeptide QG(pS)LTLPR is shown here, along with a partial alignment between ABI5 and six other ABA-responsive transcription factors in which the phosphopeptide was identified. B, Cotyledon greening assay of WT, *fer*, *abi5-7*, and *fer abi5-7*, on control half-strength MS medium or half-strength MS medium containing 1 �M ABA. Images are of 5-day-old seedlings. C, Quantification of cotyledon greening rates from (B) with 30 seeds of each genotype per treatment. Data represent means � sem from three biological replicates. Different letters indicate significant differences according to one-way ANOVA Tukey’s multiple range tests (*P* < 0.05). D, Cotyledon greening assay of WT, *fer*, *fer FERpro:FER-GFP*, or *fer FERpro:FER^K565R^-GFP* complementation lines on control half-strength MS medium or half-strength MS medium containing 1 �M ABA. Images are of 5-day-old seedlings. E, Quantification of cotyledon greening rates from (D) with 40 seeds of each genotype per treatment. Data represent means � sem from four biological replicates. Different letters indicate significant differences according to one-way ANOVA Tukey’s multiple range tests (*P* < 0.05). F, Co-infiltration of *ABI5-FLAG* with *FER-GFP* or *FER^K565R^* (*mFER-GFP*) in *N. benthamiana* for 2 days. Proteins were detected by immunoblotting with anti-FLAG (rabbit) and anti-GFP (rabbit) antibodies. Ponceau S staining of Rubisco protein serves as loading control. Data from two independent leaves are shown here. G, Immunoblot showing ABI5 protein abundance upon treatment of *ABI5-YFP* OX and *fer ABI5-YFP OX* with 10 �M ABA. Asterisk indicates background bands. Arrow indicates ABI5-YFP. Ponceau S staining of Rubisco protein serves as loading control. H, In vitro kinase assay showing the phosphorylation of ABI5 and decreased phosphorylation of ABI5^S145A^ and FERK autophosphorylation (top); bottom, SYPRO RUBY staining of the proteins used in the assay. I, Immunoblot showing the stability of ABI5 and ABI5^S145A^ upon CHX treatment, in transgenic plants overexpressing *ABI5-YFP* or *ABI5^S145A^-YFP*. Ponceau S staining of Rubisco protein serves as loading control.

To elucidate the potential mechanisms by which FER regulates ABI5, we co-infiltrated the constructs *ABI5-FLAG* and *FER-GFP*, *mFER-GFP* (*FER^K565R^-GFP*), or *GFP* control into *N. benthamiana* leaves. Immunoblots established the lower accumulation of ABI5 when *ABI5-FLAG* was co-infiltrated with *FER-GFP*, compared to the *GFP* control (Figure�7F), suggesting that FER negatively regulates ABI5 protein levels. *mFER-GFP* did not appreciably change ABI5 protein abundance, suggesting that the negative regulation of ABI5 by FER is kinase dependent. We further generated *ABI5-yellow fluorescent protein* (*YFP*) transgenic plants by introducing a transgene consisting of the *ABI5* coding sequence cloned in-frame and upstream of *YFP* and driven by the cauliflower mosaic virus 35S promoter (*35Spro:ABI5-YFP* OX). We then crossed the resulting transgenic lines to *fer* and generated *fer 35Spro:ABI5-YFP* OX lines (Supplemental Figure S12C). To test the effect of FER on ABI5 stability, we treated the rosette leaves of *35Spro:ABI5-YFP* OX and *fer 35Spro:ABI5-YFP* OX with 10 �M ABA. While we detected low levels of ABI5-YFP in *ABI5-YFP* OX prior to ABA treatment, we observed a modest increase in ABI5-YFP abundance after ABA treatment (Figure�7G). In contrast, we noticed the greater accumulation of ABI5-YFP in *fer ABI5-YFP* OX lines even before ABA treatment, which further increased upon ABA treatment. These results suggest that FER negatively regulates ABI5 protein stability (Figure�7G).

An in vitro kinase assay showed that ABI5 can be directly phosphorylated by FER (Figure�2C). We thus mutated Ser-145 in ABI5 to Ala to produce the nonphosphorylatable variant ABI5^S145A^. In vitro kinase assay determined that FER phosphorylation of ABI5^S145A^ is greatly reduced compared to that of intact ABI5, suggesting that FER phosphorylates ABI5 at S145 (Figure�7H). Furthermore, we generated transgenic Arabidopsis lines overexpressing *ABI5^S145A^-YFP* (*ABI5^S145A^-YFP* OX). ABI5^S145A^-YFP appeared to be more stable than ABI5-YFP when seedlings were treated with the protein synthesis inhibitor cycloheximide (CHX; Figure�7I), suggesting that FER destabilizes ABI5 through phosphorylation at S145 in planta. Similar to *ABI5-YFP* OX seedlings, *ABI5^S145A^-YFP* OX lines were also hypersensitive to ABA during cotyledon greening (Supplemental Figure S12D). Taken together, our results indicate that FER negatively regulates ABA responses during cotyledon greening through phosphorylation and destabilization of ABI5, in addition to the known regulation of ABI2 by FER (Yu et al., 2012; Chen et al., 2016).

## Discussion

The receptor kinase FER plays critical roles in mediating plant growth and development, as well as responses to biotic and abiotic stress. Our integrated omics analysis of the *fer-4* mutant not only corroborated previous findings but also revealed new pathways and potential underlying mechanisms of FER function (Figure�8). We showed here that FER negatively regulates ER body formation by regulating the transcription factor NAI1, along with the protein encoded by its target gene *NAI2*. FER also repressed indole glucosinolate biosynthesis, which was consistent with the previously observed co-occurrence of ER body formation and IG biosynthesis and catabolism (Nakano et al., 2017; Wang et al., 2017). In addition, our phosphoproteomics analysis identified a group of TFs whose encoding genes are induced by ABA and whose phosphorylation is regulated by FER and likely act downstream of FER. We also showed that ABI5 functions downstream of FER and is phosphorylated at the S145 residue by FER, leading to ABI5 destabilization. Thus, our integrated omics study provided new insights into FER functions and underlying molecular mechanisms.

**Figure 8:**
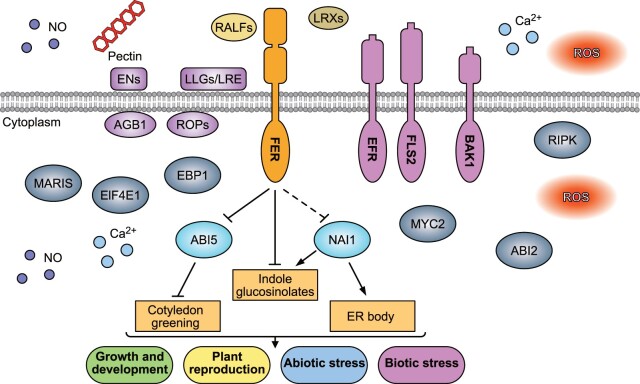
Known and newly identified functions of FER and underlying mechanisms. FER and its co-receptors LLGs/LRE can perceive RALF peptides as ligands (Haruta et al., 2014; Li et al., 2015; Xiao et al., 2019) to regulate diverse biological processes, such as female fertility (Escobar-Restrepo et al., 2007; Hou et al., 2016; Duan et al., 2020), plant growth and development (Guo et al., 2009a; Duan et al., 2010), abiotic and biotic stress responses (Kessler et al., 2010; Chen et al., 2016; Masachis et al., 2016; Stegmann et al., 2017; Feng et al., 2018; Guo et al., 2018; Zhao et al., 2018). Many molecules and FER-interacting proteins are also involved in FER signaling. These include extracellular pectin, reactive oxygen species (ROS), Ca^2+^ and nitric oxide (NO), and LRX proteins (Duan et al., 2014; Ngo et al., 2014; Feng et al., 2018; Lin, 2018; Zhao et al., 2018; Dunser et al., 2019; Duan et al., 2020); plasma membrane-associated proteins such as early nodulin-like proteins, Arabidopsis G-protein beta subunit 1, ROPs, FLS2, EFR, and BRI1-associated receptor kinase (Duan et al., 2010; Hou et al., 2016; Stegmann et al., 2017; Yu et al., 2018); intracellular proteins such as ABI2, RIPK, MARIS, MYC2, EBP1, eIF4E1, and intracellular ROS, Ca^2+^, and NO (Yu et al., 2012; Boisson-Dernier et al., 2015; Du et al., 2016; Guo et al., 2018; Li et al., 2018; Zhu et al., 2020). The ligand RALF1 functions through FER to inhibit root elongation (Haruta et al., 2014); FER also interacts with and activates ROP2 to promote root hair development (Duan et al., 2010); FER controls pectin and NO to contribute to female reproduction (Duan et al., 2020); LRXs interact with FER directly or through RALFs to mediate vacuolar expansion or salt tolerance, respectively (Zhao et al., 2018; Dunser et al., 2019); and upon bacterial pathogen attack, FER can serve as a scaffold for FLS2/EFR or phosphorylates and destabilizes MYC2 to promote immune response (Stegmann et al., 2017; Guo et al., 2018). In our current multiomics study, we identified and validated novel functions for FER and underlying molecular mechanisms. FER negatively regulates ER body and indolic glucosinolate biosynthesis through the negative regulation of the transcription factor NAI1, and positively regulates cotyledon greening through the negative regulation of ABI5.

Although ER bodies have been implicated in stress responses, how they are regulated by signaling pathways remains largely unknown. Unlike young seedlings with many ER bodies, the rosette leaves of adult Arabidopsis plants and older seedlings are largely free of ER bodies (Hayashi et al., 2001; Wang et al., 2017). The fact that the *fer* mutant retains ER bodies in older seedlings and adult leaves indicated that FER negatively regulates ER body formation. We further determined that FER inhibits ER body formation through the negative regulation of NAI1, a master transcription factor controlling ER body-related gene expression such as the ER body resident gene *NAI2*. ER body formation can also be induced by wounding and phytohormones such as JA in Arabidopsis (Hara-Nishimura and Matsushima, 2003; Geem et al., 2019; Stefanik et al., 2020). We showed previously that FER negatively regulates the JA signaling pathway by phosphorylating and destabilizing MYC2, the master transcription factor in the JA pathway (Guo et al., 2018). MYC2 and its homologs MYC3 and MYC4 activate the expression of the ER body gene *TRYPTOPHAN SYNTHASE ALPHA CHAIN1*, which is involved in JA-induced ER body formation in Arabidopsis adult leaves (Stefanik et al., 2020), which suggests that FER can also regulate ER body formation by regulating the JA signaling pathway. This study thus provides an important link between FER-mediated signaling pathway and ER body formation, and a platform to study the mechanisms by which internal and external stimuli are integrated through the receptor kinase FER to regulate ER body formation and plant stress responses.

Glucosinolates are a group of secondary metabolites that exert important functions in plant responses to biotic stresses (Burrow et al., 2010). ER body-related genes and glucosinolate biosynthetic and catabolic genes are strongly co-expressed, and ER body formation co-occurs with indole glucosinolates (Nakano et al., 2017; Wang et al., 2017). ER bodies are known to accumulate beta-glucosidases such as PYK10 and BGLU21 that are involved in the hydrolysis of glucosinolates and defense against herbivores such as woodlice (*Armadillidium vulgare*), thereby influencing Arabidopsis/endophyte interactions (Sherameti et al., 2008; Nakazaki et al., 2019; Yamada et al., 2020). In the *fer* mutant, ER body formation is constitutive with increased levels of beta-glucosidases, as well as elevated indole glucosinolates, which suggests a role for FER in herbivory and other biotic interactions. Future studies should help establish the functions of FER and underlying molecular mechanisms in plant–biotic interactions mediated by ER body and glucosinolates.

Our study further highlights that FER regulates diverse biological processes through the regulation of transcription factors. In addition to our previous finding that FER regulates MYC2 to modulate JA signaling and plant immunity (Guo et al., 2018), here we discovered that (1) FER negatively regulates NAI1 in controlling ER body formation and partially controlling IG biosynthesis, likely through intermediate proteins (Figures�3–6) and (2) that FER phosphorylates and destabilizes ABI5 to control cotyledon greening (Figure�7). In addition, we looked for all 2,492 transcription factors encoded by the Arabidopsis genome (Pruneda-Paz et al., 2014) among differentially expressed transcripts, DAPs, and phosphoproteins in *fer*. This analysis showed that more than 20% of the transcription factors (524/2,492) exhibit altered levels for their transcripts, encoded proteins, or phosphorylation state (Supplemental Figure S13; Supplemental Data Set 6), which strongly suggests that FER regulates diverse biological processes through the extensive involvement of multiple transcription factors, directly or indirectly. Future analysis of these transcription factors should reveal a more comprehensive FER signaling network and provide a better understanding of the molecular interplay in FER-mediated signaling pathways.

## Materials and methods

### Plant materials and growth conditions

The Arabidopsis accession Col-0 was used as WT in all experiments. The T-DNA insertion mutant *fer-4* (GABI-106A06, referred to as *fer*) and the *nai1-1 GFP-HDEL* mutant (CS69075) were described previously (Matsushima et al., 2004; Guo et al., 2018). Seeds for the *nai2-2* (SALK_005896) mutant harboring the *GFP-HDEL* transgene (Yamada et al., 2008) were generously provided by Dr. Kenji Yamada. *Abi5-7* seeds (Nambara et al., 2002) were provided by Dr. Yan Bao. *GFP-HDEL* seeds were described previously (Batoko et al., 2000). The *fer* complementation lines *fer FERpro:FER-GFP* and *fer FERpro:FER^K565R^-GFP* (lines #5, #6, and #8) (Shih et al., 2014; Chakravorty et al., 2018) were generously provided by Prof. Sarah M. Assmann. For all experiments involving Arabidopsis plants, seeds were surface sterilized with 70% (v/v) ethanol containing 0.1% (v/v) Triton X-100 and sown on half-strength Murashige and Skoog (MS) medium with 1% (w/v) sucrose and 0.8% (w/v) agar, with or without treatments as indicated. Ten-day-old seedlings were transferred to soil at 22�C under long-day (16-h light/8-h dark) conditions with a light intensity of ∼120–150 μmol m^−2^s^−1^.

### Quantitative proteomics and phosphoproteomics

#### Protein extraction and digestion

The proteomics experiments were carried out based on established methods as follows (Walley et al., 2018; Song et al., 2018a, 2018b, 2020). Three biological replicate samples were collected from 4.5-week-old entire rosettes from both Col-0 and the *fer* mutant, with 4–5 Col-0 rosettes and 8–10 *fer* rosettes per replicate. Lysis buffer consisting of 8 M urea, 100-mM Tris–HCl pH 7, 5-mM tris(2-carboxyethyl)phosphine (TCEP) and 1 � phosphatase inhibitor (2.5-mM NaF, 0.25-mM NaVO_4_, 0.25- mM sodium pyrophosphate decahydrate, and 0.25-mM glycerophosphate in H_2_O) was added to 250-mg tissue at a ratio of 1:2 sample:buffer (w:v). Zirconium oxide beads (1-mm diameter, Next Advance) were added to the samples at a 1:1 ratio (v:v) and then the samples were shaken using a GenoGrinder (SPEX) at 1,500 rpm for 3 min. The samples were centrifuged at 4,000 *g* for 3 min. The shaking and centrifugation steps were repeated once. Samples were transferred to new tubes, to which four volumes of prechilled 100% acetone were added. Samples were precipitated at −20�C for >30 min followed by centrifugation at 4,500 *g* for 10 min at 4�C. Acetone (80%, v/v) was added to the pellets and the samples were probe-sonicated to resuspend the pellet and shear DNA. Samples were incubated at –20�C for >5 min and then centrifuged at 4,500 *g* for 10 min at 4�C. Precipitation and sonication in 80% (v/v) acetone was performed 3 times in total. Prechilled 100% methanol was then added to each pellet, the samples were probe-sonicated, and kept at –20�C for 30 min prior to centrifugation at 4,500 *g* for 10 min at 4�C. Methanol precipitation was repeated once. The supernatant was discarded and the pellet was placed in a vacuum concentrator until nearly dry.

Proteins were solubilized in 0.5-mL protein resuspension buffer (8 M urea, 0.1-M Tris–HCl pH 7.0, 5-mM TCEP, 1 � phosphatase inhibitor) and probe-sonicated. The protein amount was evaluated by Bradford assay and ∼1 mg was mixed with 3.5-mL urea solution (8 M urea, 0.1-M Tris–HCl pH 8.0, 1 � phosphatase inhibitor). This solution was added to an Amicon Ultracel—30K centrifugal filter (Cat # UFC803008) and centrifuged at 4,000 *g* for 20–40 min at room temperature. This step was repeated once. Then 4 mL of urea solution with 2-mM TCEP was added to the filter unit and centrifuged at 4,000 *g* for 20–40 min. Next, 2-mL iodoacetamide (IAM) solution (50-mM IAM in 8-M urea) was added and incubated without mixing at room temperature for 30 min in the dark prior to centrifugation at 4,000 *g* for 20–40 min. Two milliliters of 8-M urea were added to the filter unit, which was then centrifuged at 4,000 *g* for 20–40 min. This step was repeated once. Then, 2 mL of 0.05 M NH_4_HCO_3_ with 1 � phosphatase inhibitor was added to the filter unit and centrifuged at 4,000 *g* for 20–40 min. This step was repeated once. Then the filter unit was added to a new collection tube and 2 mL of 0.05 M NH_4_HCO_3_ with 1 � phosphatase inhibitor with trypsin (enzyme to protein ratio 1:100, w/w) was added. Samples were incubated at 37�C overnight. Undigested proteins were estimated using Bradford assays before adding trypsin (1 μg/μL) at a ratio of 1:100 (w/w) and an equal volume of Lys-C (0.1 μg/μL) was added and incubated for an additional 4 h at 37�C. The filter unit was centrifuged at 4,000 *g* for 20–40 min. One milliliter of 0.05-M NH_4_HCO_3_ with 1 � phosphatase inhibitor was added and centrifuged at 4,000 *g* for 20–40 min. The samples were acidified to pH 2–3 with 100% formic acid and centrifuged at 21,000 *g* for 20 min. Finally, samples were desalted using 50 mg Sep-Pak C18 cartridges (Waters, Milford, MA, USA). Eluted peptides were dried using a vacuum centrifuge (Thermo Fisher Scientific, Waltham, MA, USA) and resuspended in 0.1% (v/v) formic acid. Peptide amount was quantified using the Pierce BCA Protein assay kit.

#### TMT labeling

TMTsixplex label reagents (Thermo Fisher Scientific, Lot #SH254566) were used to label the samples according to the manufacturer’s recommended peptide-to-tandem mass tag (TMT) reagent ratio. Four hundred micrograms of vacuum-dried peptides from each sample were resuspended with 400-�L 50 mM TEAB buffer and vortexed for 10 min at room temperature. Then, 41-�L acetonitrile was added to each tube of TMT label (0.8 mg), vortexed, and incubated at room temperature for 5 min to resuspend the labels. Four tubes (4 � 41 �L) of each type of TMT label (3.2-mg TMT label in total) were added to each tube of peptides (400 �g), pipetted up and down several times, and vortexed to mix them well. After a 2-h incubation at room temperature, 32 �L of 5% (w/v) hydroxylamine was added to each tube, vortexed, and incubated at room temperature for 15 min to quench the labeling reaction. Next, the six samples were mixed together, a 35-�g aliquot of peptides was reserved for protein abundance profiling, and the remaining peptides were used for phosphopeptide enrichment, and stored at –80�C.

#### Phosphopeptide enrichment

The TMT-labeled phosphopeptides were enriched using Titansphere Phos-TiO_2_ beads (GL Sciences 5010–21315) based on previously published methods (Kettenbach and Gerber, 2011; Song et al., 2018b). The beads were prepared by resuspending in 1.5-mL wash and binding buffer (2 M lactic acid in 50% [v/v] acetonitrile), vortexing, and then centrifugation at 3,000 *g* for 1 min at room temperature; this was repeated a total of 3 times. At the last washing step, 5 and 11 mg of TiO_2_ beads were aliquoted into new tubes before centrifugation. After centrifugation, the wash and binding buffer were removed and the TiO_2_ beads were saved for phosphopeptide enrichment. About 2.4 mg of TMT6-labeled and vacuum-dried peptides were resuspended with 2.4 mL wash and binding buffer and then added to the tube containing 11 mg TiO_2_ beads, rotated at room temperature for 1 h, and then centrifuged at 3,000 *g* for 1 min at room temperature. The supernatant was processed with a second round of enrichment using 5 mg of TiO_2_ beads. Then, 1.8--mL wash and binding buffer was added to each tube from the two enrichment steps, vortexed, and centrifuged at 3,000 *g* for 1 min at room temperature. This wash was repeated once. Next, the TiO_2_ beads were washed twice with 1.8 mL of 50% (v/v) acetonitrile in 0.1% (w/v) trifluoroacetic acid. After the wash steps, 500 �L of 3% (w/v) ammonium hydroxide was added to each tube of the two enrichment steps, vortexed and centrifuged at 3,000 *g* for 1 min at room temperature. The eluted supernatants were combined. One more elution step was performed with 5% (w/v) ammonium hydroxide. All supernatants from the two elution steps were combined and dried in a speed vac; the phosphopeptides were then resuspended in 0.1% (w/v) FA, and stored at –80�C until liquid chromatography-tandem mass spectrometry (LC/MS–MS) run.

#### LC/MS–MS

An Agilent 1,260 quaternary high-performance liquid chromatograph (HPLC) was used to deliver a flow rate of ∼600 nL min^−1^ via a splitter. All columns were packed in-house using a Next Advance pressure cell, and the nanospray tips were fabricated using a fused silica capillary that was pulled to a sharp tip using a laser puller (Sutter P-2000). Thirty-five micrograms of TMT-labeled peptides (nonmodified proteome), or 25 �g TiO_2_-enriched peptides (phosphoproteome), were loaded onto 20-cm capillary columns packed with 5-μM Zorbax SB-C18 (Agilent Technologies, Inc., St Clara, CA, USA), which was connected using a zero dead volume 1-μm filter (Upchurch, M548) to a 5-cm long strong cation exchange (SCX) column packed with 5-μm PolySulfoethyl (PolyLC). The SCX column was then connected to a 20-cm nanospray tip packed with 2.5 μM C18 (Waters). The three sections were joined and mounted on a custom electrospray source for online nested peptide elution. A new set of columns was used for each sample. Peptides were eluted from the loading column onto the SCX column using a 0%–80% (v/v) acetonitrile gradient over 60 min. Peptides were then fractionated from the SCX column using a series of salt steps. For the nonmodified proteome, the following ammonium acetate salt steps were used (in millimolar): 10, 25, 30, 32, 33, 34, 35, 36, 37, 38, 39, 40, 42, 45, 47, 50, 55, 65, 75, 90, 98, 100, 110, 130, 150, 200, and 1,000. For the phosphoproteome analysis, ammonium acetate steps of 6, 10, 12, 15, 18, 21, 30, 45, 70, 90, 100, 150, 500, and 1,000 mM were used. For these analyses, buffers A (99.9% [v/v] H_2_O, 0.1% [v/v] formic acid), B (99.9% [w/v] acetonitrile [I], 0.1% [v/v] formic acid), C (100 mM ammonium acetate, 2% [w/v] formic acid), and D (2 M ammonium acetate, 2% [w/v] formic acid) were utilized. For each salt step, a 150-min gradient program consisted of a 0–5 min increase to the specified ammonium acetate concentration, 5–10 min hold, 10–14 min at 100% buffer A, 15–120 min 5%–35% buffer B, 120–140 min 35%–80% buffer B, 140–145 min 80% buffer B, and 145–150 min buffer A was employed.

Eluted peptides were analyzed using a Thermo Scientific Q-Exactive Plus high-resolution quadrupole Orbitrap mass spectrometer, which was directly coupled to the HPLC. Data-dependent acquisition was obtained using Xcalibur version 4.0 software in positive ion mode with a spray voltage of 2.00 kV and a capillary temperature of 275�C and a retention factor of 60. MS1 spectra were measured at a resolution of 70,000, an automatic gain control (AGC) of 3 � 10^6^ with a maximum ion time of 100 ms and a mass range of 400–2,000 *m/z*. Up to 15 MS2 were triggered at a resolution of 17,500 with a fixed first mass of 120 *m/z* for the phosphoproteome and 115 *m/z* for the proteome. An AGC of 1 � 105 with a maximum ion time of 50 ms, an isolation window of 1.3 *m/z* for phosphoproteome and 1.2 *m/z* for proteome, and a normalized collision energy of 31 and 32 were used for nonmodified and phosphoproteomes, respectively. Charge exclusion was set to unassigned, 1, 5–8, and >8. MS1 that triggered MS2 scans were dynamically excluded for 25 or 30 s for nonmodified and phosphoproteomes, respectively.

#### Data analysis

The raw data were analyzed using MaxQuant version 1.6.1.0 (Tyanova et al., 2016). Spectra were searched, using the Andromeda search engine (Cox et al., 2011) against the Arabidopsis TAIR10 proteome file entitled “TAIR10_pep_20101214” that was downloaded from the TAIR website (https://www.arabidopsis.org/download/index-auto.jsp?dir=%2Fdownload_files%2FProteins%2FTAIR10_protein_lists) and was complemented with reverse decoy sequences and common contaminants by MaxQuant. Carbamidomethyl cysteine was set as a fixed modification, while methionine oxidation and protein N-terminal acetylation were set as variable modifications. For the phosphoproteome, “Phosho STY” was also set as a variable modification. The sample type was set to “Reporter Ion MS2” with “6plex” “MT” selected for both lysine and N termini. TMT batch-specific correction factors were configured in the MaxQuant modifications tab (TMT Lot SH254566). Digestion parameters were set to “specific” and “Trypsin/P;LysC.” Up to two missed cleavages were allowed. A false-discovery rate, calculated in MaxQuant using a target-decoy strategy (Elias and Gygi, 2007) of less than 0.01 at both the peptide spectral match and protein identification level was required. The “second peptide” option to identify co-fragmented peptides was not used. The match between runs feature of MaxQuant was not utilized.

Prior to statistical analysis, protein/phosphosite intensity data were normalized such that the total intensity for each TMT lane was equal across the run (referred to as sample loading normalization). No imputation for missing values was performed. Then, statistical analysis was performed using the PoissonSeq package in R (Li et al., 2012). Proteins were categorized as differentially abundant if they had a *q* ≤ 0.05. For phosphosites, we categorized differential abundance as those sites with *q* ≤ 0.2 based on the *q*-value histogram (Supplemental Figure S1).

### RNA extraction and 3′-RNA sequencing

Total RNA was extracted using RNeasy Plant Mini Kit (Qiagen, Hilden, Germany; catalog # 74904), and genomic DNA contamination was removed using RNase-free DNase I Set (Qiagen; catalog # 79254) in-column during RNA extraction according to the manufacturer’s protocols. Libraries were constructed using QuantSeq 3′-mRNA-Seq Library Prep Kit from Illumina. Libraries were run on a HiSeq4000 with 50-bp reads, as QuantSeq is optimized for 50- to 100-bp reads (Moll et al., 2014). Each library was run twice to increase the average read count to ∼6 million reads per sample. Read alignment to the TAIR10 genome was performed using the STAR aligner (Dobin et al., 2013) using default parameters exce– –outFilterMismatchNoverLmax was set to 0.6 to account for the shorter QuantSeq reads, resulting in ∼70% of reads uniquely mapped per sample, which is expected for QuantSeq (Moll et al., 2014). Counts per transcript were obtained using htseq-count using the intersection-nonempty parameter for reads spanning more than one feature (Anders et al., 2015).

### Data analysis of the transcriptome, proteome, and phosphoproteome

The prcomp function in R, and PCA plots were constructed using ggbiplot (https://github.com/vqv/ggbiplot).

GO enrichment analysis and network reconstruction were performed using the ClueGO application in Cytoscape (Bindea et al., 2009). Terms were considered enriched if they had a corrected *P* <0.05.

Volcano plots were constructed with ggplot2 (Wickham, 2016) in R using –Log_10_-transformed *q*-values and Log_2_(Fold-change [*fer*/WT]) of protein.

The enrichment of consensus sequences for FER phosphorylation was performed using the motifeR R package (Wang et al., 2019a, 2019b) with default settings. Sequence logos were made using the ggseqlogo R package (Wagih, 2017). Both analyses were performed in R version 3.6.2 (R Core Team, 2019).

Venn diagrams were generated using Venny (http://bioinfogp.cnb.csic.es/tools/venny/).

### Plasmids construction

Using primers listed in Supplemental Table S1, *ABI5* was PCR amplified from Col-0 cDNA and cloned into vector pXY136-35SP-YFP (Nolan et al., 2017). The *ABI5^S145A^* coding sequence was cloned using two-step PCR. First, two fragments were generated using forward primer 5′-gcaggatccATGGTAACTAGAGAAACGAAG-3′ and reverse primer with the mutation, 5′-ggaagtgtcaaag**C**gccttgtcgaggaagac-3′; forward primer 5′-ctcgacaaggc**G**ctttgacacttccagc-3′ and reverse primer 5′-caggtcgacGAGTGGACAACTCGGGTTC-3′, using the pXY136-35SP-ABI5-YFP vector as template. Second, the full-length *ABI5^S145A^* coding sequence was then PCR amplified using forward primer 5′-gcaggatccATGGTAACTAGAGAAACGAAG-3′ and reverse primer 5′-caggtcgacGAGTGGACAACTCGGGTTC-3′, and cloned into vector pXY136-35SP-YFP.

The sequences encoding the substrate proteins: ABF3, OXS2, ABI5, ABI5^S145A^, SHOU4, and NAIP1 were PCR amplified and cloned into pMBP-H to generate MBP fusion proteins (Guo et al., 2018). The cloning primers are listed in Supplemental Table S1. The coding sequences of *FBH3*, *GT2*, *NSI*, and At35g1950 cloned into pDEST22 were obtained from TAIR (https://www.arabidopsis.org/). The coding sequences were then cloned into pDONR-221 and then pDEST-HIS MBP to generate MBP fusion proteins. All the constructs used in this study were listed in Supplemental Table S2.

### In vitro kinase assay

The in vitro kinase assay was carried out as described (Guo et al., 2018). Briefly, recombinant purified GST-FERK was mixed with MBP-substrate proteins in 20-�L reactions, with 10 �Ci^32^P-γATP and incubated at room temperature for 1 h. The reactions were stopped using 20 �L 2 � SDS sample buffer and resolved on an 8% (w/v) SDS–PAGE.

### BiFC assays

The *mFER* coding sequence was cloned in-frame and upstream of the sequence encoding the N terminus of YFP, while the *SHOU4*, *FBH3*, *ABI5*, and *DDL* coding sequences were cloned in-frame and upstream of the sequence encoding the C terminus of YFP. All resulting plasmids were individually introduced into Agrobacterium (*Agrobacterium tumefaciens*) (strain GV3101). Positive Agrobacterium colonies were cultured in liquid LB medium containing 0.2-mM acetosyringone for 1–2 days. Cells were collected by centrifugation, washed, and resuspended in infiltration buffer (10-mM MgCl_2_, 10-mM MES, pH 5.7, 0.2-mM acetosyringone) to a final optical density (OD) measured at a wavelength of 600 nm (OD_600_) of 0.9. Agrobacteria carrying appropriate pairs of *nYFP* and *cYFP* constructs were mixed in a 1:1 ratio and infiltrated into the lower surface of *N.* *benthamiana* leaves from 2-month-old plants grown on soil. After 36 h, YFP signals were detected using a Zeiss Laser Scanning Microscope 700 (LSM700).

### Transient infiltration assays

Agrobacteria carrying the constructs of interest were grown in liquid LB medium with antibiotics in a 30�C shaker for 2 days. After collecting the agrobacteria by centrifugation, the cells were resuspended in infiltration buffer (10-mM MgCl_2_, 10-mM MES pH 5.7, 200-μM acetosyringone) to a final OD_600_ of 0.3. Leaf infiltration was conducted with a 1-mL syringe on the abaxial side of the leaves, as above. At least two biological replicates were examined for each target construct.

Two days after infiltration, five leaf discs (7 mm in diameter) were collected for each sample and flash-frozen in liquid nitrogen and ground directly in 200 μL of 2 � SDS sample buffer. The samples were resolved on 8% (w/v) SDS–PAGE, followed by immunoblotting using commercial rabbit anti-GFP (A11122; Invitrogen, Waltham, MA, USA) or anti-FLAG antibodies (F7425; Sigma-Aldrich, St Louis, MO, USA) at a 1:1,000 dilution.

### Co-IP

Agrobacteria carrying constructs encoding FLAG- and GFP-tagged proteins of interest were co-infiltrated into *N. benthamiana* leaves. Leaf samples were collected 2 days after the infiltration. One gram of each sample was ground in liquid nitrogen and homogenized in 2.5 mL IP buffer (10-mM HEPES Ph 7.5, 100-mM NaCl, 1-mM EDTA, 10% [v/v] glycerol, and 0.5% [v/v] Nonidet P-40) with 1-mM PMSF, 20-�M MG132, and proteinase inhibitor cocktail for 20 min at 4�C with rotation. Five micrograms of FLAG M2 antibody (F1804; Sigma-Aldrich) was prebound to 60-�L protein G Dynabeads (10003D; Thermo Fisher Scientific) for 30 min in 1� phosphate buffer saline (PBS) buffer with 0.02% (v/v) Tween-20 at room temperature. The beads were washed once with the same PBS buffer and resuspended in Co-IP buffer. After protein extraction, 20 �L of anti-FLAG prebound Dynabeads was added to each sample for another 1.5 h incubation at 4�C with rotation. Dynabeads were precipitated using DynaMagnetic rack (12321D; Thermo Fisher Scientific) and washed twice with Co-IP buffer with 0.5% (v/v) Nonidet P-40 and twice with Co-IP buffer without Nonidet P-40. The IP product was resuspended in 2� SDS sample buffer and used for immunoblotting with rabbit anti-GFP (A11122; Invitrogen) and rabbit anti-FLAG antibody (F7425; Sigma-Aldrich).

### ER body observation and quantification

For ER body observation, GFP-HDEL-labeled structures were observed and images were taken by confocal microscopy using a Zeiss Laser Scanning Microscope 700 (LSM700) with a 63� oil immersion objective. GFP was excited with a 488 nm laser line and detected at 555 nm. The ER body quantification was carried out as described (Yamada et al., 2008).

### Measurement of glucosinolate contents

Glucosinolates were measured as previously described (Brown et al., 2003; Kliebenstein et al., 2001a–c). Briefly, 3-week-old whole rosettes were pooled, weighed, frozen, and harvested in 90% (v/v) methanol. Tissues were homogenized for 3 min in a paint shaker, centrifuged, and the supernatants were transferred to a 96-well filter plate with DEAE Sephadex. The filter plate with DEAE Sephadex was washed once with water, once with 90% (v/v) methanol, and once with water again. The Sephadex-bound glucosinolates were eluted after an overnight incubation with 110 μL of sulfatase. Individual desulfo-glucosinolates within each sample were separated and detected by high-performance liquid chromatography (HPLC)-diode array detection, identified using retention time and absorbance spectra developed from purified compounds and re-validated using Arabidopsis genotypes with known chemotypes, quantified using relative response factors developed by comparison to standard curves from purified compounds as previously reported (Brown et al., 2003), and further normalized to fresh weight.

### Cotyledon greening assay

Surface-sterilized seeds were germinated on control half-strength MS medium or half-strength MS medium containing 1-�M ABA under constant light. Cotyledon greening was observed 4–5 days later. The cotyledon greening rate was calculated using the percentage of seeds with green cotyledons out of all seeds sown, for each genotype.

### CHX and ABA treatments for protein stability assays

For ABI5 protein stability assays, 3-week-old rosette leaves were collected and cut into smaller pieces and incubated in liquid half-strength MS medium for 2 h before adding 10-�M ABA. Samples were then collected at the indicated times. Time 0 was collected right before the addition of ABA. Total proteins were extracted using 2� SDS buffer and samples were immunoblotted with rabbit anti-GFP antibody (A11122; Invitrogen).

For ABI ^S145A^-YFP protein stability assays, 7-day-old seedlings of the *ABI5-YFP* and *ABI ^S145A^-YFP* transgenic lines were treated with 0.5-mM CHX in half-strength liquid MS medium for the indicated times. Total proteins were extracted in 2� SDS loading buffer and samples were immunoblotted with rabbit anti-GFP antibody (A11122; Invitrogen).

For endogenous FER protein detection, samples were immunoblotted with lab-made rabbit anti-FER antibody.

### RT-qPCR

Total RNA was isolated from 3-week-old rosette leaves using an RNeasy Plant Mini Kit (Qiagen; catalog # 74904) and genomic DNA was removed using RNase-free DNase Set (Qiagen; catalog # 79254) in-column during RNA extraction. The first-strand cDNA was synthesized with an iScript cDNA Synthesis Kit (BioRad, Hercules, CA, USA; catalog # 1708891). Real-time PCR was performed using SYBR Green PCR Master Mix (Applied Biosystems, Waltham, MA, USA; catalog # 4309155) on the StepOnePlus Real-Time PCR system (Applied Biosystems). Relative gene expression was determined by applying the 2^–ΔΔCT^ (CT, cycle threshold) method and normalized to the expression of the reference gene *ACTIN2* (At3g18780). RT-qPCR was performed with two to three technical replicates from three to four independent biological replicates. Primers used for qPCR are provided in Supplemental Table S1.

### Statistical analysis

Graphs were created in GraphPad Prism software (version 9.3.0). SPSS version 27.0 software (IBM, Armonk, NY, USA) was used for statistical data analysis. The data are shown as means � standard error of the mean (sem) and were subjected to one-way analysis of variance (ANOVA) Tukey’s multiple range tests (*P* < 0.05). ANOVA data are provided in Supplemental Data Set 7.

### Accession numbers

The accession numbers of genes discussed in this article are: *FER* (At3g51550), *SHOU4* (At1g78880), *FBH3* (At1g51140), *ABI5* (At2G36270), *NAI1* (At2g22770) and *NAI2* (At3g15950)*, PYK10* (At3g09260)*, NAIP1* (At4G15545)*, ABF3* (At4g34000)*, OXS2* (At2g41900), At3g51950*, GT2* (At1g76890)*, NSI* (At1G32070)*, ASA1* (At5g05730)*, IGMT5* (At1g76790)*, MYC2* (At1g32640)*, CYP83B1* (At4g31500)*, CYP79B2* (At4g39950)*, CYP79B3* (At2g22330), and *DDL* (At3g20550)*.* Raw sequencing data have been deposited at the Gene Expression Omnibus under series GSE143634 and GSE191303. Raw proteomics data have been deposited on MassIVE (https://massive.ucsd.edu) under series MSV000084804.

## Supplemental data 

The following materials are available in the online version of this article.

**Supplemental Figure S1.** FER omics data analyses parameters.

**Supplemental Figure S2.** The comparisons of differentially expressed transcripts and proteins in *fer*, and the differentially expressed transcripts of this study to that of the previous publication.

**Supplemental Figure S3.** Enriched GO terms in transcripts with increased levels in *fer*.

**Supplemental Figure S4.** Enriched GO terms in transcripts with decreased levels in *fer*.

**Supplemental Figure S5.** Enriched GO terms in proteins with increased levels in *fer*.

**Supplemental Figure S6.** Enriched GO terms in proteins with decreased levels in *fer*.

**Supplemental Figure S7.** Enriched GO terms in phosphoproteins with increased levels in *fer*.

**Supplemental Figure S8.** Enriched GO terms in phosphoproteins with decreased levels in *fer*.

**Supplemental Figure S9.** FER directly phosphorylates many proteins with diverse functions.

**Supplemental Figure S10.** FER negatively regulates ER-body formation.

**Supplemental Figure S11.** FER negative regulation of ER-body formation is validated using *FER-*miRNA knockdown.

**Supplemental Figure S12.** FER negatively regulates ABA response during cotyledon greening through ABI5.

**Supplemental Figure S13.** FER regulates >20% of TFs in the Arabidopsis genome, directly or indirectly.

**Supplemental Table S1.** Primers used in this study.

**Supplemental Table S2.** Constructs used in this study.

**Supplemental Data Set 1.** Misregulated gene list in FER omics data.

**Supplemental Data Set 2.** Enriched GO terms in FER omics data.

**Supplemental Data Set 3.** Enriched consensus sequences of FER phosphorylation sites.

**Supplemental Data Set 4.** Misregulated genes in 10-day-old *fer* seedlings.

**Supplemental Data Set 5.** The comparisons of FER omics data with ABF1, ABF3, ABF4, and FBH3 target genes (Song et al., 2016).

**Supplemental Data Set 6.** Transcription factors that are differentially expressed in FER omics data.

**Supplemental Data Set 7.** ANOVA results in this study.

## Supplementary Material

koac111_Supplementary_Data

## Notes

H.G., Y.Y., and J.W.W. conceived the research. P.W. performed genetic, molecular, biochemical, and cell biology analysis. G.S. performed proteomics and phosphoproteomics. N.M.C., G.S., and T.M.N. performed the omics data analysis. N.M.C. performed statistical analysis for transcript and protein enrichment. P.M.B. contributed to biochemical experiments. J.K.P. and J.J.K. contributed to SHOU4 analysis. D.C.B. and C.L. contributed to data analysis. E.K. and D.J.K. performed glucosinolate measurements. C.M.S. performed the motifeR to identify consensus sequence for FER phosphosites. H.G. and P.W. wrote the manuscript, with edits or input from other co-authors.

The authors responsible for distribution of materials integral to the findings presented in this article in accordance with the policy described in the Instructions for Authors (https://academic.oup.com/plcell) are Hongqing Guo (hguo@iastate.edu) and Yanhai Yin (yin@iastate.edu).

## References

[koac111-B1] Anders S , Pyl PT, Huber W (2015) HTSeq–a Python framework to work with high-throughput sequencing data. Bioinformatics 31: 166–169 25260700 10.1093/bioinformatics/btu638 PMC4287950

[koac111-B2] Batoko H , Zheng HQ, Hawes C, Moore I (2000) A rab1 GTPase is required for transport between the endoplasmic reticulum and golgi apparatus and for normal golgi movement in plants. Plant Cell 12: 2201–2218 11090219 10.1105/tpc.12.11.2201 PMC150168

[koac111-B3] Bindea G , Mlecnik B, Hackl H, Charoentong P, Tosolini M, Kirilovsky A, Fridman WH, Pages F, Trajanoski Z, Galon J (2009) ClueGO: a Cytoscape plug-in to decipher functionally grouped gene ontology and pathway annotation networks. Bioinformatics 25: 1091–1093 19237447 10.1093/bioinformatics/btp101 PMC2666812

[koac111-B4] Boisson-Dernier A , Franck CM, Lituiev DS, Grossniklaus U (2015) Receptor-like cytoplasmic kinase MARIS functions downstream of CrRLK1L-dependent signaling during tip growth. Proc Natl Acad Sci USA 112: 12211–12216 26378127 10.1073/pnas.1512375112 PMC4593096

[koac111-B5] Brown PD , Tokuhisa JG, Reichelt M, Gershenzon J (2003) Variation of glucosinolate accumulation among different organs and developmental stages of Arabidopsis thaliana. Phytochemistry 62: 471–481 12620360 10.1016/s0031-9422(02)00549-6

[koac111-B6] Burrow M , Halkier BA, Kliebenstein DJ (2010) Regulatory networks of glucosinolates shape Arabidopsis thaliana fitness. Curr Opin Plant Biol 13: 347–352 10.1016/j.pbi.2010.02.002 20226722

[koac111-B7] Chakravorty D , Yu Y, Assmann SM (2018) A kinase-dead version of FERONIA receptor-like kinase has dose-dependent impacts on rosette morphology and RALF1-mediated stomatal movements. FEBS Lett 592: 3429–3437 30207378 10.1002/1873-3468.13249 PMC6205910

[koac111-B8] Chen J , Yu F, Liu Y, Du C, Li X, Zhu S, Wang X, Lan W, Rodriguez PL, Liu X, et al (2016) FERONIA interacts with ABI2-type phosphatases to facilitate signaling cross-talk between abscisic acid and RALF peptide in Arabidopsis. Proc Natl Acad Sci USA 113: E5519–5527 27566404 10.1073/pnas.1608449113 PMC5027425

[koac111-B9] Cox J , Neuhauser N, Michalski A, Scheltema RA, Olsen JV, Mann M (2011) Andromeda: a peptide search engine integrated into the MaxQuant environment. J Proteome Res 10: 1794–1805 21254760 10.1021/pr101065j

[koac111-B10] Deslauriers SD , Larsen PB (2010) FERONIA is a key modulator of brassinosteroid and ethylene responsiveness in Arabidopsis hypocotyls. Mol Plant 3: 626–640 20400488 10.1093/mp/ssq015

[koac111-B11] Dobin A , Davis CA, Schlesinger F, Drenkow J, Zaleski C, Jha S, Batut P, Chaisson M, Gingeras TR (2013) STAR: ultrafast universal RNA-seq aligner. Bioinformatics 29: 15–21 23104886 10.1093/bioinformatics/bts635 PMC3530905

[koac111-B12] Du C , Li X, Chen J, Chen W, Li B, Li C, Wang L, Li J, Zhao X, Lin J, et al (2016) Receptor kinase complex transmits RALF peptide signal to inhibit root growth in Arabidopsis. Proc Natl Acad Sci USA 113: E8326–E8334 27930296 10.1073/pnas.1609626113 PMC5187724

[koac111-B13] Duan Q , Kita D, Li C, Cheung AY, Wu HM (2010) FERONIA receptor-like kinase regulates RHO GTPase signaling of root hair development. Proc Natl Acad Sci USA 107: 17821–17826 20876100 10.1073/pnas.1005366107 PMC2955125

[koac111-B14] Duan Q , Kita D, Johnson EA, Aggarwal M, Gates L, Wu HM, Cheung AY (2014) Reactive oxygen species mediate pollen tube rupture to release sperm for fertilization in Arabidopsis. Nat Commun 5: 3129 24451849 10.1038/ncomms4129

[koac111-B15] Duan Q , Liu MJ, Kita D, Jordan SS, Yeh FJ, Yvon R, Carpenter H, Federico AN, Garcia-Valencia LE, Eyles SJ, et al (2020) FERONIA controls pectin- and nitric oxide-mediated male-female interaction. Nature 579: 561–566 32214247 10.1038/s41586-020-2106-2

[koac111-B16] Dunser K , Gupta S, Herger A, Feraru MI, Ringli C, Kleine-Vehn J (2019) Extracellular matrix sensing by FERONIA and Leucine-Rich Repeat Extensins controls vacuolar expansion during cellular elongation in Arabidopsis thaliana. EMBO J 35: e100353 10.15252/embj.2018100353 PMC6443208 30850388

[koac111-B17] Elias JE , Gygi SP (2007) Target-decoy search strategy for increased confidence in large-scale protein identifications by mass spectrometry. Nat Methods 4: 207–214 17327847 10.1038/nmeth1019

[koac111-B18] Escobar-Restrepo JM , Huck N, Kessler S, Gagliardini V, Gheyselinck J, Yang WC, Grossniklaus U (2007) The FERONIA receptor-like kinase mediates male-female interactions during pollen tube reception. Science 317: 656–660 17673660 10.1126/science.1143562

[koac111-B19] Feng W , Kita D, Peaucelle A, Cartwright HN, Doan V, Duan Q, Liu MC, Maman J, Steinhorst L, Schmitz-Thom I, et al (2018) The FERONIA receptor kinase maintains cell-wall integrity during aalt stress through Ca(2+) signaling. Curr Biol 28: 666–675 e665 29456142 10.1016/j.cub.2018.01.023 PMC5894116

[koac111-B20] Franck CM , Westermann J, Boisson-Dernier A (2018) Plant malectin-like receptor kinases: from cell wall integrity to immunity and beyond. Annu Rev Plant Biol 69: 301–328 29539271 10.1146/annurev-arplant-042817-040557

[koac111-B21] Geem KR , Kim DH, Lee DW, Kwon Y, Lee J, Kim JH, Hwang I (2019) Jasmonic acid-inducible TSA1 facilitates ER body formation. Plant J 97: 267–280 30267434 10.1111/tpj.14112

[koac111-B22] Guan C , Wang X, Feng J, Hong S, Liang Y, Ren B, Zuo J (2014) Cytokinin antagonizes abscisic acid-mediated inhibition of cotyledon greening by promoting the degradation of abscisic acid insensitive5 protein in Arabidopsis. Plant Physiol 164: 1515–1526 24443524 10.1104/pp.113.234740 PMC3938637

[koac111-B23] Guo H , Ye H, Li L, Yin Y (2009a) A family of receptor-like kinases are regulated by BES1 and involved in plant growth in *Arabidopsis thaliana*. Plant Signal Behav 4: 784–786 19820315 10.4161/psb.4.8.9231 PMC2801400

[koac111-B24] Guo H , Li L, Ye H, Yu X, Algreen A, Yin Y (2009b) Three related receptor-like kinases are required for optimal cell elongation in *Arabidopsis thaliana*. Proc Natl Acad Sci USA 106: 7648–7653 19383785 10.1073/pnas.0812346106 PMC2678668

[koac111-B25] Guo H , Nolan TM, Song G, Liu S, Xie Z, Chen J, Schnable PS, Walley JW, Yin Y (2018) FERONIA receptor kinase contributes to plant immunity by suppressing jasmonic acid signaling in Arabidopsis thaliana. Curr Biol 28: 3316–3324 e3316 30270181 10.1016/j.cub.2018.07.078

[koac111-B26] Hara-Nishimura I , Matsushima R (2003) A wound-inducible organelle derived from endoplasmic reticulum: a plant strategy against environmental stresses? Curr Opin Plant Biol 6: 583–588 14611957 10.1016/j.pbi.2003.09.015

[koac111-B27] Haruta M , Sabat G, Stecker K, Minkoff BB, Sussman MR (2014) A peptide hormone and its receptor protein kinase regulate plant cell expansion. Science 343: 408–411 24458638 10.1126/science.1244454 PMC4672726

[koac111-B28] Haruta M , Gaddameedi V, Burch H, Fernandez D, Sussman MR (2018) Comparison of the effects of a kinase-dead mutation of FERONIA on ovule fertilization and root growth of Arabidopsis. FEBS Lett 592: 2395–2402 29904923 10.1002/1873-3468.13157

[koac111-B29] Hayashi Y , Yamada K, Shimada T, Matsushima R, Nishizawa NK, Nishimura M, Hara-Nishimura I (2001) A proteinase-storing body that prepares for cell death or stresses in the epidermal cells of Arabidopsis. Plant Cell Physiol 42: 894–899 11577182 10.1093/pcp/pce144

[koac111-B30] Herger A , Dunser K, Kleine-Vehn J, Ringli C (2019) Leucine-rich repeat extensin proteins and their role in cell wall sensing. Curr Biol 29: R851–R858 31505187 10.1016/j.cub.2019.07.039

[koac111-B31] Hou Y , Guo X, Cyprys P, Zhang Y, Bleckmann A, Cai L, Huang Q, Luo Y, Gu H, Dresselhaus T, et al (2016) Maternal ENODLs are required for pollen tube reception in Arabidopsis. Curr Biol 26: 2343–2350 27524487 10.1016/j.cub.2016.06.053 PMC5522746

[koac111-B32] Kessler SA , Lindner H, Jones DS, Grossniklaus U (2015) Functional analysis of related CrRLK1L receptor-like kinases in pollen tube reception. EMBO Rep 16: 107–115 25490905 10.15252/embr.201438801 PMC4304734

[koac111-B33] Kessler SA , Shimosato-Asano H, Keinath NF, Wuest SE, Ingram G, Panstruga R, Grossniklaus U (2010) Conserved molecular components for pollen tube reception and fungal invasion. Science 330: 968–971 21071669 10.1126/science.1195211

[koac111-B34] Kettenbach AN , Gerber SA (2011) Rapid and reproducible single-stage phosphopeptide enrichment of complex peptide mixtures: application to general and phosphotyrosine-specific phosphoproteomics experiments. Anal Chem 83: 7635–7644 21899308 10.1021/ac201894j PMC3251014

[koac111-B152] Kliebenstein DJ , Gershenzon J, Mitchell-Olds T (2001a) Comparative quantitative trait loci mapping of aliphatic, indolic and benzylic glucosinolate production in Arabidopsis thaliana leaves and seeds. Genetics 159: 359–370 11560911 10.1093/genetics/159.1.359 PMC1461795

[koac111-B153] Kliebenstein DJ , Kroymann J, Brown PFiguth A, Pedersen D, Gershenzon J, Mitchell-Olds T (2001b) Genetic control of natural variation in Arabidopsis glucosinolate accumulation. Plant Physiol 126: 811–825 11402209 10.1104/pp.126.2.811 PMC111171

[koac111-B154] Kliebenstein DJ , Lambrix VM, Reichelt MGershenzon J, Mitchell-Olds T (2001c) Gene duplication in the diversification of secondary metabolism: tandem 2-oxoglutarate-dependent dioxygenases control glucosinolate biosynthesis in Arabidopsis. Plant Cell 13: 681–693 11251105 10.1105/tpc.13.3.681 PMC135509

[koac111-B35] Kliebenstein DJ , Figuth A, Mitchell-Olds T (2002) Genetic architecture of plastic methyl jasmonate responses in Arabidopsis thaliana. Genetics 161: 1685–1696 12196411 10.1093/genetics/161.4.1685 PMC1462221

[koac111-B36] Li C , Liu X, Qiang X, Li X, Li X, Zhu S, Wang L, Wang Y, Liao H, Luan S, et al (2018) EBP1 nuclear accumulation negatively feeds back on FERONIA-mediated RALF1 signaling. PLoS Biol 16: e2006340 30339663 10.1371/journal.pbio.2006340 PMC6195255

[koac111-B37] Li C , Yeh FL, Cheung AY, Duan Q, Kita D, Liu MC, Maman J, Luu EJ, Wu BW, Gates L, et al (2015) Glycosylphosphatidylinositol-anchored proteins as chaperones and co-receptors for FERONIA receptor kinase signaling in Arabidopsis. Elife 4: e06587 26052747 10.7554/eLife.06587 PMC4458842

[koac111-B38] Li J , Witten DM, Johnstone IM, Tibshirani R (2012) Normalization, testing, and false discovery rate estimation for RNA-sequencing data. Biostatistics 13: 523–538 22003245 10.1093/biostatistics/kxr031 PMC3372940

[koac111-B39] Lin W , Tang W, Anderson CT, Yang Z (2018) FERONIA’s sensing of cell wall pectin activates ROP GTPase signaling in Arabidopsis. bio Rxiv 10.1101/269647

[koac111-B40] Lin W , Tang W, Pan X, Huang A, Gao X, Anderson CT, Yang Z (2021) Arabidopsis pavement cell morphogenesis requires FERONIA binding to pectin for activation of ROP GTPase signaling. Curr Biol 32: 497–507.E4 10.1016/j.cub.2021.11.030 34875229

[koac111-B41] Liu C , Shen L, Xiao Y, Vyshedsky D, Peng C, Sun X, Liu Z, Cheng L, Zhang H, Chai J, et al (2021) Pollen PCP-B peptides unlock a stigma peptide–receptor kinase gating mechanism for pollination. Science 372: 171–175 33833120 10.1126/science.abc6107

[koac111-B42] Lopez-Molina L , Mongrand S, McLachlin DT, Chait BT, Chua NH (2002) ABI5 acts downstream of ABI3 to execute an ABA-dependent growth arrest during germination. Plant J 32: 317–328 12410810 10.1046/j.1365-313x.2002.01430.x

[koac111-B43] Mang H , Feng B, Hu Z, Boisson-Dernier A, Franck CM, Meng X, Huang Y, Zhou J, Xu G, Wang T, et al (2017) Differential regulation of two-tiered plant immunity and sexual reproduction by ANXUR receptor-like kinases. Plant Cell 29: 3140–3156 29150546 10.1105/tpc.17.00464 PMC5757273

[koac111-B44] Masachis S , Segorbe D, Turra D, Leon-Ruiz M, Furst U, El Ghalid M, Leonard G, Lopez-Berges MS, Richards TA, Felix G, et al (2016) A fungal pathogen secretes plant alkalinizing peptides to increase infection. Nat Microbiol 1: 16043 27572834 10.1038/nmicrobiol.2016.43

[koac111-B45] Matsushima R , Fukao Y, Nishimura M, Hara-Nishimura I (2004) NAI1 gene encodes a basic-helix-loop-helix-type putative transcription factor that regulates the formation of an endoplasmic reticulum-derived structure, the ER body. Plant Cell 16: 1536–1549 15155889 10.1105/tpc.021154 PMC490044

[koac111-B46] Matsushima R , Kondo M, Nishimura M, Hara-Nishimura I (2003a) A novel ER-derived compartment, the ER body, selectively accumulates a beta-glucosidase with an ER-retention signal in Arabidopsis. Plant J 33: 493–502 12581307 10.1046/j.1365-313x.2003.01636.x

[koac111-B47] Matsushima R , Hayashi Y, Yamada K, Shimada T, Nishimura M, Hara-Nishimura I (2003b) The ER body, a novel endoplasmic reticulum-derived structure in Arabidopsis. Plant Cell Physiol 44: 661–666 12881493 10.1093/pcp/pcg089

[koac111-B48] Moll P , Ante M, Seitz A, Reda T (2014) QuantSeq 3’ mRNA sequencing for RNA quantification. Nat Methods 11: i–iii

[koac111-B49] Nakano RT , Pislewska-Bednarek M, Yamada K, Edger PP, Miyahara M, Kondo M, Bottcher C, Mori M, Nishimura M, Schulze-Lefert P, et al (2017) PYK10 myrosinase reveals a functional coordination between endoplasmic reticulum bodies and glucosinolates in Arabidopsis thaliana. Plant J 89: 204–220 27612205 10.1111/tpj.13377

[koac111-B50] Nakazaki A , Yamada K, Kunieda T, Sugiyama R, Hirai MY, Tamura K, Hara-Nishimura I, Shimada T (2019) Leaf ER bodies identified in Arabidopsis rosette leaves are involved in defense against herbivory. Plant Physiol 179: 1515–1524 30696747 10.1104/pp.18.00984 PMC6446793

[koac111-B51] Nambara E , Suzuki M, Abrams S, McCarty DR, Kamiya Y, McCourt P (2002) A screen for genes that function in abscisic acid signaling in Arabidopsis thaliana. Genetics 161: 1247–1255 12136027 10.1093/genetics/161.3.1247 PMC1462180

[koac111-B52] Ngo QA , Vogler H, Lituiev DS, Nestorova A, Grossniklaus U (2014) A calcium dialog mediated by the FERONIA signal transduction pathway controls plant sperm delivery. Dev Cell 29: 491–500 24814317 10.1016/j.devcel.2014.04.008

[koac111-B53] Nolan TM , Brennan B, Yang M, Chen J, Zhang M, Li Z, Wang X, Bassham DC, Walley J, Yin Y (2017) Selective autophagy of BES1 mediated by DSK2 balances plant growth and survival. Dev Cell 41: 33–46 e37 28399398 10.1016/j.devcel.2017.03.013 PMC5720862

[koac111-B54] Ortiz-Morea FA , Liu J, Shan L, He P (2021) Malectin-like receptor kinases as protector deities in plant immunity. Nat Plants10.1038/s41477-021-01028-3. PMC9059209 34931075

[koac111-B55] Polko JK , Barnes WJ, Voiniciuc C, Doctor S, Steinwand B, Hill JL, Tien M, Pauly M, Anderson CT, Kieber JJ (2018) SHOU4 proteins regulate trafficking of cellulose synthase complexes to the plasma membrane. Curr Biol 28: 3174–3182 30245104 10.1016/j.cub.2018.07.076

[koac111-B56] Pruneda-Paz JL , Breton G, Nagel DH, Kang SE, Bonaldi K, Doherty CJ, Ravelo S, Galli M, Ecker JR, Kay SA (2014) A genome-scale resource for the functional characterization of Arabidopsis transcription factors. Cell Rep 8: 622–632 25043187 10.1016/j.celrep.2014.06.033 PMC4125603

[koac111-B57] Sarkar SNS , Kunieda T, Hara-Nishimura I, Yamada K (2021) The Arabidopsis transcription factor NAI1 activates the NAI2 promoter by binding to the G-box motifs. Plant Signal Behav 16: 1846928 33315514 10.1080/15592324.2020.1846928 PMC7849731

[koac111-B58] Sherameti I , Venus Y, Drzewiecki C, Tripathi S, Dan VM, Nitz I, Varma A, Grundler FM, Oelmueller R (2008) PYK10, a beta-glucosidase located in the endoplasmatic reticulum, is crucial for the beneficial interaction between Arabidopsis thaliana and the endophytic fungus Piriformospora indica. Plant J 54: 428–439 18248598 10.1111/j.1365-313X.2008.03424.x

[koac111-B59] Shih HW , Miller ND, Dai C, Spalding EP, Monshausen GB (2014) The receptor-like kinase FERONIA is required for mechanical signal transduction in Arabidopsis seedlings. Curr Biol 24: 1887–1892 25127214 10.1016/j.cub.2014.06.064

[koac111-B60] S�nderby IE , Geu-Flores F, Halkier BA (2010) Biosynthesis of glucosinolates–gene discovery and beyond. Trends Plant Sci 15: 283–290 20303821 10.1016/j.tplants.2010.02.005

[koac111-B61] Song G , Brachova L, Nikolau BJ, Jones AM, Walley JW (2018b) Heterotrimeric G-protein-dependent proteome and phosphoproteome in unstimulated Arabidopsis roots. Proteomics 18: e1800323 30407730 10.1002/pmic.201800323 PMC6298806

[koac111-B62] Song G , Hsu PY, Walley JW (2018a) Assessment and refinement of sample preparation methods for deep and quantitative plant proteome profiling. Proteomics 18: e1800220 30035338 10.1002/pmic.201800220 PMC6296749

[koac111-B63] Song G , Montes C, Walley JW (2020) Quantitative profiling of protein abundance and phosphorylation state in plant tissues using tandem mass tags. Methods Mol Biol 2139: 147–156 32462584 10.1007/978-1-0716-0528-8_11

[koac111-B64] Song L , Huang SC, Wise A, Castanon R, Nery JR, Chen H, Watanabe M, Thomas J, Bar-Joseph Z, Ecker JR (2016) A transcription factor hierarchy defines an environmental stress response network. Science 354: aag1550 27811239 10.1126/science.aag1550 PMC5217750

[koac111-B65] Song Y , Wilson AJ, Zhang XC, Thoms D, Sohrabi R, Song S, Geissmann Q, Liu Y, Walgren L, He SY, et al (2021) FERONIA restricts Pseudomonas in the rhizosphere microbiome via regulation of reactive oxygen species. Nat Plants 7: 644–654 33972713 10.1038/s41477-021-00914-0

[koac111-B66] Stefanik N , Bizan J, Wilkens A, Tarnawska-Glatt K, Goto-Yamada S, Strzałka K, Nishimura M, Hara-Nishimura I, Yamada K (2020) NAI2 and TSA1 drive differentiation of constitutive and inducible ER body formation in brassicaceae. Plant Cell Physiol 61: 722–734 31879762 10.1093/pcp/pcz236

[koac111-B67] Stegmann M , Monaghan J, Smakowska-Luzan E, Rovenich H, Lehner A, Holton N, Belkhadir Y, Zipfel C (2017) The receptor kinase FER is a RALF-regulated scaffold controlling plant immune signaling. Science 355: 287–289 28104890 10.1126/science.aal2541

[koac111-B68] Tang W , Lin W, Zhou X, Guo J, Dang X, Li B, Lin D, Yang Z (2021) Mechano-transduction via the pectin-FERONIA complex activates ROP6 GTPase signaling in Arabidopsis pavement cell morphogenesis. Curr Biol 32: 508–517.E3 10.1016/j.cub.2021.11.031. 34875231

[koac111-B151] Tyanova S , Temu T, Cox J (2016) The MaxQuant computational platform for mass spectrometry-based shotgun proteomics. Nat Protoc 11: 2301–2319 27809316 10.1038/nprot.2016.136

[koac111-B69] Wagih O (2017) ggseqlogo: a versatile R package for drawing sequence logos. Bioinformatics 33: 3645–3647 29036507 10.1093/bioinformatics/btx469

[koac111-B70] Walley JW , Shen Z, McReynolds MR, Schmelz EA, Briggs SP (2018) Fungal-induced protein hyperacetylation in maize identified by acetylome profiling. Proc Natl Acad Sci USA 115: 210–215 29259121 10.1073/pnas.1717519115 PMC5776827

[koac111-B71] Wang JZ , Li B, Xiao Y, Ni Y, Ke H, Yang P, de Souza A, Bjornson M, He X, Shen Z, et al (2017) Initiation of ER body formation and indole glucosinolate metabolism by the plastidial retrograde signaling metabolite, MEcPP. Mol Plant 10: 1400–1416 28965830 10.1016/j.molp.2017.09.012 PMC6368977

[koac111-B72] Wang S , Cai Y, Cheng J, Li W, Liu Y, Yang H (2019a) motifeR: an integrated web software for identification and visualization of protein posttranslational modification motifs. Proteomics 19: e1900245 31622013 10.1002/pmic.201900245

[koac111-B73] Wang Z , Li X, Liu N, Peng Q, Wang Y, Fan B, Zhu C, Chen Z (2019b) A family of NAI2-interacting proteins in the biogenesis of the ER body and rlated structures. Plant Physiol 180: 212–227 30770459 10.1104/pp.18.01500 PMC6501091

[koac111-B74] Wickham H (2016) ggplot2: Elegant Graphics for Data Analysis. Springer-Verlag, New York, NY

[koac111-B75] Xiao Y , Stegmann M, Han Z, DeFalco TA, Parys K, Xu L, Belkhadir Y, Zipfel C, Chai J (2019) Mechanisms of RALF peptide perception by a heterotypic receptor complex. Nature 572: 270–274 31291642 10.1038/s41586-019-1409-7

[koac111-B76] Yamada K , Goto-Yamada S, Nakazaki A, Kunieda T, Kuwata K, Nagano AJ, Nishimura M, Hara-Nishimura I (2020) single-cell chemical defense in Brassicaceae plants. Commun Biol 3: 1–10 31937912 10.1038/s42003-019-0739-1 PMC6959254

[koac111-B77] Yamada K , Nagano AJ, Nishina M, Hara-Nishimura I, Nishimura M (2008) NAI2 is an endoplasmic reticulum body component that enables ER body formation in Arabidopsis thaliana. Plant Cell 20: 2529–2540 18780803 10.1105/tpc.108.059345 PMC2570739

[koac111-B78] Yu Y , Chakravorty D, Assmann SM (2018) The G protein beta-subunit, AGB1, interacts with FERONIA in RALF1-regulated stomatal movement. Plant Physiol 176: 2426–2440 29301953 10.1104/pp.17.01277 PMC5841690

[koac111-B79] Yu F , Qian L, Nibau C, Duan Q, Kita D, Levasseur K, Li X, Lu C, Li H, Hou C, et al (2012) FERONIA receptor kinase pathway suppresses abscisic acid signaling in Arabidopsis by activating ABI2 phosphatase. Proc Natl Acad Sci USA 109: 14693–14698 22908257 10.1073/pnas.1212547109 PMC3437822

[koac111-B80] Yu F , Wu Y, Xie D (2015) Precise protein post-translational modifications modulate ABI5 activity. Trends Plant Sci 20: 569–575 26044742 10.1016/j.tplants.2015.05.004

[koac111-B81] Zhang X , Peng H, Zhu S, Xing J, Li X, Zhu Z, Zheng J, Wang L, Wang B, Chen J, et al (2020) Nematode-encoded RALF peptide mimics facilitate parasitism of plants through the FERONIA receptor kinase. Mol Plant 13: 1434–1454 32896643 10.1016/j.molp.2020.08.014

[koac111-B82] Zhao C , Zayed O, Yu Z, Jiang W, Zhu P, Hsu CC, Zhang L, Tao WA, Lozano-Duran R, Zhu JK (2018) Leucine-rich repeat extensin proteins regulate plant salt tolerance in Arabidopsis. Proc Natl Acad Sci USA 115: 13123–13128 30514814 10.1073/pnas.1816991115 PMC6305001

[koac111-B83] Zhu S , Estevez JM, Liao H, Zhu Y, Yang T, Li C, Wang Y, Li L, Liu X, Pacheco JM, et al (2020) The RALF1-FERONIA complex phosphorylates eIF4E1 to promote protein synthesis and polar root hair growth. Mol Plant 13: 698–716 31904511 10.1016/j.molp.2019.12.014

