# Supplementary material for: Integrated omics reveal novel functions and underlying mechanisms of the receptor kinase FERONIA in Arabidopsis thaliana
Source: Plant Cell. 2022 Apr 18;34(7):2594–614. doi: 10.1093/plcell/koac111 (PMC9252503; doi:10.1093/plcell/koac111)
Supplement: koac111_Supplementary_Data [file koac111_supplementary_data.zip › koac111-suppl_data/TPC2021LSB00634R2 Supp Figures and Tables.pdf]

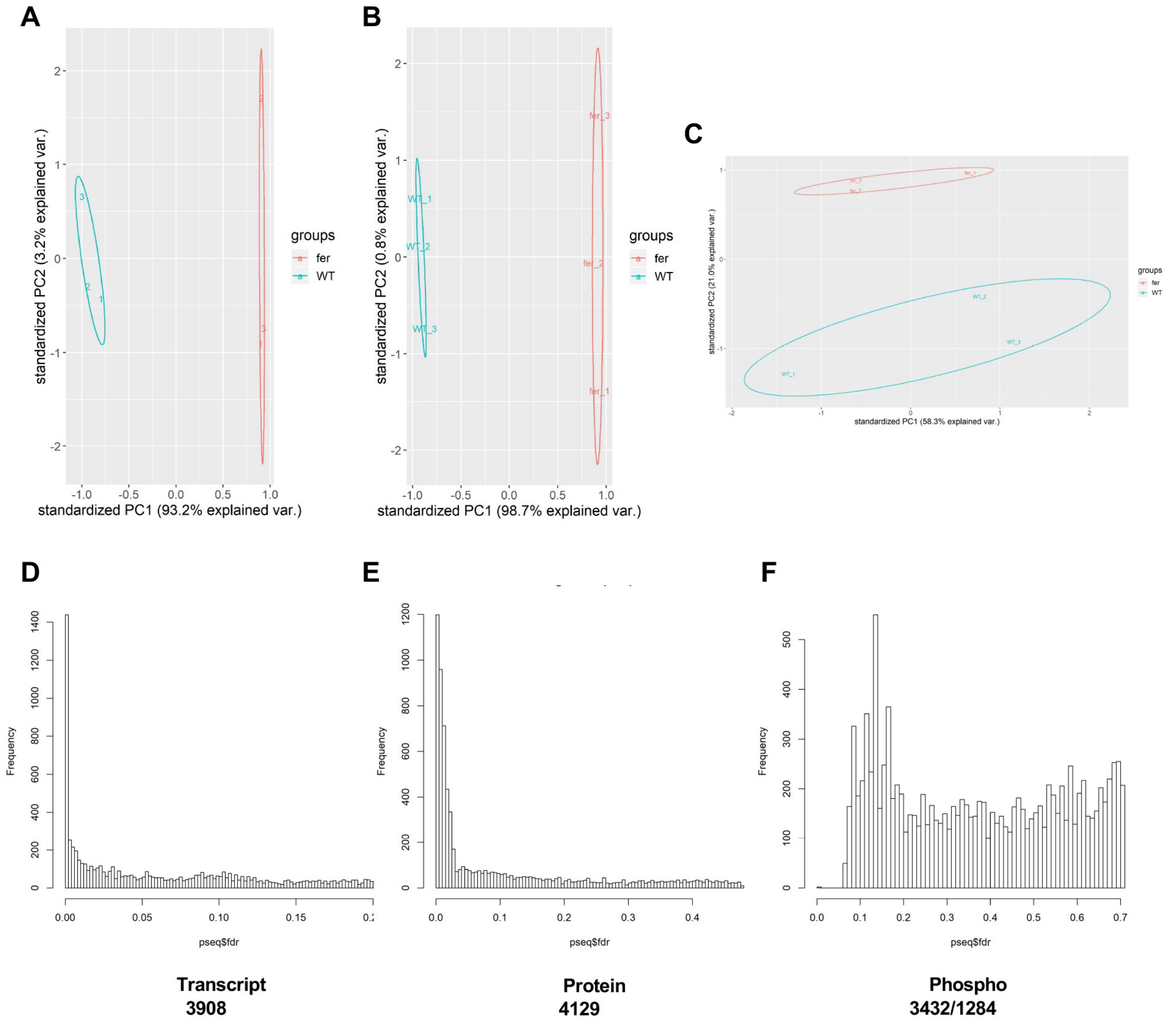

Supplemental Figure S1. Parameters of FER omics data analyses.
A-C, Principal component analysis plots. **D-F,** Distribution of *q*-values and the numbers of differentially expressed transcripts, differentially abundant proteins, and differentially phosphorylated phosphosites/proteins for the transcriptome, proteome and phosphoproteome, respectively.
(Supports Figure 1)

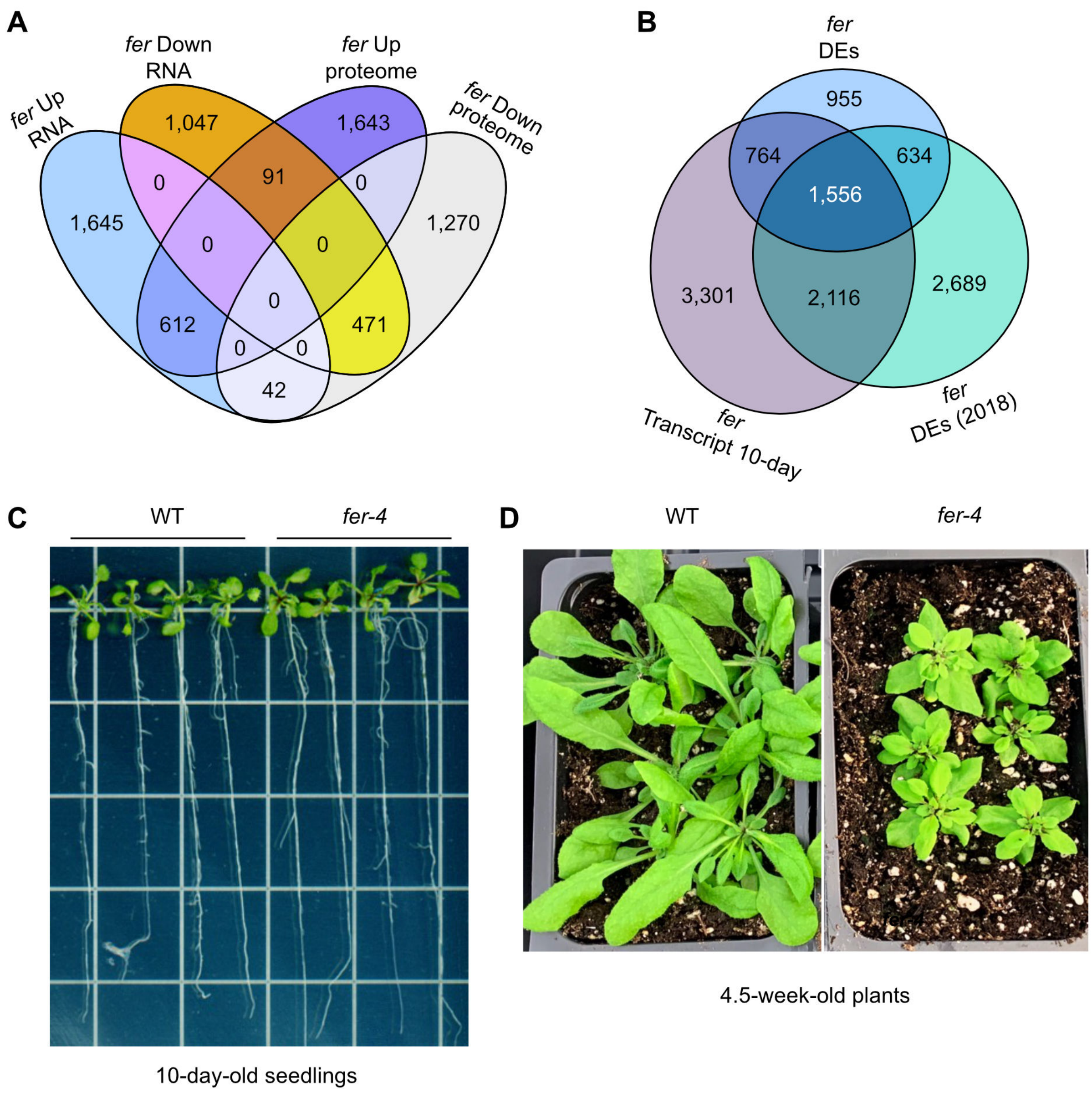

Supplemental Figure S2. Comparisons between differentially expressed transcripts and abundant proteins in *fer,* and between differentially expressed transcripts in this study and from the previous publication.
A, Venn diagram showing the extent of overlap between differentially expressed transcripts and differentially abundant proteins in the *fer* mutant. **B**, Venn diagram showing the extent of overlap between differentially expressed transcripts in 4.5-week-old *fer* (fer DEs), 10-day-old *fer* seedlings (Transcript 10-day) and published previously differentially expressed transcripts (fer DEs (2018)) (Guo et al., 2018). **C**, Representative growth phenotype of 10-day-old seedlings used for transcriptome analysis. **D**, Representative growth phenotype of 4.5-week-old plants used for multiomics analysis.
(Supports Figure 1).

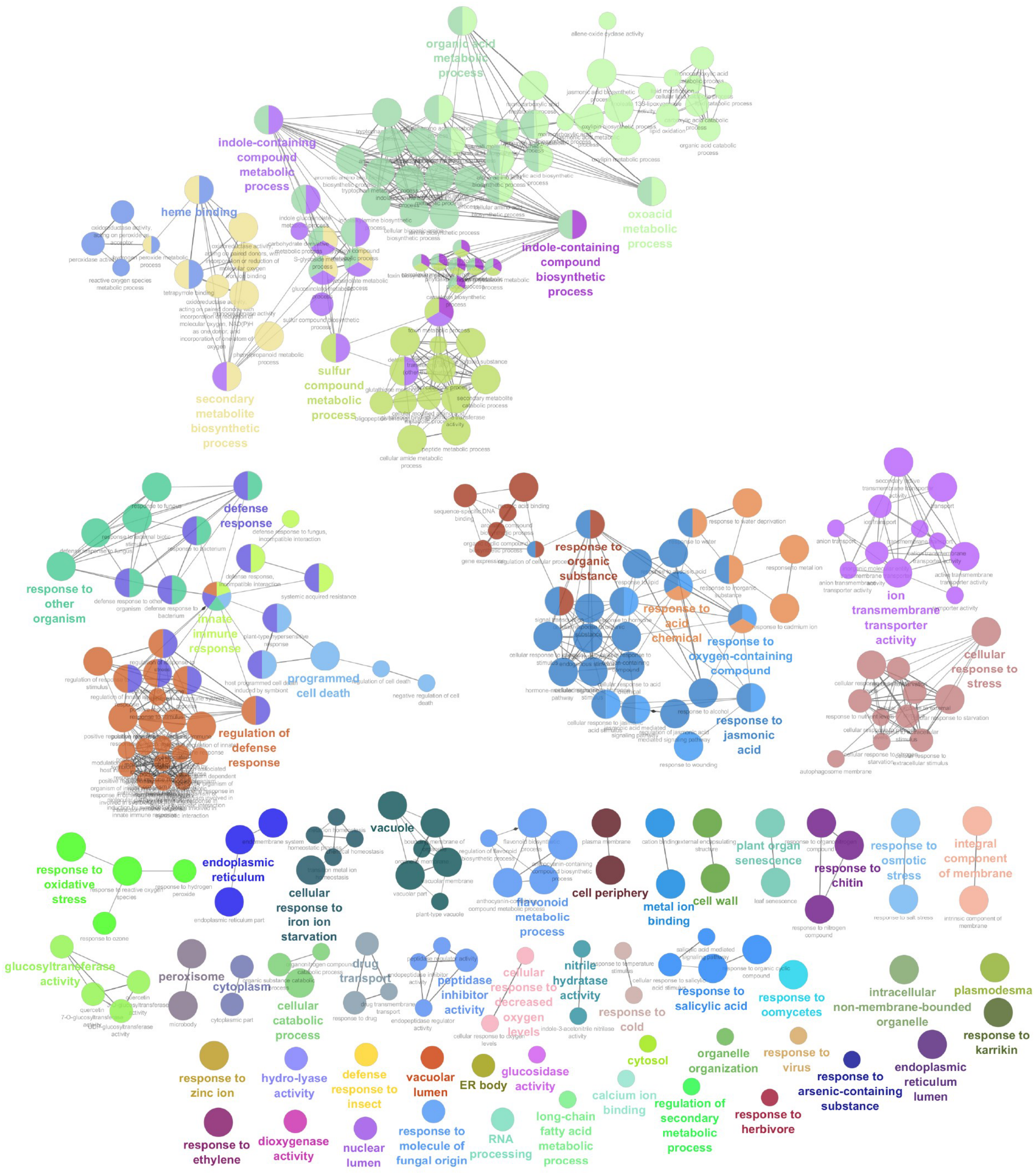

Supplemental Figure S3. Enriched GO terms in transcripts with increased levels in *fer*.
GO analysis and network reconstruction were performed using the ClueGO application in Cytoscape (Bindea et al, 2009). Terms were considered enriched with a corrected *P*-value<0.05.
(Supports Figure 1).

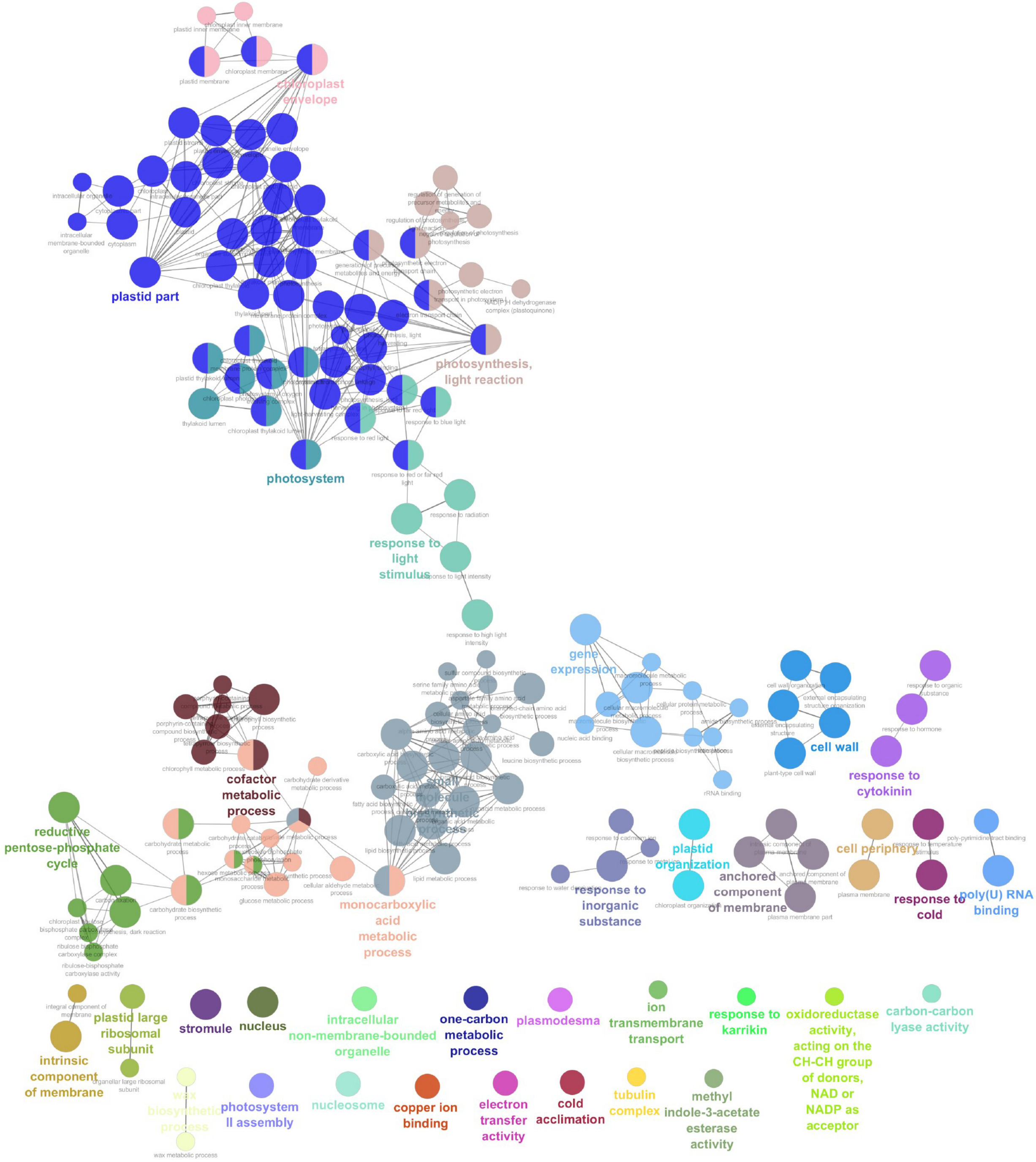

Supplemental Figure S4. Enriched GO terms in transcripts with decreased levels in *fer*.
GO analysis and network reconstruction were performed using the ClueGO application in Cytoscape (Bindea et al, 2009). Terms were considered enriched with a corrected *P*-value<0.05.
(Supports Figure 1).

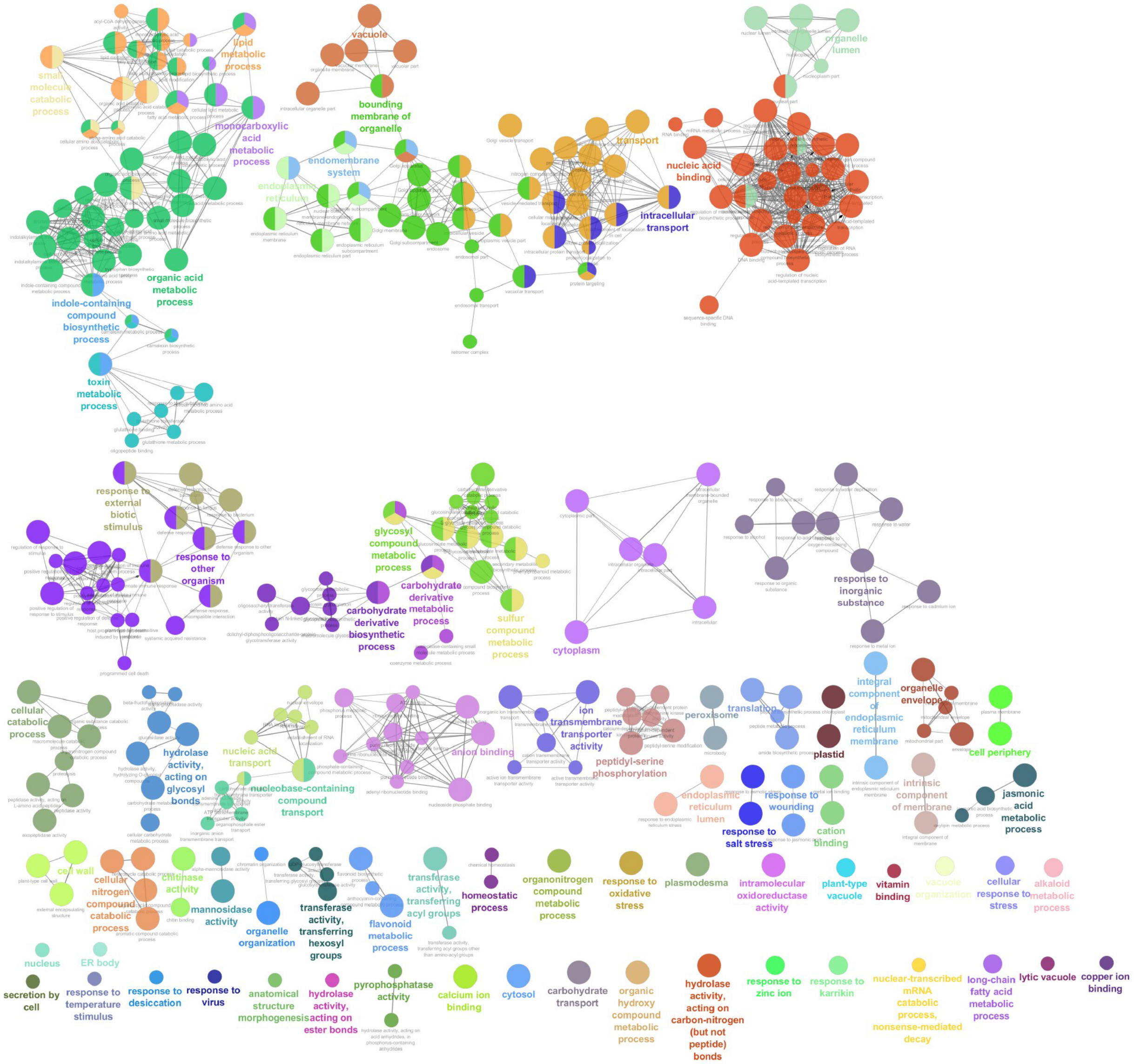

Supplemental Figure S5. Enriched GO terms in proteins with increased levels in *fer*.
GO analysis and network reconstruction were performed using the ClueGO application in Cytoscape (Bindea et al, 2009). Terms were considered enriched with a corrected *P*-value<0.05.
(Supports Figure 1).

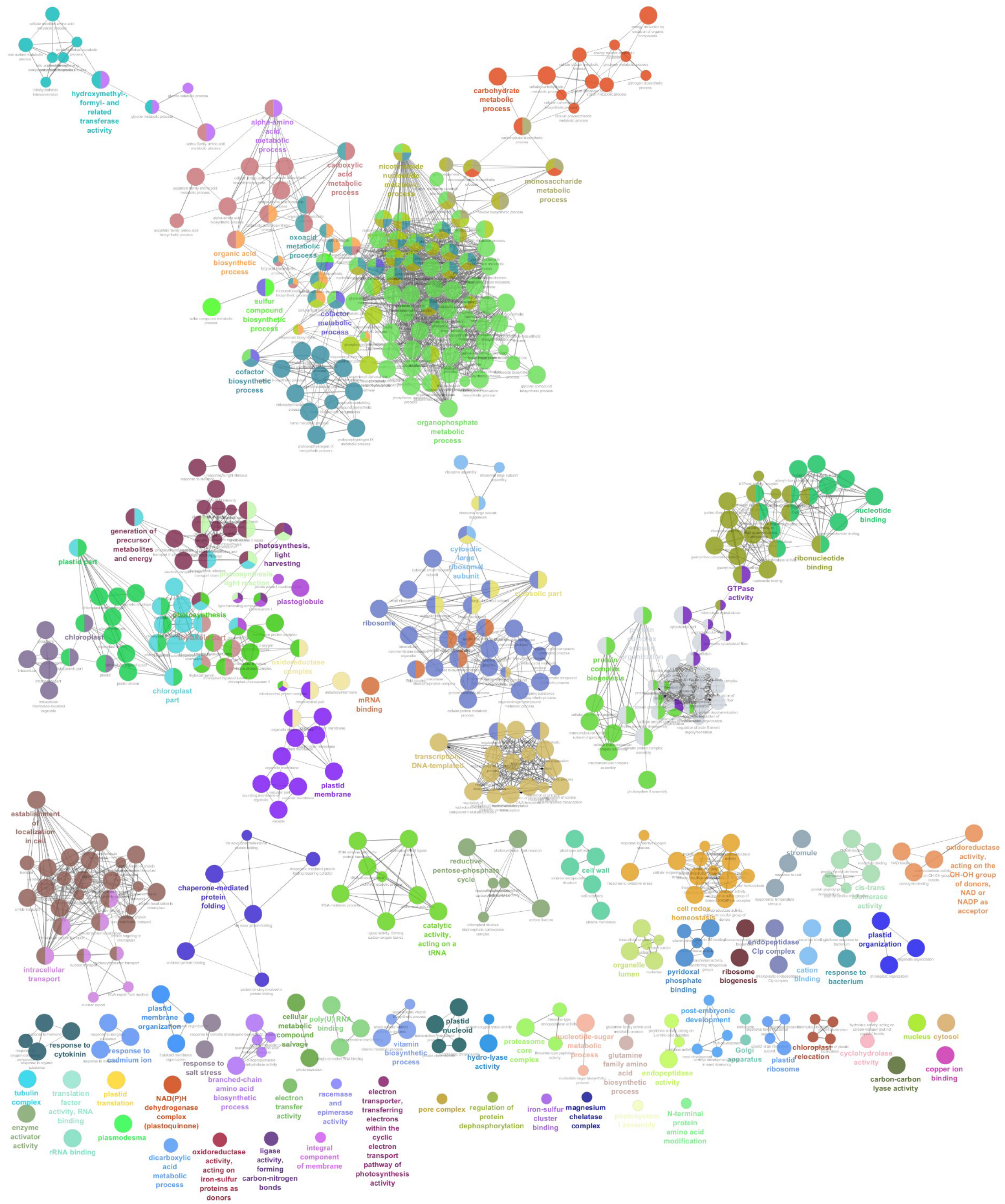

Supplemental Figure S6. Enriched GO terms in proteins with decreased levels in *fer*.
GO analysis and network reconstruction were performed using the ClueGO application in Cytoscape (Bindea et al, 2009). Terms were considered enriched with a corrected *P*-value<0.05.
(Supports Figure 1).

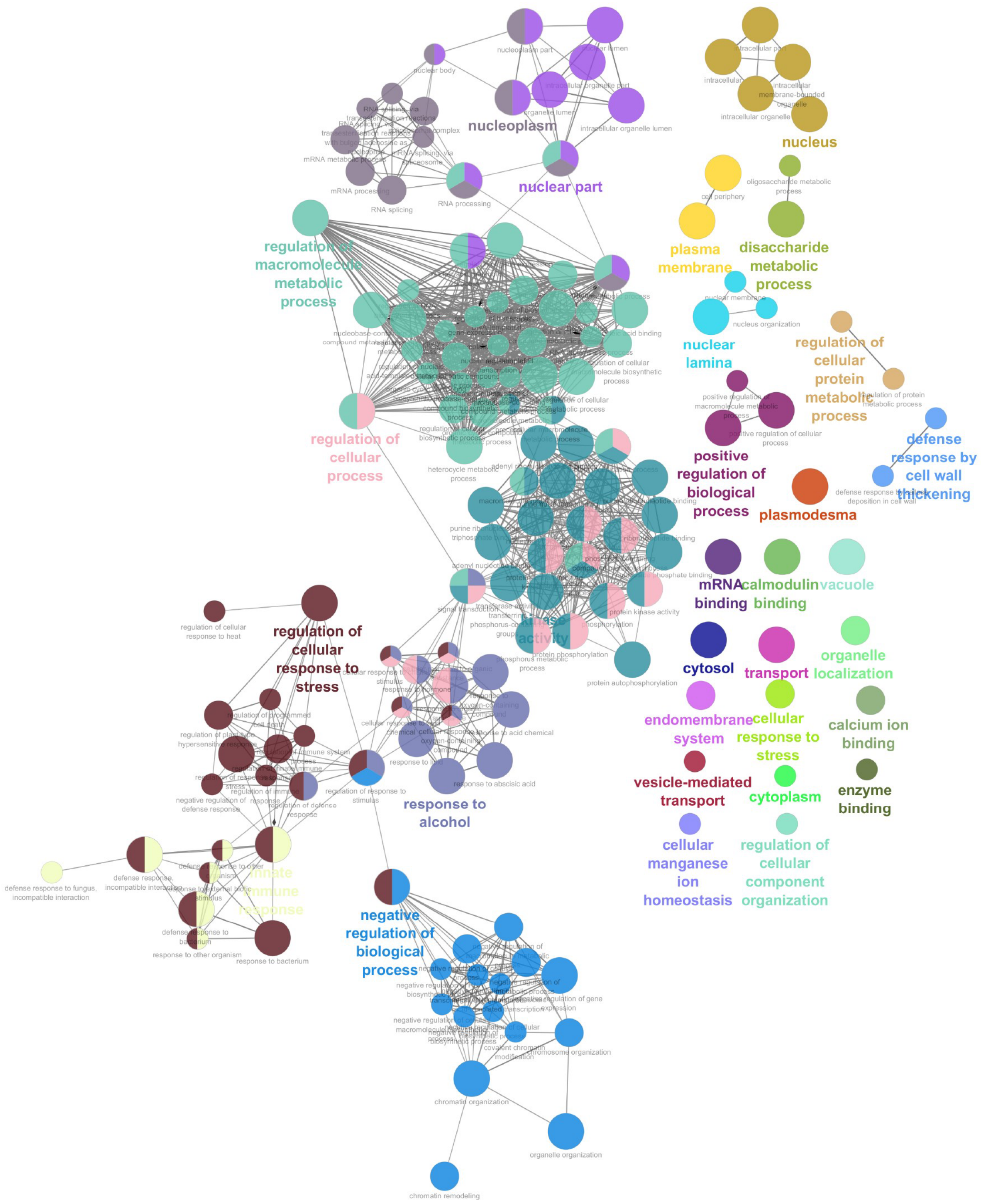

Supplemental Figure S7. Enriched GO terms in phosphoproteins with increased levels in *fer*.
GO analysis and network reconstruction were performed using the ClueGO application in Cytoscape (Bindea et al, 2009). Terms were considered enriched with a corrected *P*-value<0.05.
(Supports Figure 1).

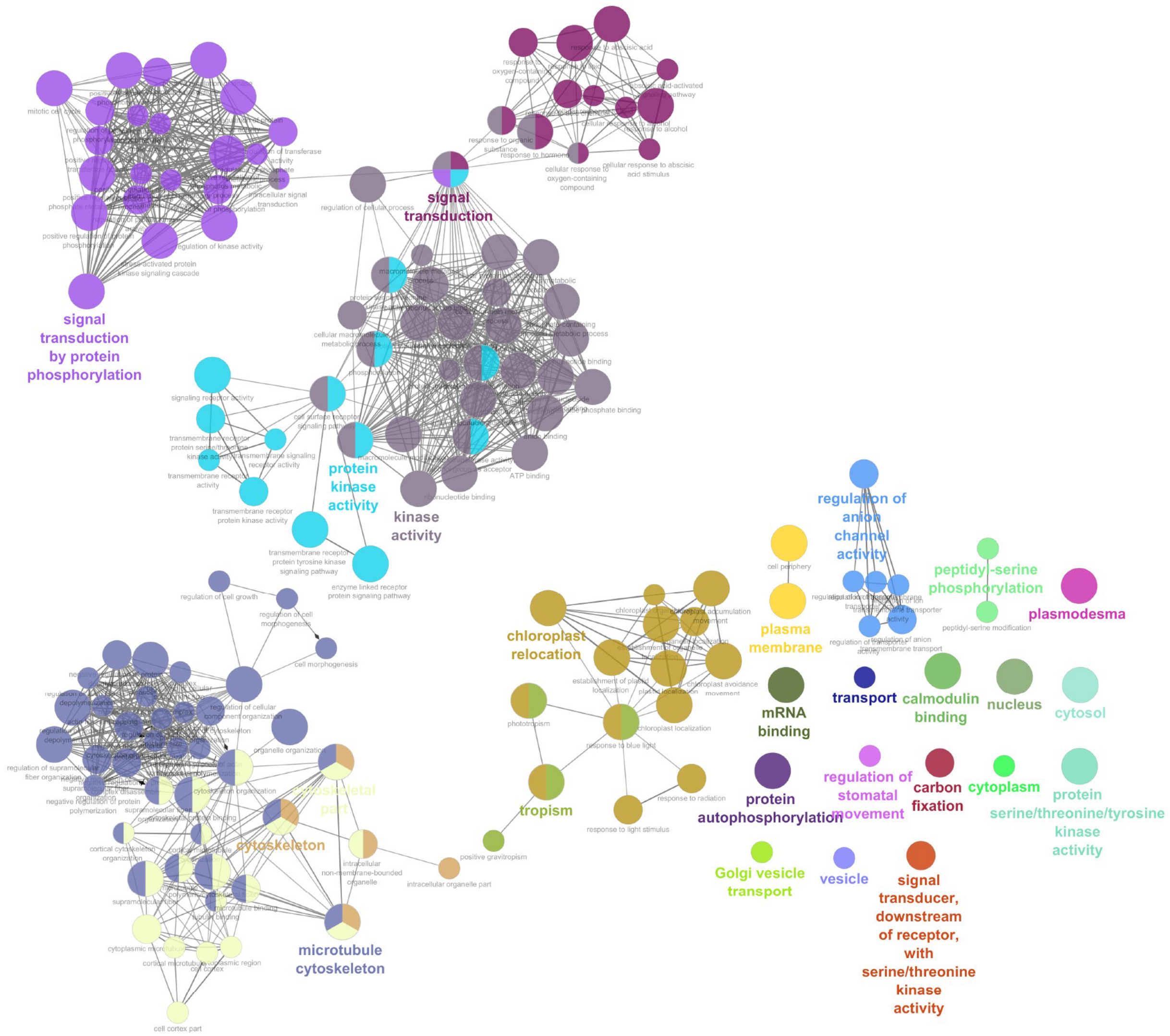

Supplemental Figure S8. Enriched GO terms in phosphoproteins with decreased levels in *fer*.
GO analysis and network reconstruction were performed using the ClueGO application in Cytoscape (Bindea et al, 2009). Terms were considered enriched with a corrected *P*-value<0.05.
(Supports Figure 1).

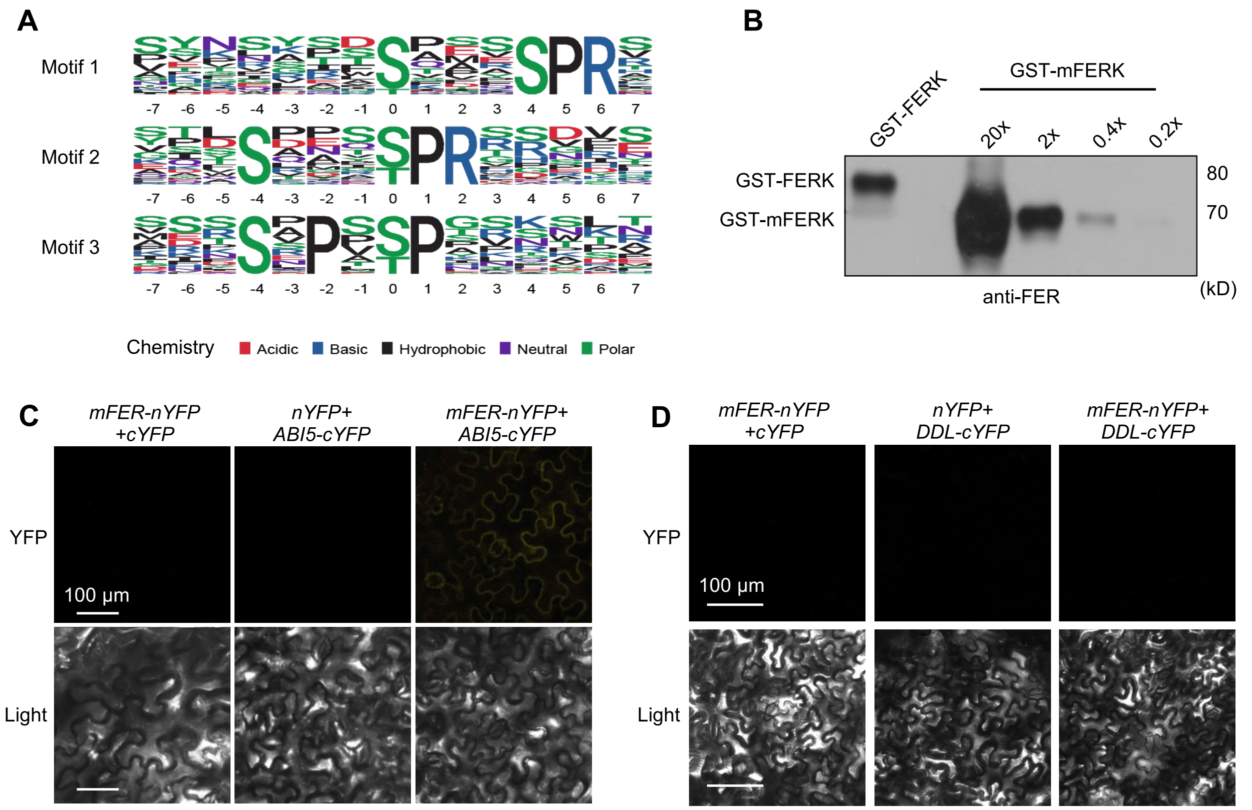

Supplemental Figure S9. FERONIA directly phosphorylates many proteins with diverse functions. **A,** LOGOs of the three most enriched consensus sequences of FER phosphorylation sites. Sequence logos were made using the ggseqlogo R package (Wagih, 2017). **B**, Amount of GST-FERK and GST-mFERK used for in vitro kinase assay was estimated by immunoblotting using anti-FER antibody. **C-D**, FER and ABI5 interaction by BiFC (**C**) and negative controls using mFER and DDL (**D**) in *N. benthamiana*. Fluorescence and light images of leaf epidermal cells are shown. Scale bars = 100 μm.
(Supports Figure 2).

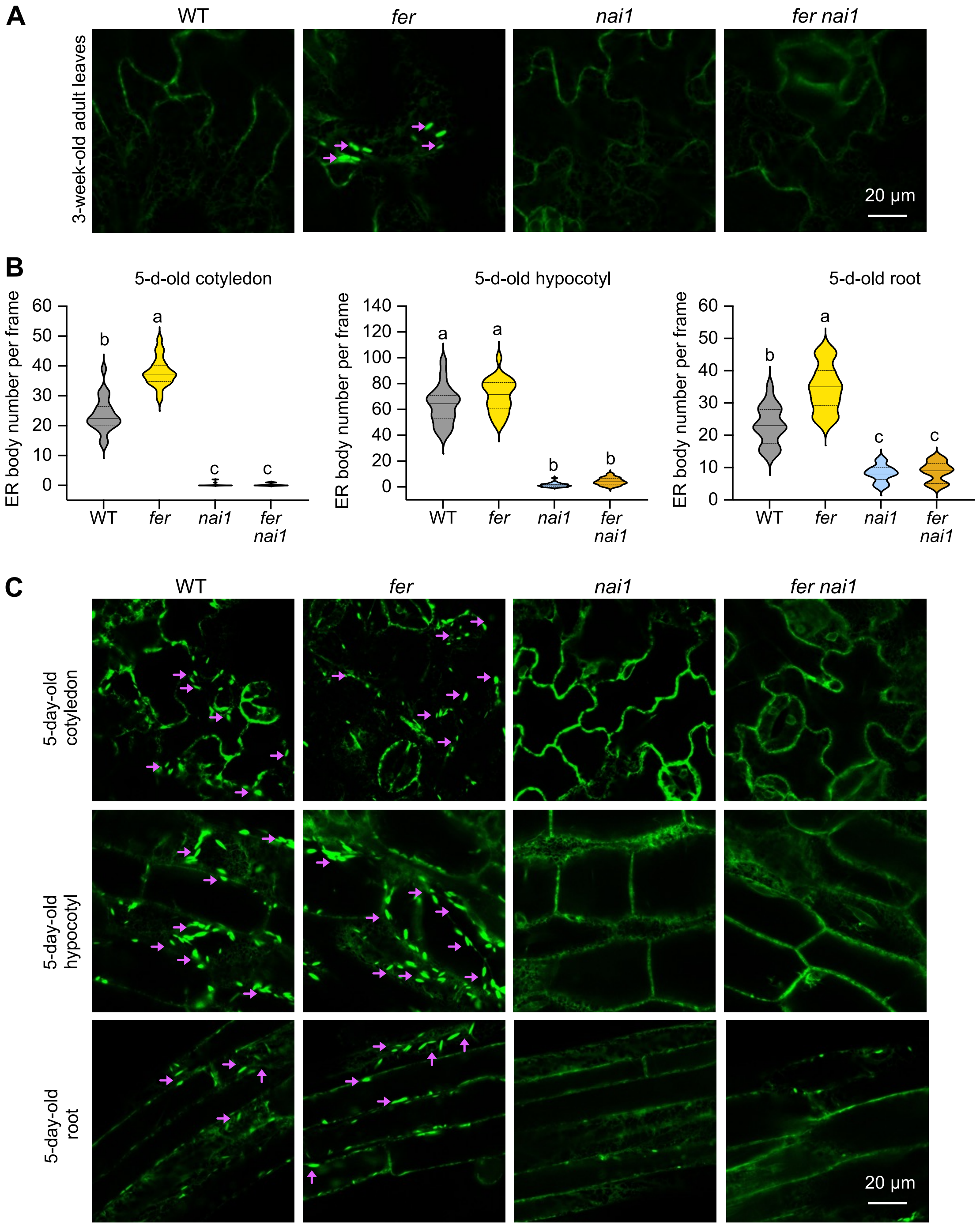

Supplemental Figure S10. FERONIA negatively regulates ER-body formation.
A, Representative confocal images of ER bodies in three-week-old rosette leaves. ER bodies are only present in *fer,* and absent in WT, *nai1* and *fer nai1* double mutants. **B,** ER bodies of 5-day-old cotyledons, hypocotyls and roots were quantified from confocal images of the four genotypes overexpressing the ER marker *GFP-HDEL.* Data are shown as violin plots with median, first and third quartiles. Different letters indicate significant differences according to one-way ANOVA Tukey's multiple range tests ($P < 0.05$). n=14-24. **C,** Representative confocal images from B. Scale bar = 20 μm.
(Supports Figure 3)

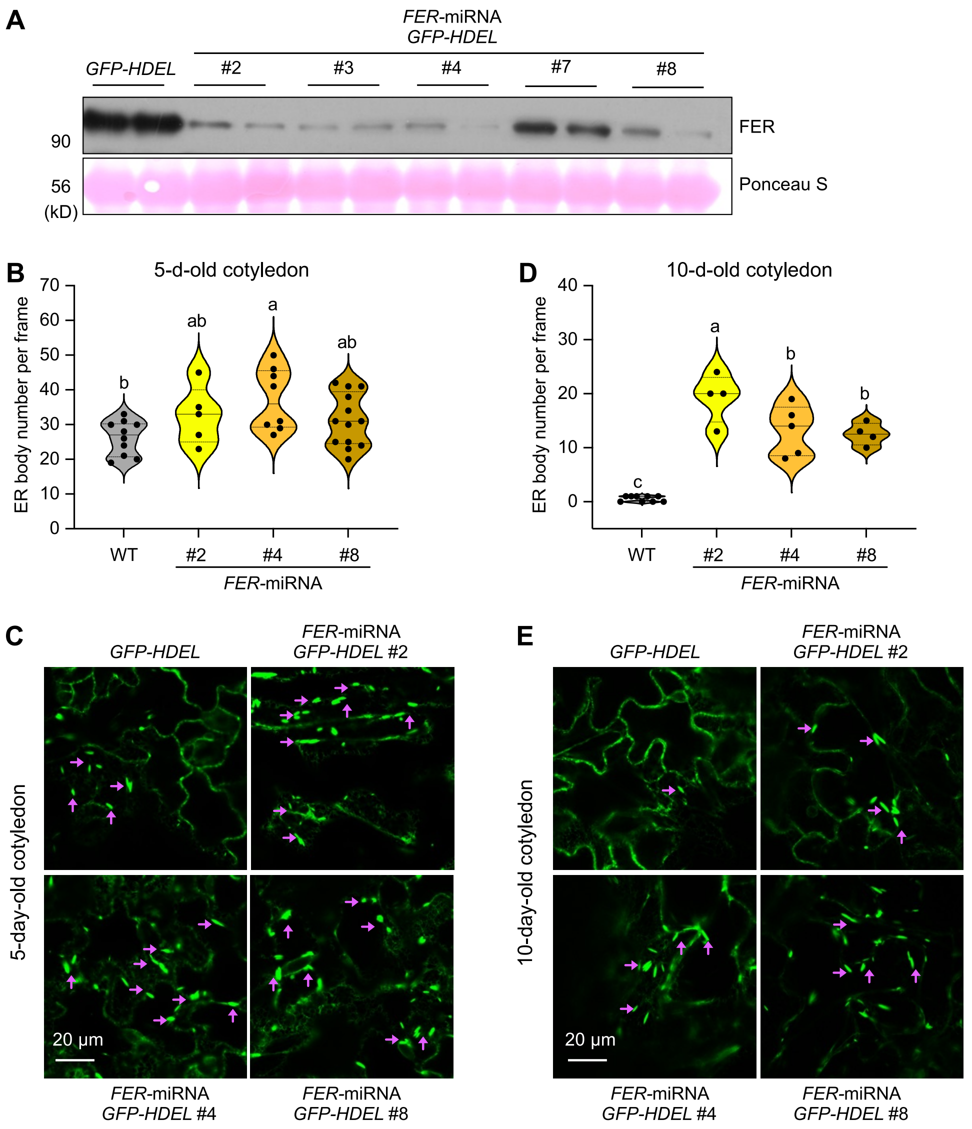

Supplemental Figure S11. FERONIA negative regulation of ER-body formation is validated using *FER*-miRNA knockdown mutant.

A, Immunoblot showing the decreased FER protein levels in multiple lines of *FER*-miRNA *GFP-HDEL.* Ponceau S staining of Rubisco protein serves as loading control. Lines #2, #4 and #8 were used for further ER body analysis. **B,** Number of ER bodies in 5-day-old cotyledons from confocal images of the control (*GFP-HDEL)* and three individual lines of *FER*-miRNA *GFP-HDEL.* Data are shown as violin plots with median, first and third quartiles and individual values. Different letters indicate significant differences according to one-way ANOVA Tukey's multiple range tests ($P < 0.05$). n=5-13. **C,** Representative confocal images from B. Scale bar = 20 μm. **D,** Number of ER bodies in 10-day-old cotyledons from confocal images of the control (*GFP-HDEL)* and three individual lines of *FER*-miRNA *GFP-HDEL.* Data are shown as violin plots with median, first and third quartiles and individual values. Different letters indicate significant differences according to one-way ANOVA Tukey's multiple range tests ($P < 0.05$). N=4-9. **E,** Representative confocal images from D. Scale bar = 20 μm.

(Supports Figure 3)

A

TFs	Target genes	Overlap with *fer* transcripts (3,908)	*p*-value
ABF1	9,213	22.6%	<0.001
ABF3	12,847	21.7%	<0.001
ABF4	9,739	22.3%	<0.001
FBH3	1,441	23.8%	<0.001

C

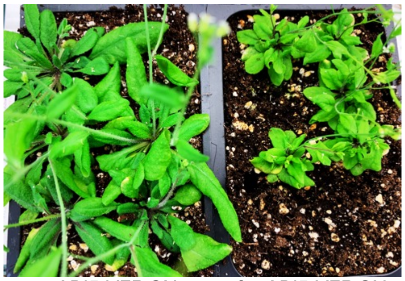

B

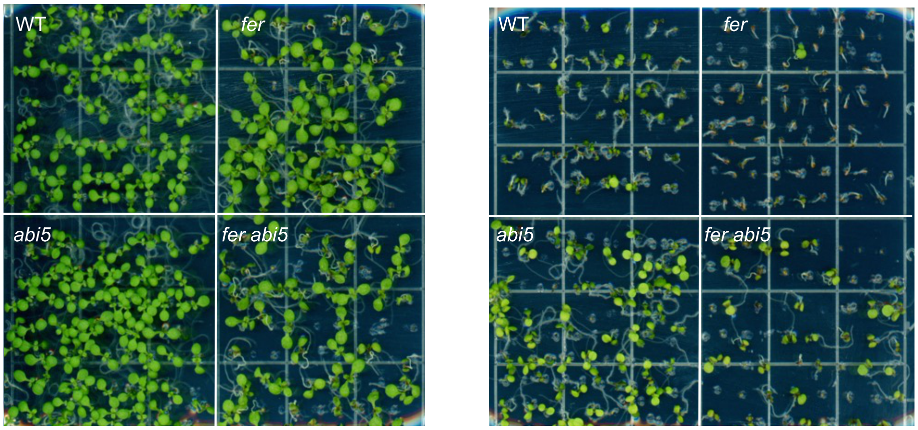

D

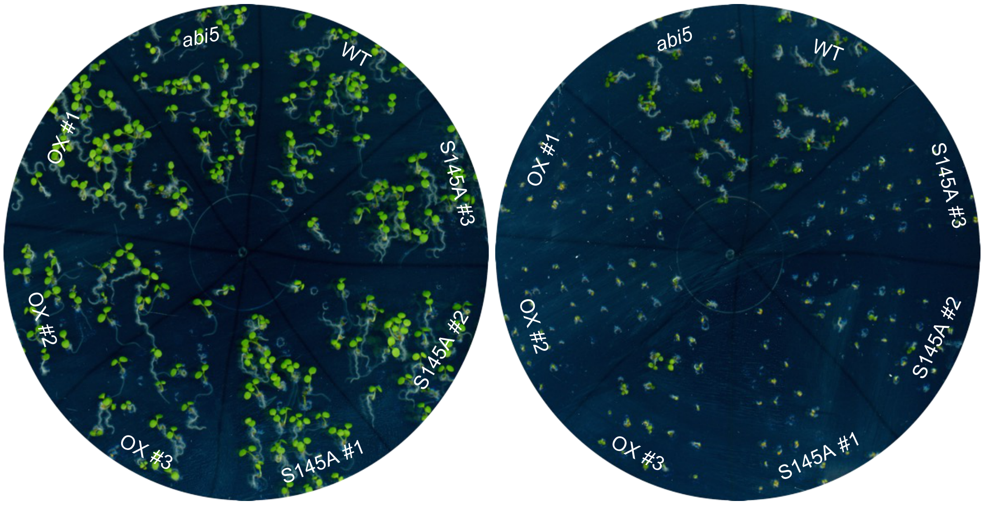

Supplemental Figure S12. FERONIA negatively regulates the ABA response during cotyledon greening through ABI5.

A, Comparisons between differentially expressed genes in *fer* and direct target genes of the ABA-responsive transcription factors (Song et al., 2016), using a hypergeometric test. **B,** Images showing seedlings of WT, *fer*, *abi5-7* and *fer abi5-7,* 5 days after germination on control or plates with 1 µM ABA. **C,** *ABI5-YFP* OX and *fer ABI5-YFP* OX plants used to assay ABI5 protein stability in Figure 7G. **D,** Images showing seedlings of WT, *abi5-7*, three *ABI5-YFP* overexpression lines (OX#1, OX#2, OX#3) and three *ABI5*^{S145A}*-FLAG* lines (S145A#1, S145#2, S145A#3), 4 days after germination on control or plates with 1 µM ABA.

(Supports Figure 7)

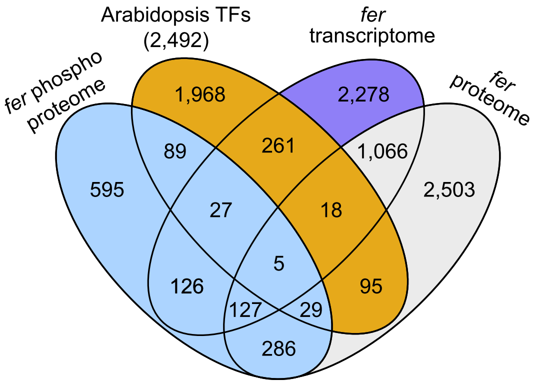

Supplemental Figure S13. FERONIA regulates over 20% of the genes encoding TFs in the Arabidopsis genome, directly or indirectly.

Venn diagram showing the extent of overlap between the 2,492 transcription factors in the Arabidopsis genome (Pruneda-Paz et al., 2014) and the differentially expressed transcripts, differentially abundant proteins and phosphoproteins in *fer*.

(Supports Figure 8)

Supplemental Table S1. Primers used in this study

Primer Name	Sequence
ABF3_BamHI_F	GCAGGATCCATGGGGTCTAGATTAAACTTC
ABF3_stop_SalI_R	GACGTCGACATACCAGGGACCCGTCAATGTCC
OXS2_BamHI_F	CGAGGATCCATGTGCTGTGGATCAGACC
OXS2_stop_SalI_R	CAGGTCGACTCAATTCTGCTGAGCCACAAGC
ABI5_BamHI_F	GCAGGATCCATGGTAACTAGAGAAACGAAG
ABI5_SalI_R	CAGGTCGACGAGTGGACAACTCGGGTTC
ABI5_stop_SalI_R	CAGGTCGACTTAGAGTGGACAACTCG
SHOU4_BamHI_F	GCAGGATCCATGGGTTCGAGATACGCATCTCACC
SHOU4_SalI_R	CAGGTCGACGACAGGAATTGCATCTACG
SHOU4_stop_SalI_R	CAGGTCGACTCAGACAGGAATTGCATCTACG
NAIP1_BamHI_F	GCAGGATCCATGTCAGAGATAGAAGAAG
NAIP1_stop_SalI_R	GACGTCGACTTAGTGAGCGTTGCGTGTGATC
FBH3_BamHI_F	GACGGATCCATGGAATCAGAATTCCAGC
FBH3_SalI_R	GACGTCGACCGCACTAGAGCATCTACATC
qMYC2_F	AACGGAGTTGTGGAAGTCG
qMYC2_R	GTTATTGTGCTTGAGCTACC
qNAI1_F	CACTGACGAGTATCTGATCG
qNAI1_R	TGAGATCACTTGTGACGTGC
qNAI2_F	CTGCAGATGGTGTATGTGG
qNAI2_R	ACTGAGCCTACCTTTGAGG
qPYK10_F	GAGAAAGGAGTGAGTCAAGC
qPYK10_R	ACTTTTCCACCGTATTCTTGG
qIGMT5_F	ATGGGACACCTAATTCCTCAAAC
qIGMT5_R	GTGGGGAGCCTAATAGCTATCT
qCYP79B3_F	AATTGAGATTGTCGGAGGACC
qCYP79B3_R	AGAGATACTCGATGTGAATACCTTC
qCYP79B2_F	TGTCGAGCTGATGGAGTC
qCYP79B2_R	AACCGGAATTGACCAAACTTG
qCYP83B1_F	GGATCTCTTATTGATTATAGCCGGTT
qCYP83B1_R	CCGTATAGCTTGGAGAGACG
qASA1_F	ATGAACGTAGCGACGATGC
qASA1_R	TCACTTACTATTGAAGCTTCCGG
qACTIN2-F	TCAACCAATCGTGTGTGACAAT
qACTIN2-R	CACCATGCTCAATAGGATACTTCAAG

Supplemental Table S2. Constructs used in this study

**Construct Name**	**Source**
pXY136 p35S:ABI5-YFP	This study
pXY384 pBRI1:ABI5-FLAG	This study
pXY136 p35S:ABI5^{S145A}-YFP	This study
pXY136 p35S:SHOU4-YFP	This study
pXY104 p35S:SHOU4-cYFP	This study
pCHF3 p35S-FLAG	This study
pXY104 p35S:cYFP	Yu et al., 2008
pXY104 p35S:FBH3-cYFP	This study
pXY104 p35S:ABI5-cYFP	This study
pXY104 p35S:DDL-cYFP	This study
pXY103 p35S:nYFP	Yu et al., 2008
pXY103 p35S:mFER-nYFP	Guo et al., 2018
pXY384 pBRI1:FER-FLAG	This study
pYY46 p35S:FER-GFP	Guo et al., 2018
pYY46 p35S:mFER-GFP	Guo et al., 2018
pMBP-H MBP	Guo et al., 2018
pMBP-H MBP-ABI5	This study
pMBP-H MBP-ABI5^{S145A}	This study
pMBP-H MBP-ABF3	This study
pMBP-H MBP-OXS2	This study
pMBP-H MBP-SHOU4	This study
pMBP-H MBP-NAIP1	This study
pDESTHisMBP	Addgene#11085
pDESTHisMBP-FBH3	This study
pDESTHisMBP-GT2	This study
pDESTHisMBP-NSI	This study
pDESTHisMBP-At5g51950	This study
pGEX-5x-1 GST-FERK	Guo et al., 2018
pGEX-5x-1 GST-mFERK	Guo et al., 2018
