# Supplementary material for: Integrated omics reveal novel functions and underlying mechanisms of the receptor kinase FERONIA in Arabidopsis thaliana
Source: Plant Cell. 2022 Apr 18;34(7):2594–614. doi: 10.1093/plcell/koac111 (PMC9252503; doi:10.1093/plcell/koac111)
Supplement: koac111_Supplementary_Data [file Peer Review Report TPC2021LSB00634R2.pdf › koac111-suppl_data/Peer Review Report TPC2021LSB00634R2.pdf]

Integrated Omics Reveal Novel Functions and Underlying Mechanisms of FERONIA Receptor Kinase in *Arabidopsis thaliana*

Ping Wang, Natalie M Clark, Trevor M. Nolan, Gaoyuan Song, Parker M. Bartz, Ching-Yi Liao, Christian Montes-Serey, Ella Katz, Joanna K. Polko, Joseph J. Kieber, Daniel J. Kliebenstein, Diane C Bassham, Justin W Walley, Yanhai Yin and Hongqing Guo

Corresponding authors: *Yanhai Yin* *yin@iastate.edu*

Hongqing Guo *hguo@iastate.edu*

Review timeline:

**TPC2021-LSB-00634**	Submission received:	Jul. 23, 2021
	1st Decision:	Sep. 20, 2021 *Request revisions*
**TPC2021-LSB-00634R1**	Revision received:	Jan. 20, 2022
	2nd Decision:	Feb. 15, 2022 *Accept (minor revisions)*
**TPC2021-LSB-00634R2**	Revision received:	Feb. 22, 2022
	3rd Decision:	Feb. 28, 2022 *Accept*
	Final acceptance:	Apr. 08, 2022

REPORT: (The report shows the major requests for revision and author responses. Minor comments for revision and miscellaneous correspondence are not included. The original format may not be reflected in this compilation, but the reviewer comments and author responses are not edited, except to correct minor typographical or spelling errors that could be a source of ambiguity.)

**TPC2021-LSB-00634**	**Submission received**	**Jul. 23, 2021**

**TPC2021-LSB-00634**	**1st Editorial Decision – *Request revisions***	**Sep. 20, 2021**

We have received reviews of your manuscript entitled "Integrated Omics Reveal Novel Functions and Underlying Mechanisms of FERONIA Receptor Kinase in *Arabidopsis thaliana*." Thank you for submitting your best work to The Plant Cell. The editorial board agrees that the work you describe is substantive, falls within the scope of the journal, and may become acceptable for publication, pending revision and re-review.

We ask you to pay attention to the following points in preparing your revision: Please strengthen the genetic and functional analysis of ABA-related phenotypes, and ER body phenotype differences, as raised by Reviewers 1 and 2; clarify the in vivo, in vitro phosphorylation, direct or indirect phosphorylation between FER and NAI1/2, TOR1 and ABI5 as questioned by all three reviewers. In addition to gene IDs, we ask that you deposit all the proteomic data at ProteomeXchange for public access as Reviewers 2 & 3 suggested. I should stress that we are reluctant to see manuscripts undergoing multiple rounds of revision and would be unlikely to offer you more than one chance to satisfy the reviewers.

**TPC2021-LSB-00634R1**	**1st Revision received**	**Jan. 20, 2022**

-- Reviewer comments (with **Authors responses in** blue):

Summary of Response

We would like to thank you for your consideration of our manuscript and thank all the reviewers for the critical and constructive comments and suggestions on the manuscript. We have performed additional experiments and/or analyses and are able to address most of reviewers' comments. As

suggested by reviewers 1 and 3, we focused on FER functions in endoplasmic reticulum (ER)-body formation, glucosinolate biosynthesis and abscisic acid (ABA) response, and removed the description of FER regulation of autophagy from the revised manuscript.

We were also able to obtain *fer*-complemented transgenic lines with wild type *FER*, *pFER:FER-GFP* and mutant *FER* (K565R), *pFER:FER^{K565R}-GFP* from Prof. Sally Assmann and demonstrated that FER kinase activity is indeed required for all three FER functions we examined. We performed in vitro kinase assays with mutant kinase FERK^{K565R} and demonstrated that the mutant kinase does not phosphorylate the identified substrates at similar protein concentration to the wild-type FER Kinase, FERK. It is worth noting that high levels of mutant kinase, FERK^{K565R} (100-200-fold higher than wild-type FERK) showed autophosphorylation, which might explain that high levels of FER^{K565R}-GFP transgene expression was able to complement *fer-4* growth phenotype and ABA response/ER-body/IG biosynthesis phenotype and gene expression.

In addition to that of 4.5-week-old adult plants, we included RNA-seq analysis of 10-day-old seedlings and also performed quantitative PCR with 3-week-old plants to show FER-regulation of the expression of ER body and glucosinolate biosynthesis genes is consistent in both young and adult plants. We also validated some FER-substrate interactions by Co-IP and/or BiFC assays; and wanted to emphasize that some FER-substrate interactions (such as FER-ABI5) may be very transient and cannot be detected by all current assays we have.

We performed additional functional analysis on FER regulation of ABI5 with the FER kinase mutant, FER^{K565R}. We clarified other places as appropriate. We deposited all the omics data to public databases. We also re-created all the graphs with GraphPad Prism software. We changed the order of the authorship and added new authors due to new research performed during the revision.

We numbered the comments and responses to facilitate tracking.

Reviewer comments:

Reviewer #1 (Comments for the Author):

Comment 1: Based on quantitative transcriptome, proteome, and phosphoproteome profiling of the *fer* mutant, authors found many potential functions of FER. They further confirmed that FER is required for endoplasmic reticulum body formation, glucosinolate biosynthesis, autophagy and germination using biochemical and genetical approaches. This study indeed provides new insights into the underlying mechanisms of FER function. The omics data on fer will be a useful resource to study FER. However, data in this manuscript are not supportive for the conclusions that authors have drawn, and authors should provide more strongly evidence to clarify each new function of FER.

In general, the major problem of this work is that the authors want to convey too much information in one paper. The result is that this paper is loosely formed and the functional studies of FER are not well supported by solid enough data. So, I suggest that the authors delete parts of functional studies of FER (such as, ABA, FER-TOR role in autophagy) and only focus on one or two functional studies, such as the formation of ER bodies.

Response 1: We thank the reviewer for the constructive comments and suggestions. We removed the FER-TOR study and focused on FER-ER body/indolic glucosinolates and FER-ABI5 in the revision.

Major：

Comment 2: Fig.1. How could the phosphorylation level increase when carried out phosphoproteome analysis in *fer-4* mutant?

Response 2: Having both increased and decreased phosphorylation sites is typically observed when profiling kinase loss-of-function mutants. It is likely that other protein kinases become more active (or phosphatases become less active) in *fer-4* mutant to cause the observed elevated phosphorylation; The potential FER substrates we chose for further analysis are proteins with decreased levels of phosphorylation in *fer-4* mutant.

Comment 3: Authors declared that 29% of the differentially expressed proteins (DEPs) also exhibited differential expression of their cognate transcripts in *fer*, with most of the DEPs following the same direction of change as their transcript. However, no change trends of the same direction were observed in Fig1.

Response 3: It is shown in Supplemental Figure S2A, and the name of the figure is added at the end of the sentence in lines 115-117.

Comment 4: Also, could you please explain the reason why when the transcription levels changed, the phosphorylation level of proteins changed as well. Are there any inner links between transcription level and phosphorylation level of proteins? How could the authors ensure a good biological repetition?

Response 4: Transcript and phosphorylation level can change in the same direction for many reasons. These include coordinated regulation of processes that control steady state transcript levels (i.e. by transcription and/or mRNA turnover) and phosphorylation state (i.e. through action of a kinase or phosphatase). Additionally, for proteins that phosphorylation level largely follows the amount of protein present, this observation can simply be a reflection of a change in transcript level resulting in a change in the level of the genes' proteins. We used three independent biological replicates and reported gene expression changes that are biologically reproducible.

Comment 5: The PCA analysis in Fig S1 did not show a good biological repetition in WT and *fer-4* mutant.

Response 5: We interpret the PCA results as actually indicating good overall replication between replicates. For transcript and protein, PC1 explains 93 and 98 percent of the variance. There is little spread among the replicates for PC1, which explains the vast majority of the variance. There is more spread among the replicates in the phosphorylation data. However, the replicates still cluster by genotype. Overall, we were able to identify statically significant changes in gene-product levels, which would not have been possible if the biological repetition between replicate samples was poor.

Comment 6: The overall data in Fig1A was not consistent with data in Figure 1B, Figure S2A, Figure S12A and Figure S15. The number of differentially expressed protein in Fig 1b was 4129, but it was 4133 in the description of the text (line 88).

Response 6: Sorry for the error we made in the numbers. We changed the number of differentially expressed proteins from 4133 to 4129 (2346 UP, and 1783 DN) in Figure 1A and Supplemental Figure S1 and in the text (lines 103-105). The comparisons in FigS2A, FigS2B, Fig S15 (now Supplemental Figure S13) are all based on the number 4,129. We also changed the number of differentially phosphorylated proteins from 1394 to 1284 in Figure 1A and Supplemental Figure S1 to reflect that 110 proteins have peptides with both increased and decreased phosphorylation. The former Fig S12A was on FER-TOR study and removed from the revision.

Comment 7: Fig.2. The presented conserved motifs of FER phosphorylation substrates obtained based on the sequence of these phosphorylated polypeptides was of little instructive value, for the reason that proteins of reduced phosphorylation levels in *fer* were not exactly the direct phosphorylation substrates of FER.

Response 7: Out of the nine proteins we chose for validation of the in vivo phosphoproteomics data, seven showed direct phosphorylation by FER kinase. We decided to move motif LOGO to Supplemental Figure S9A.

Comment 8: Concerning in vitro kinase assay (Figure 2C), was the same amount of FERK added in the reaction solution? Why did the lanes show different abundance of FERK in autoradiograph?

Response 8: The same amount of FERK was added to each reaction. It is possible that interactions between FERK and the substrate proteins affected FERK autophosphorylation.

Comment 9: The FERK and selected potential substrates of FERK were not well purified, which could not meet the requirements for verification of kinase activity in vitro.

Response 9: We used SYPRO RUBY staining for the proteins, which is very sensitive for protein staining and can show small amounts of degradation products or contaminants. We ran the same proteins and stained with Coomassie blue (see Figure below).

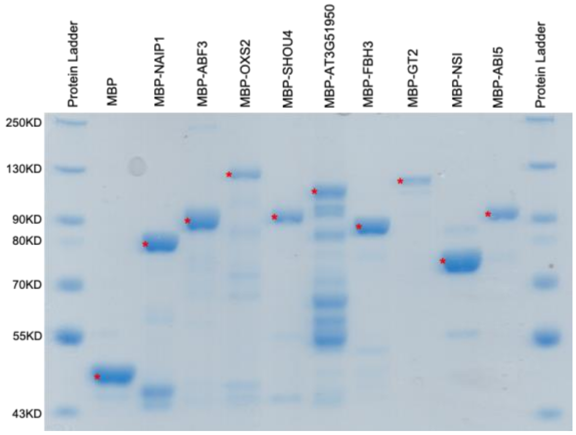

Comment 10: Based on information in Fig2C, it could only draw a conclusion that FER could phosphorylate these proteins in vitro. It was strongly recommended that ferK565R should be used as a control. Alternatively, the mutant of identified phosphorylated sites of these proteins could be added to further confirm if these proteins could be phosphorylated by FER.

Response 10: The proteins tested here were detected with decreased phosphorylation in vivo in the *fer-4* mutant via phosphoproteomic analysis, which suggested that these proteins are FER substrates in vivo. The in vitro kinase assay was to validate the in vivo findings.

In the revision, we used mutant kinase $FERK^{K565R}$ as control and repeated in vitro kinase assay with three of the substrates. While WT FERK phosphorylated the substrates, similar amount of FERK565R did not show either autophosphorylation or phosphorylation of the substrates (Figure 2C, Lines 166-178).

Comment 11: Fig.3. The authors should explain how FER (localized to PM) modulates an ER-localizing protein NAI1 to regulate ER body.

Response 11: NAI1 is a transcription factor that is required for ER body formation. It was shown to be localized in the nucleus (Sarkar et al 2021). We detected increased transcript level of *NAI1* in *fer-4* mutant, and no protein or phosphorylation of NAI1 were detected. Our BiFC and co-immunoprecipitation assays did not detect FER and NAI1 protein interaction. It is conceivable that FER regulates intermediate proteins that in turn regulate *NAI1* transcription.

Comment 12: Based on the previous results that ROP2-TOR interaction, FER-GEFs/ROP2 interaction, authors claimed that FER functions in the same complex with ROP2 and TOR, and activates TOR kinase. I think it is not enough to draw the conclusion. Do FER and TOR form a protein complex? Do FER and TOR directly interact in vitro or in vivo? The authors claimed that FER negatively regulates autophagy, what is the phenotype of *fer* mutant under nutrient-deprivation conditions? Further, anti-ATG8 should be used to detect ATG8 and ATG8-PE levels in *fer* mutant.

While anti-P-S6K1/2 antibody only recognized the phosphorylated S6K1/2, anti-S6K1/2 antibody recognized both phosphorylated and non-phosphorylated S6K1/2. Has it been reported in the literature?

The authors draw the conclusion that FER functions in the same complex with ROP2 and TOR, and activates TOR kinase. However, no data in Figure 6 supported that ROP2 involved in FER-TOR signaling pathway.

The *fer TOROE* material may not be enough to support the conclusion that FER acts through TOR to negatively regulate autophagy. I suggest to add the material *fer-4* crossing with *es-tor*.

Response 12: We removed the FER-TOR-autophagy section from the revised manuscript as suggested also by Reviewer #3.

Comment 13: Authors claimed that FER regulates ABA-mediated seed germination through ABI5, but authors quantified cotyledon greening rate in Fig.7C, could you please explain why the cotyledon greening rate but not the seed germination rate was calculated here?

Response 13: ABI5 negatively regulates cotyledon greening (Guan, et al. 2014). We changed the wording from "germination" to "cotyledon greening" in the text.

Comment 14: More systematic transgenic plants were needed to prove that the regulation of ABA signal pathway was mediated by FER-ABI5, such as *fer/ABI5S145A-OE*. Moreover, *fer/ABI5-OE* and *fer/ABI5S145A-OE* should be added to Fig7F to further confirm the regulation of FER in stability of ABI5.

Response 14: To further confirm the negative regulation of FER on ABI5 protein stability, we co-expressed *ABI5-FLAG* with *FER-GFP*, *mFER-GFP* or *GFP* control. Two days post infiltration, we collected leaf samples and carried out western blotting. It showed much lower levels of ABI5-FLAG when FER-GFP was present compared to that of mFER-GFP and control (Figure 7F, Lines 354-359), suggesting that FER destabilizes ABI5 and the destabilization is dependent on FER kinase activity.

Minor:

Comment 15: For *ABI5-YFP OX* and *fer ABI5-YFP OX*, could you please explain why the specificity of *ABI5-YFP OX* and *fer ABI5-YFP OX* shown in Fig7D was much less than that in Fig7F?

Response 15: we used different batches of polyclonal anti-GFP antibody from Invitrogen for Fig7F (now Figure 7I) and Fig7D (now Figure 7G). The batch for Fig7D (now Figure 7G) gave some non-specific bands.

Comment 16: Could you explain why 4.5-week-old plants, instead of younger seedlings, were used in multi-omics? And why 3-week-old whole rosettes were used in Aliphatic glucosinolates (AG) biosynthesis?

Response 16: The reason that 4.5-week-old plants were used for the multi-omics was that *fer-4* shows the strongest growth phenotype at this age.

In the transcriptome data of 10-day-old seedlings (Supplemental Data Set S4, deposited to the public data base), the IG gene expression pattern is consistent with that of the 4.5-week-old plants (Figure 5A, 5B), which suggests that FER regulation of the IG biosynthesis is consistent in different stages of plant growth.

We further checked the expression of some key genes in IG biosynthesis in 3-week-old plants using qPCR, and the expression pattern is consistent with that of seedlings and 4.5-week-old plants (Figure 6A, Lines 306-318).

Comment 17: Line 618-619: How could the authors ensure the accuracy if sequencing was conducted by HiSeq 4000 with 50-bp single end reads.

Response 17: 50-bp reads are routinely used for transcriptome profiling in Arabidopsis and we only used the reads that uniquely mapped to a transcript for quantification. We have added this following text to the materials and methods on lines 600-608 for clarity:

> *"Libraries were run on a HiSeq4000 with 50bp reads as QuantSeq is optimized for 50 to 100bp reads (Moll et al., 2014). Each library was run twice to increase the average read count to approximately 6 million reads per sample. Read alignment to the TAIR10 genome was performed using the STAR aligner (Dobin et al., 2013) using default parameters except --outFilterMismatchNoverLmax was set to 0.6 to account for the shorter QuantSeq reads. This resulted in approximately 70% of reads uniquely mapped per sample which is expected for QuantSeq (Moll et al., 2014). Counts per transcript were obtained using htseq-count using the intersection-nonempty parameter for reads spanning more than one feature (Anders et al, 2014)."*

Comment 18: Line 226-228, line 236-239: the expression levels of many IG biosynthesis genes and the genes that are involved in both IG and AG are increased in *fer-4* at both transcript and protein levels (line 226-228), however, many AG biosynthesis genes had decreased levels of both transcripts and proteins in the *fer-4* mutant (line 236-239). It was inconsistent in results authors had obtained and it could be drawn a conclusion that FER did not regulate the biosynthesis of AG.

> **Response 18**: We thank the reviewer for the suggestion. It has been shown that ER body formation has a strong co-occurrence with IG biosynthesis (Nakano *et al.*, 2017; Wang *et al.*, 2017). It makes more sense to address IG biosynthesis along with ER body formation. FER's role in AG biosynthesis is likely more complex and was removed from the revised manuscript.

Comment 19: Line 455-458: The altered levels of transcript, protein of TFs may be due to the indirect effects of FER. It is not precise to conclude that FER regulates diverse biological processes through extensive involvement of the transcription factors.

> **Response 19**: We modified the sentence to reflect both direct and indirect regulation of FER on the TFs in line 439.

Comment 20: Tables S6 did not show the corresponding Venn region that each transcription factor belongs to.

> **Response 20**: It is shown in Supplemental Figure S13.

Comment 21: Line 230, 233 and 239: don't and doesn't should be modified. Line 173: It should be modified.

> **Response 21**: They are modified, now in line 300, line 303, and line 224.

Comment 22: Add statistics in Figure S12 for B and D.

> **Response 22**: Figure S12 on FER-TOR was removed.

Comment 23: Check *fer* and *fer-4* in the manuscript and keep using a uniform form.

> **Response 23**: We made the changes.

Comment 24: In Fig D and F, the LC should be presented in same color.

> **Response 24**: Corrected.

Comment 25: Line 42-43: Please add a reference (Chen et al, 2016, PNAS).

Response 25: Added, it is now in line 51.

Comment 26: Line443-447: Please change "FERONIA" to "FER" to keep the consistency with the previous description in the text.

Response 26: The paragraph on FER-TOR was removed. And we used FER throughout the text.

Comment 27: Line399-400, line 415-417: Please add references here.

Response 27: Added in line 392, and lines 408-409.

Comment 28: It would be necessary to have molecular mass markers in Fig6F and Fig6G.

Response 28: The figure on FER-TOR is removed.

Comment 29: Please delete the background lines in Fig3C, Fig3D, Fig4A, Fig4C, Fig5C, Fig5D, Fig5E, Fig6B, Fig6E, Fig7C. It was strongly recommended that authors could use some software such as Prism for illustration.

Response 29: We re-created all the graphs with GraphPad Prism software as you suggested.

Reviewer #2 (Comments for the Author):

Comment 30: This is a well-prepared presentation for an extensively executed study, almost comprehensively on the multiple omics' approaches applied to study an important regulator, FERONIA. I'd pass on the detail assessment of the omics' experiments and data analysis. I did take a look at the S-data and the list of genes etc., but would like to emphasize that ready accessibility to data repository should be required for this type of study. I hope the review and editorial process could ensure that is the case. As resource papers go, this paper is also rich in experimental details, surely benefitting also from the fact that many phenotypes in *fer* mutants are drastic and tractable. I find that overall this is an imminently publishable work.

Response 30: We thank the reviewer for the constructive assessment. We included the link for data repository in the revised manuscript (lines 585-587, 608-612).

Comment 31: I have one aspect that I consider important that this work underserves but is in fact in an excellent position to provide the information. When reading through the manuscript, the entire time, especially in the phospho-protein analysis, I kept thinking the authors have in their hands an excellent resource to tackle the question of what the kinase aspect of FER is crucial for in vivo. In the literature, there are conflicted findings, some phenotypes are said to depend on, and others not depend on, or it is only dependent on how much kinase dead-FER is over-expressed. Here, there is a

list, some important regulators themselves, to test whether the pathways they control is dependent on FER-KD.

This thought was especially so when reading the final experiment related to ABA sensitivity. My first thought was as above, whether the TFs listed (L345-348) are direct targets of FER. The work very nicely went on to show that they are, and also in vitro, but fell short of a complementation assay using the FER-KD complemented lines to see if the ABA-related phenotypes are restored, or NOT.

> **Response 31**: These are excellent suggestions. We have obtained complemented lines from Prof. Sally Assmann, one WT *FER-GFP* complemented line and three lines with different expression levels of *FER^{K565R}-GFP* (Chakravorty, et al. 2018). High levels of FER^{K565R} was able to complement the growth phenotype of *fer-4* to different degrees. We examined ABA responses by measuring cotyledon greening of WT, *fer*, *fer FER-GFP*, *fer FER^{K565R}-GFP* (three lines) on control and plates containing 1 μM ABA. Consistent with the growth phenotypes (Chakravorty, et al. 2018), the WT *FER-GFP* line almost completely complemented the hypersensitivity of *fer-4* to ABA during cotyledon greening. *FER^{K565R}-GFP* partially complemented the hypersensitivity of *fer-4* to ABA, and lines with higher FER^{K565R}-GFP levels showed higher degree of complementation (Figure 7D and 7E). The data suggest that FER kinase activity is important for FER-mediated ABA response during cotyledon greening (Lines 349-355).
>
> Another interesting point is that high levels of FER^{K565R} showed partial complementation in vegetative growth (Chakravorty, et al. 2018), ABA response (Figure 7D and 7E) and ER body/IG gene expression (Figure 3F and Figure 6B, Lines 260-275 and 306-318), suggesting that FER^{K565R} might have residual kinase activity. We are also able to demonstrate residual kinase activity of FERK^{K565R} through autophosphorylation in in vitro kinase assay. In Figure 2D, we compared the autophosphorylation of FERK to that of different amount of FERK^{K565R}, and found that FERK^{K565R} has about 100- to 200-fold lower kinase activity in vitro, as 100- to 200-fold more FERK^{K565R} protein is needed to achieve similar level of autophosphorylation as WT (Lines 166-178).

Comment 32: There had been several previous studies that were heavily built upon FER's kinase activity biochemically [e.g. cited in Intro, e.g. L62-68; and the phosphor-omics result, e.g. pg. 10, L205-206, what about the ER body phenotype, is it K-dependent?], but the in vivo consequences were not addressed adequately or at all. I think this would be a good place to provide some clarity on the issue, or even just to reaffirm previous conclusions. The authors probably already have the lines in hand (they should be available from many labs), many of the phenotypes are very easy to analyze. The efforts would have done the FERONIA community a huge favor.

> **Response 32**: The expression ER body genes is a reliable marker for ER body phenotype. We checked the transcript levels of ER body genes *NAI1*, *NAI2* and *PYK10* by qPCR in WT, *fer*, *fer FER-GFP*, two lines of *fer FER^{K565R}-GFP*. Similar to growth phenotype (Chakravorty, et al. 2018) and ABA response, *FER^{K565R}-GFP* was able to partially complement *fer-4* while *FER-GFP* completely complemented *fer-4* in the expression of these ER body genes (Figure 3F, Lines 260-275). The data suggest that FER kinase activity is important for FER-mediated ER body formation.

Other issues are minor:

Comment 33: L52: Lin et al. 2018

Response 33: Added, now in line 60.

Comment 34: Pg. 5, L83, 4.5 weeks; methods say 5 weeks. This is not so important, I was in fact only curious to look because the text distinctly refers to see methods. But my thoughts were more at ~5 weeks, good-looking *fer* plants (which in this case they were) are actually adequately robust, so perhaps the profiling - drastically different enough --- at younger stages or different stages might provide additional insights that directly reflect the severity of phenotypes. This thought was later reinforced when reading the ER body study. The authors might wish to select some key genes and do an expression analysis during crucial developmental stages, such as during embryogenesis when cotyledons develop and early seedling stages etc.

L150 FER and ER body section - I could have missed and that it was mentioned somewhere, the physiological/cell biological roles served by the ER bodies. My notes, around L190, says it would be good to have that background and to have a discussion of the results just presented with a functional context connect to *fer* at 10 d. This is also where I thought having a development profile of the expression pattern of the genes involved would be a nice complement, given the ER body phenotype differences in *fer* seen in the two developmental stages. Along that line, what is the seedling phenotype of *nai1*?

Response 34: We have generated RNA-seq data for 10-day-old whole seedlings of WT and *fer-4* (Supplemental Data Set S4). It has significant overlap with RNA-seq data from the 4.5-week-old plants (Supplemental Figure S2B). In addition, all the ER body genes have increased RNA levels in the seedlings (Figure 3A), suggesting that FER regulation of ER body is consistent at different developmental stages.

We also checked the expression of *NAI1*, *NAI2*, and *PYK10* by qPCR in 3-week-old plants, and the expression pattern is very similar to that of 10-day-old seedlings and the 4.5-week-old plants (Figure 3C).

The *nai1* mutant seedlings and adult plants do not have visible growth phenotypes compared to WT under normal growth conditions.

Comment 35: L205, I understand the motivation to have a simple model and hypothesis present (Fig. 3F) so the rationale of the experiments come through easier. But I felt that the linear relationship as stated/present was premature and probably way over-simplified with the results shown thus far. It might be good just to leave it until more of the results were collected and interpreted collectively.

Response 35: We removed the linear models from Fig3, Fig4 and Fig 7.

Comment 36: L250 TOR studies were nicely done.

L322, the conclusion that "*TOROE* has elevated TOR kinase activity" is over-stated, being at 0.45 while the mutant is only 0.42. I think generally since the basal level of the S6K response is already extremely low (the difference from WT is striking), to monitor the responses to TOR inhibitors would be extremely difficult, or probably not so meaningful either. L301, the results on root growth responses also support this, *fer* being more hyposensitive to AZD ($p<0.05$) is really marginal at best.

I suggest to focus the discussion on the basal difference and, if possible, relate that to logically connectable phenotypes.

> **Response 36**: We removed the FER-TOR data (we plan to publish in a separate manuscript) and focused on FER-ER body/IG biosynthesis and FER-ABI5 in the revision as suggested by Reviewers #1 and #3.

Comment 37: L345-352. On first read, this segment is not very clear as to whether the TFs (Line 345-348 are direct targets of FER) and then the results of the CHIP seq studies referred to what. But later sections clarified my first question. But see my comment above.

> **Response 37**: The ChIP seq data from Song et al, 2016 was used to show that FER-regulated genes have statistically significant overlaps with the target genes of these TFs (Supplemental Fig S12A and Data Set S5), which supports that FER functions through these TFs.

Reviewer #3 (Comments for the Author):

Comment 38: This manuscript reports transcriptomics, proteomics and phosphoproteomics of WT and *fer* mutant. Through the omics data analysis, the authors identified four biological processes for further investigation, i.e., ER body formation, glucosinolate biosynthesis, autophagy and ABA response during germination. Genetic approaches were mainly used for establishing that FER is a negative regulator for all the four processes. Here are some concerns for the authors to consider:

The WT and mutant have distinct growth and developmental differences. Omics will reveal many pleiotropic changes in molecules at different levels. An inducible system would help to minimize the pleiotropic effects and identify direct changes related to FER function.

> **Response 38**: We thank the reviewer for the comments. We agree that loss-of-function of FER can cause pleiotropic changes, which prompted us to identify specific downstream effectors of FER in different biological processes. The *abi5* mutant largely complemented *fer-4*'s hypersensitivity to ABA during cotyledon greening (Figure 7B and 7C), and the *nai1* mutant largely complemented *fer-4*'s constitutive ER body phenotype (Figure 3D and 3E), which suggested that these two processes are specifically regulated by the FER signaling pathway.

Comment 39: For each of the four processes, genetics experiments showed correlation, but not direct evidence for FER function in many cases. For example, how does FER negatively regulate NAI1 and NAI2? Does FER phosphorylate NAI1 and/or NAI2?

> **Response 39**: *NAI1* transcript level is increased in *fer-4*, and protein and phosphorylation not detected. It is likely that FER regulates intermediate proteins that in turn regulate NAI1. *NAI2* is a direct target of NAI1 (Sarkar et al 2021).

Comment 40: In addition, glucosinolate biosynthesis and regulation have been well-studied. If the authors like to establish FER and NAI1 functions in indolic glucosinolate biosynthesis, a lot more

critical experiments need to be carried out (e.g., causal relationship with the well-known Myb34, 51 and 122, as well as P450s).

Response 40: We thank the reviewer for the suggestions. The detailed mechanisms of how FER and NAI1 function to regulate indolic glucosinolates biosynthesis, and if and how FER interacts with myb34, 51, 122 and P450s to regulate IG biosynthesis will be interesting for future studies. In this manuscript, we used the multiomics data analyses to provide a framework for the future studies.

Comment 41: The genetic results beg more biochemistry and orthogonal experiments. Does FER interact with TOR and phosphorylate it?

Response 41: We removed the FER-TOR study and focused on FER-ER body/IG biosynthesis and FER-ABI5 in the revision as suggested also by Reviewer #1.

Comment 42: Although in vitro kinase assay showed activity of phosphorylation, in vivo validation is essential. For example, do the seven out of nine proteins interact with FER in vivo? Does FER phosphorylate ABI5?

Response 42: We thank the reviewer for the suggestions.

From the in vivo phosphoproteomic data analysis, we detected phosphorylation sites/peptides from the nine selected proteins with decreased phosphorylation, which suggests that FER phosphorylates them in vivo (Figure 2A).

The in vitro kinase was used to validate the in vivo findings, which confirmed that seven of the nine proteins were directly phosphorylated by FER (Figure 2B). We also mutated the detected phosphorylation site in ABI5 from serine to alanine and the phosphorylation level was decreased (Figure 7I), suggesting that FER phosphorylates ABI5 at S145.

We further showed the interactions of FER with some of the substrates, SHOU4, FBH3, ABI5 via BiFC (Figure 2E, Supplemental Figure S3C and S3D). Interactions of FER and SHOU4, FBH3 were also confirmed with Co-immunoprecipitation (Figure 2F and 2G, Lines 179-185).

Comment 43: The authors should have focused on just one of the four processes and conducted detailed mechanistic studies. Current work showed some genetic relationship and correlations, but still have a lot of gaps.

Response 43: We removed the FER-TOR data and focused on FER-ER body/IG biosynthesis and FER-ABI5 in the revision.

Comment 44: All the proteomics data need to be deposited at ProteomeXchange for public access.

Response 44: We provide the link in the manuscript (lines 585-587).

Comment 45: Glucosinolate method needs to be detailed. How were the glucosinolates identified and quantified? What are the standard compounds?

Response 45: We expanded the method and added a new reference in lines 704-708.

TPC2021-LSB-00634R1	2nd Editorial decision – *Accept (minor revisions)*	Feb. 15, 2022

We have received reviews of your manuscript entitled "Integrated Omics Reveal Novel Functions and Underlying Mechanisms of FERONIA Receptor Kinase in *Arabidopsis thaliana*." On the basis of the advice received, the board of reviewing editors would like to accept your manuscript for publication in The Plant Cell.

This acceptance is contingent on revision based on the comments of our reviewers. In particular, please consider the following textual editing: the general introduction on the importance of the FER CrRLK1L family in a broad plant biology field, biological connections of ER, IG, and cotyledon greening, a precise description of *fer* mutant growth phenotypes at ten-day-old and four-week-old stages, and consistent description on cotyledon greening.

TPC2021-LSB-00634R2	2nd Revision received	Feb. 22, 2022

-- Reviewer comments (with **Authors responses in** blue):

Summary of Response

Dear editor and reviewers,

We would like to thank you for your consideration of accepting our manuscript for publication and for the critical and constructive comments/suggestions and the favorable assessments of the revised manuscript. We have added the importance of the FER receptor kinase in a broad plant biology field in the introduction. FERONIA receptor kinase plays critical roles in mediating plant growth and stress responses. FER studies will provide an important paradigm in the plant biology field to study receptor kinase signaling and how plants balance growth and stress in response to environmental conditions. We wanted to clarify that the goal of this study was not to make connections of ER-body, IG biosynthesis and cotyledon greening, rather validate the findings of the integrated omics analyses. We also added the plant photos of 4.5-week- and 10-day-old plants, changed “seed germination” to “cotyledon greening”, and made edits as appropriate. The detailed responses are summarized below and corresponding changes are highlighted in BLUE in the manuscript. We numbered the comments and responses to facilitate tracking.

Reviewer comments:

Reviewer #1 (Comments for the Author):

In the revised manuscript, the authors addressed my major concerns and provided reasonable explanations for others. However, there are still a few points that I consider important to be addressed.

Comment 1: The description of title is not appropriate. In the manuscript, many methods other than integrated omics were used, such as genetics, cell biology, and biochemistry.

Response 1: Our justification of the title: the "integrated omics" was essential for the new findings of the study so is highlighted in the title. The genetic, molecular and cell biological, and biochemical experiments were performed to validate these findings.

Comment 2: In Introduction and Discussion section, the importance of studying of FER function should be reflected: what is the significance of studying receptor kinases in plants or biology field? What is the research value of the CrRLK1L family?

Response 2: The significance of the FER study is added in BLUE in introduction (Lines 80-85).

Comment 3: It was better to make a connection in ER, IG, and cotyledon greening: the author verified these three parts due to the intrinsic connection of them or some other reasons?

Response 3: We analyzed them because we identified ER-body and IG biosynthesis in Gene Ontology analysis as novel functions of FER. We also identified that ABI5 is a FER substrate and proposed a molecular mechanism of FER regulation in cotyledon greening. Our goal of this study was not to make connections of different biological processes.

Comment 4: Line 52：Chen et al., 2016 first reported that *fer* mutants display hypersensitivity to high concentrations of NaCl. Please refer to it.

Response 4: We added it.

Comment 5: Fig. 1A, the order of transcriptome, proteome, and phosphorylome was shown according to the logical description in the manuscript.

Response 5: We changed the order in Figure 1A.

Comment 6: Line 122-126: The authors found that the up-regulated genes in the *fer* mutant were enriched in hormone pathways such as JA, ABA, and ethylene, which confirmed the previous conclusion FER negatively regulates biological pathways such as JA, ABA, and ethylene. It was not accurate. Here, it could not judge that whether FER is positive or negative regulation in these hormone pathways and just draw a conclusion that FER is involved in these signaling pathways. For example, some upregulated genes that are negatively regulated above pathways could also be enriched. The same problems were applied to other GO analysis.

Response 6: We changed "inhibits" to "regulates".

Comment 7: Line 149: why the hypo-phosphorylated proteins in *fer* mutant are potential FER substrates?

Response 7: We expect that FER substrates are hypo-phosphorylated (decreased phosphorylation) in *fer-4* mutant due to the loss-of function of FER.

Comment 8: Line 181: "FER showed interactions with SHOU4, FBH3 and ABI5 in the cytoplasm." However, the Fig. 2E showed that FER interacted with these proteins in the plasma membrane. Please confirmed it.

Response 8: We removed "in the cytoplasm".

Comment 9: The result of FERKD protein purification was still not shown and needed to explain it.

Response 9: The quality and quantity of the mFERK protein along with WT FERK are shown by western blotting using anti-FER antibody in supplemental Figure 9B. We included the Coomassie blue staining of the proteins below. The mFERK protein purification yielded large amount of protein, much more than WT FERK using same number of cells. That was why we used western blotting to estimate the amount of proteins we used for in vitro kinase assay.

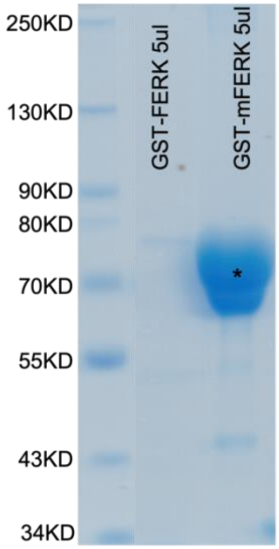

Comment 10: Line 332: FER phosphorylates ABI5, however, ABI5 are hypo-phosphorylated in *fer*?

Response 10: ABI5 is a FER substrate, and it is expected to be hypo-phosphorylated (i.e. decreased phosphorylation) in the *fer-4* mutant.

Comment 11: The author was still not supplementing the genetic materials of fer/ABI5S145A-OE, which further confirms the regulation of FER on the stability of ABI5.

Response 11: Since ABI5S145A is more stable than ABI5 (Figure 7I), and ABI5 is more stable in the *fer-4* mutant (Figure 7G), it would not be very informative to compare the stability of ABI5 and ABI5S145A in *fer-4* background by making the *fer ABI5S145A-OE* line.

Comment 12: It would be necessary to have molecular mass markers in all WB figures as shown in Fig. 2B.

Response 12: We added them in all WB figures.

Comment 13: The abbreviation such as didn't and It's should not be used, and correct them.

Response 13: We corrected them through the text.

Reviewer #2 (Comments for the Author):

Comment 14: I very much appreciate the authors' effort in the revisions, in particular the extent of analysis carried out for the FER kinase involvement in the phenotypes under study. I think it is a great service to the community.

I only have one query:

The authors mentioned in response to Rev. #1:

"The reason that 4.5-week-old plants were used for the multi-omics was that *fer-4* shows the strongest growth phenotype at this age. " and later to my or Rev. #3's comments that 4.5-week- and 10-day-old seedling transcriptomes have considerable overlap.

I think just the "overlap" is adequate justification, and their experiments justify the data that relate to developmental time. However, that 4.5 weeks *fer* plants in their hands shows the strongest growth phenotype is a curious comment. *fer* is very affected by growth conditions --including light intensity and humidity, if in their growth conditions there are clear distinctions for 10-day-old and 4.5-week-old *fer* plants in terms of severity in growth phenotype, it would be good to show a picture of both.

> **Response 14**: Thank you for the comment. The comparison of the differentially regulated genes in *fer-4* mutant between 4.5-week-old plants and 10-day-old seedlings is shown in Figure S2B. In our last response to reviewers, the "significant overlap" was meant to refer to ER body and glucosinolate genes. We did not compare genes involved in other pathways.
>
> We provide plant pictures of 4.5-week-old plants and 10-day-old seedlings in supplemental Figure S2C-S2D, and added description of the plant growth phenotypes in results highlighted in BLUE (Lines 103-105).

Reviewer #3 (Comments for the Author):

Comment 15: In this revision, the authors have address most of my concerns. The added experiments and data are important for the mechanistic understanding of FER functions. The co-regulation between ER body and glucosinolates may also be due to the fact that glucosinolate enzymes reside on ER membranes. The author may consider this point. In addition, in Figure 8 and other places, germination was still used. Germination and cotyledon greening are different. Please make correction to be consistent in the manuscript. Also there are typos and errors. For example, in lines 414, 500, 696 (freeze) etc. In addition, please italicize all the species names. These can be taken care of in the next round of revision or during the author proof.

> **Response 15**: We thank the reviewer for the insightful comment on the co-regulation of ER body and glucosinolates. However, in addition to glucosinolate enzymes, many other enzymes including flavonoid and anthocyanin biosynthesis enzymes are also associated with the ER membrane that are

NOT co-regulated with ER bodies. We think that the association of glucosinolate enzymes with the ER membrane might not be the main reason for the co-regulation.

We changed “germination” to cotyledon greening” in Figure 8 and figure legend, and also corrected some typos.

**TPC2021-LSB-00634R2**	**2nd Editorial decision – *Accept***	**Feb. 28, 2022**

We are pleased to inform you that your paper entitled "Integrated Omics Reveal Novel Functions and Underlying Mechanisms of FERONIA Receptor Kinase in *Arabidopsis thaliana*" has been accepted for publication in The Plant Cell, pending a final minor editorial review by journal staff. At this stage, your manuscript will be evaluated by a Science Editor with respect to its presentation of scientific content, compliance with journal policies, and presentation for a broad readership.

**Final acceptance from Science Editor**	**Apr. 08, 2022**
